# Evaluating the Test-Negative Design for COVID-19 Vaccine Effectiveness Using Randomized Trial Data: A Secondary Cross-Protocol Analysis of 5 Randomized Clinical Trials

**DOI:** 10.1001/jamanetworkopen.2025.12763

**Published:** 2025-05-28

**Authors:** Leah I. B. Andrews, M. Elizabeth Halloran, Kathleen M. Neuzil, Lars van der Laan, Yunda Huang, Jessica Andriesen, Mayur Patel, Leigh H. Fisher, Holly Janes, Nadine Rouphael, Stephen R. Walsh, Deborah A. Theodore, Hong-Van Tieu, Magdalena Sobieszczyk, Hana M. El Sahly, Lindsey R. Baden, Ann R. Falsey, Thomas B. Campbell, Colleen F. Kelley, Catherine Mary Healy, Lilly Immergluck, Benjamin Luft, Ian Hirsch, Guy de Bruyn, Carla Truyers, Frances Priddy, Kelsey M. Sumner, Brendan Flannery, Dean Follmann, Peter B. Gilbert

**Affiliations:** 1 Department of Biostatistics, School of Public Health, University of Washington, Seattle; 2 Fred Hutchinson Cancer Center, Seattle, Washington; 3 Fogarty International Center, National Institutes of Health, Bethesda, Maryland; 4 Department of Statistics, College of Arts and Sciences, University of Washington, Seattle; 5 Division of Infectious Diseases, Department of Medicine, Emory University School of Medicine and the Grady Health System, Atlanta, Georgia; 6 Division of Infectious Diseases, Brigham and Women’s Hospital, Boston, Massachusetts; 7 Division of Infectious Diseases, Department of Medicine, Vagelos College of Physicians and Surgeons, New York-Presbyterian–Columbia University Irving Medical Center, New York, New York; 8 Lindsley F. Kimball Research Institute, New York Blood Center, New York, New York; 9 Department of Molecular Virology and Microbiology, Baylor College of Medicine, Houston, Texas; 10 Division of Infectious Diseases, Department of Medicine, University of Rochester, Rochester, New York; 11 Division of Infectious Diseases, University of Colorado Anschutz Medical Campus, Aurora; 12 Division of Infectious Disease, Department of Pediatrics, Baylor College of Medicine, Houston, Texas; 13 Section of Pediatric Infectious Diseases, Department of Pediatrics, University of Chicago, Chicago, Illinois; 14 Department of Medicine, Stony Brook University, Stony Brook, New York; 15 Biometrics, Vaccines and Immune Therapies, BioPharmaceuticals R&D, AstraZeneca, Cambridge, United Kingdom; 16 Sanofi Vaccines Research & Development, Swiftwater, Pennsylvania; 17 Janssen Global Commercial Strategy Organization RWE, Beerse, Belgium; 18 Moderna Inc, Cambridge, Massachusetts; 19 Influenza Division, Centers for Disease Control and Prevention, Atlanta, Georgia; 20 National Institute of Allergy and Infectious Diseases, Bethesda, Maryland

## Abstract

**Importance:**

The test-negative design (TND) has been widely used to assess postmarketing COVID-19 vaccine effectiveness but requires further evaluation for this application.

**Objective:**

To determine whether the TND reliably evaluates vaccine effectiveness against symptomatic COVID-19 using placebo-controlled vaccine efficacy randomized clinical trials (RCTs).

**Design, Setting, and Participants:**

This secondary cross-protocol analysis constructed TND study datasets from study sites in 16 countries across 5 continents using the blinded phase cohorts of 5 harmonized phase 3 COVID-19 Prevention Network RCTs: COVE (Coronavirus Vaccine Efficacy and Safety), AZD1222, ENSEMBLE, PREVENT-19 (Prefusion Protein Subunit Vaccine Efficacy Novavax Trial COVID-19), and VAT00008. Participants included adults who received the intended number of doses, experienced COVID-19–like symptoms, and obtained SARS-CoV-2 testing. Start dates ranged from July 27, 2020, to October 19, 2021; data cutoff dates ranged from March 26, 2021, to March 15, 2022. Statistical analysis was performed from May 11, 2023, to February 25, 2025.

**Interventions:**

Participants received vaccines consisting of messenger RNA-1273 (COVE; 2 doses 28 days apart), ChAdOx1 nCoV-19 (AZD1222; 2 doses 28 days apart), Ad26.COV2.S (ENSEMBLE; 1 dose), NVX-CoV2373 (PREVENT-19; 2 doses 21 days apart), CoV2 preS dTM-AS03 (VAT00008; D614) (2 doses 21 days apart), or CoV2 preS dTM-AS03 (D614 plus B.1.351) (VAT00008; 2 doses 21 days apart) or placebo.

**Main Outcomes and Measures:**

Main outcomes were symptomatic COVID-19 according to each trial’s primary efficacy definition and the Centers for Disease Control and Prevention definition. Vaccine effectiveness was estimated using targeted maximum likelihood estimation under a semiparametric logistic regression model and ordinary logistic regression. Noncase exchangeability, a core TND assumption for unbiased estimation, was also assessed by estimating vaccine efficacy against non–COVID-19 illness.

**Results:**

Among the 12 157 participants included in the analysis, mean (SD) age was 45 (15) years, 6414 were female (53%), 5858 were vaccinated (48%), 2835 experienced primary COVID-19 (23%), and 2992 experienced Centers for Disease Control and Prevention–defined COVID-19 (25%). TND vaccine effectiveness estimates were concordant with RCT vaccine efficacy estimates (concordance correlation coefficient, 0.86 [95% CI, 0.58-0.96] for both outcomes). The semiparametric method had 48% smaller variance estimates than ordinary logistic regression. Noncase exchangeability was generally supported with a median vaccine efficacy against non–COVID-19 illness of 7.7% (IQR, 2.7%-16.8%) across trial cohorts and most 95% CIs including 0.

**Conclusions and Relevance:**

In this cross-protocol analysis, the TND provided reliable inferences on COVID-19 vaccine effectiveness in health care–seeking populations for multiple vaccines and symptom definitions when confounding and selection bias were absent. A machine-learning approach for robust confounding control in postmarketing TND studies was also introduced.

## Key Points

**Question:**

Does the test-negative design (TND) reliably assess COVID-19 vaccine effectiveness when using data from placebo-controlled vaccine efficacy randomized clinical trials (RCTs)?

**Findings:**

In this secondary cross-protocol analysis of 5 US government-sponsored phase 3 COVID-19 RCTs with 12 157 participants, TND vaccine effectiveness estimates derived from a robust machine-learning logistic regression approach were concordant with RCT vaccine efficacy estimates (concordance correlation coefficient, 0.86).

**Meaning:**

These findings suggest that in settings that lack confounding and selection bias, the TND can reliably evaluate COVID-19 vaccine effectiveness in a health care–seeking population; however, postmarketing TND studies must address these issues.

## Teaser

This secondary cross-protocol analysis assesses 5 phase 3 COVID-19 Prevention Network randomized clinical trials as test-negative design studies to evaluate how various sampling methods, symptom definitions, statistical approaches, and study populations are associated with the accuracy and precision of symptomatic COVID-19 vaccine effectiveness estimates.

## Introduction

In 2020, the US government established the COVID-19 Prevention Network (CoVPN) to develop safe and effective vaccines against COVID-19, a contagious respiratory disease caused by SARS-CoV-2. The resulting phase 3 placebo-controlled randomized clinical trials (RCTs) were conducted in diverse settings and provided strong evidence to authorize or approve multiple COVID-19 vaccines.^1,2,3,4,5,6,7^ To inform vaccine recommendations, regimen updates, and strain selection, postmarketing studies must continually evaluate variant-updated vaccine effectiveness against multiple SARS-CoV-2 variants and end points and in groups underrepresented in COVID-19 RCTs. It is challenging to conduct RCTs to meet postmarketing needs.

Test-negative design (TND) studies were among the first postmarketing studies to assess COVID-19 vaccine effectiveness and have been widely implemented as practical resource-efficient observational study designs.^8,9,10,11,12,13^ A COVID-19 TND study enrolls individuals who meet a COVID-19 symptom definition and seek SARS-CoV-2 testing. Vaccine effectiveness is estimated by comparing vaccination status between individuals with positive test results for SARS-CoV-2 (cases) and those with negative test results for SARS-CoV-2 (noncases), after adjusting for potential confounders. Symptoms among noncases are caused by non–COVID-19 illnesses, such as other respiratory illnesses or allergies. The TND reduces confounding and selection bias from health care–seeking behavior, or the propensity to seek care when ill, by assuming all TND participants have identical health care–seeking behavior (eFigure 1 in Supplement 1). Most TND analyses either limit inferences to a health care–seeking population or assume no effect modification by health care–seeking behavior and generalize to the entire population.^14,15,16,17,18^ TND studies are resource efficient because they recruit noncases identically to cases, collect all information in a single visit, and enroll a high proportion of COVID-19 cases. The TND has been implemented to assess vaccine effectiveness against other diseases, including pneumococcal disease, influenza, and rotavirus.^19,20^

Although the TND is already in use to assess COVID-19 vaccine effectiveness, it should be evaluated for this application.^15,16^ Previous studies have investigated how the TND may be subject to bias from confounding,^15,16,17,21,22,23,24,25,26^ selection mechanisms,^15,16,17,21,24,25,27,28,29,30^ vaccination status misclassification,^16,31^ case status misclassification,^16,17,29,32,33^ viral interference,^21,27^ and the choice of study end point.^17,21,26^ Most studies involved theory and/or simulations, though 1 study compared the TND with an emulated target trial for COVID-19.^24^ However, no studies to date have evaluated the TND in a COVID-19 RCT setting with blinded and randomized vaccination, frequent symptom reporting and testing, and a known RCT ground truth. Assessing how reliably TND vaccine effectiveness estimates approximate RCT vaccine efficacy estimates in this setting can isolate additional issues when studying COVID-19 vaccine effectiveness in health care–seeking populations.

TND and RCT estimates derived in a setting without confounding or selection bias could differ for several reasons. For interpretable unbiased results, most TND analyses require noncase exchangeability, the assumption that vaccination status is not associated with meeting the noncase definition (ie, meeting the symptom definition and SARS-CoV-2 negative test results) in health care–seeking individuals, conditional on measured covariates.^15,19,34,35,36^ Noncase exchangeability may be violated if a COVID-19 vaccine affects non–COVID-19 illnesses or if confounders of COVID-19 vaccination and non–COVID-19 illness are not adjusted for in TND analyses.^15,19,23,34,35,36^ The former condition can be assessed using RCT data, as shown previously for influenza,^37^ rotavirus,^38^ cholera,^39^ and typhoid.^40^ Differences could also arise from case status misclassification due to imperfect SARS-CoV-2 diagnostic tests.^13,41^ Moreover, differences may occur from applying different TND sampling methods when individuals have multiple eligible SARS-CoV-2 tests during the study.^8,10,11,12^ Selecting one SARS-CoV-2 test per individual provides valid confidence intervals under standard statistical methods, but the choice of test result could induce bias if many individuals experience COVID-19 and non–COVID-19 illness.^37^ Including all SARS-CoV-2 tests increases the number of tests but may affect estimators’ variance estimates.

In postmarketing TND settings, confounding from characteristics such as age, comorbidities, infection history, and calendar date^22^ exists and is typically addressed using ordinary logistic regression^8,9,10,11,12^ or matching and conditional logistic regression.^42,43^ Misspecification of confounding or accounting for too many potential confounders can bias estimates.^44,45,46^ Matching is more resource intensive for studying multiple symptom definitions or pathogens in the same TND study and may be less efficient than covariate adjustment through regression.^47^ Recently, a doubly robust 1-step estimator involving machine-learning for covariate-adjustment has been developed.^36^

In this secondary cross-protocol analysis, we reanalyzed 5 phase 3 CoVPN RCTs as TND studies to evaluate how various TND sampling methods, symptom definitions, statistical approaches, and study populations are associated with the accuracy and precision of symptomatic COVID-19 vaccine effectiveness estimates in a setting where most sources of bias are known to be controlled. We estimated TND vaccine effectiveness from each TND study dataset using a novel semiparametric logistic regression approach that flexibly accounts for confounding using machine learning^48^ and ordinary logistic regression. We compared TND estimates with RCT estimates and assessed noncase exchangeability violations for all trial cohorts.

## Methods

### Data Sources

We analyzed data from 5 phase 3 CoVPN RCTs: COVE (Coronavirus Vaccine Efficacy and Safety) from Moderna Inc (CoVPN3001),^1^ AZD1222 from AstraZeneca/Oxford (CoVPN3002),^2^ ENSEMBLE from Janssen/Johnson & Johnson (CoVPN3003),^3^ PREVENT-19 (Prefusion Protein Subunit Vaccine Efficacy Novavax Trial COVID-19) from Novavax Inc (CoVPN3004),^4^ and VAT00008 from Sanofi/GSK (CoVPN3005).^5,6^ All trial protocols and amendments were approved by the applicable local ethics committees and/or institutional review boards or a central institutional review board as described in the final blinded phase analysis publications.^1,2,3,4,5,6^ Trial participants provided written informed consent before enrollment.

We defined 10 adult trial cohorts from the final blinded phase of these RCT primary efficacy analysis cohorts (Table and eTable 1 and eFigure 2 in Supplement 1).^1,2,3,4,5,6,7^ Most trial cohorts were restricted to participants classified as SARS-CoV-2 negative at baseline (BN), as previously defined (eTable 1 in Supplement 1),^1,2,3,4,5,6^ to indicate no known prior SARS-CoV-2 infections. We analyzed the ENSEMBLE study as 3 trial cohorts defined by Latin American, South African, and US regions, given distinct circulating SARS-CoV-2 lineages.^3^ The VAT00008 RCT was conducted in 2 stages: stage 1 compared monovalent vaccine vs placebo and stage 2 compared bivalent vaccine vs placebo. Most enrolled participants in each stage were SARS-CoV-2 positive at baseline (BP). Since prior infection is an immunity-conferring event and vaccine efficacy was greater in the BP than the BN cohort,^5,6^ we analyzed these cohorts separately for stages 1 and 2.

**Table.** Phase 3 COVID-19 Prevention Network Placebo-Controlled Randomized Clinical Trial Cohorts Analyzed^a^

Trial cohort (sponsor)	Intervention	Location	Study population	Baseline SARS-CoV-2 status	Dates	End points	Start of follow-up
COVE BN (Moderna Inc)^1^	mRNA-1273 or placebo (2 doses 28 d apart)	US	Adults aged ≥18 y with no known history of SARS-CoV-2 and high risk for SARS-CoV-2 and/or its complications	PCR negative and/or seronegative	July 2020 to March 2021	Primary COVID-19 (mild, moderate, or severe-critical); CDC COVID-19	14 d After dose 2
AZD1222 BN (AstraZeneca/Oxford)^2^	ChAdOx1 nCOV-19 or placebo (2 doses 28 d apart)	Chile, Peru, US	Adults aged ≥18 y with no previous laboratory-confirmed SARS-CoV-2 and high SARS-CoV-2 risk who were healthy or had a stable chronic disease	Seronegative	August 2020 to July 2021	Primary COVID-19 (mild, moderate, or severe-critical); CDC COVID-19	15 d After dose 2
ENSEMBLE Latin America BN (Janssen/Johnson & Johnson)^3^	Ad26. COV2.S or placebo (1 dose)	Argentina, Brazil, Chile, Colombia, Mexico, Peru	Adults aged ≥18 y without conditions associated with high SARS-CoV-2 risk who were healthy or had a stable chronic disease	PCR negative or seronegative	September 2020 to July 2021	Primary COVID-19 (moderate or severe-critical); CDC COVID-19	14 d After dose
ENSEMBLE South Africa BN (Janssen/Johnson & Johnson)^3^		South Africa					
ENSEMBLE United States BN (Janssen/Johnson & Johnson)^3^		US					
PREVENT-19 BN (Novavax Inc)^4^	NVX-CoV2373 or placebo (2 doses 21 d apart)	Mexico, US	Adults aged ≥18 y with no previous laboratory-confirmed SARS-CoV-2 and high SARS-CoV-2 risk who were healthy or had a stable chronic disease and without immunosuppression	PCR negative and seronegative	December 2020 to June 2021	Primary COVID-19 (mild, moderate, or severe-critical); CDC COVID-19	7 d After dose 2
VAT00008 Stage 1 BN (Sanofi/GSK)^5^	CoV2 preS dTM-AS03 (D614) or placebo (2 doses 21 d apart)	Colombia, Ghana, Honduras, India, Japan, Kenya, Nepal, US	Adults aged ≥18 y, previously unvaccinated with no interest in receiving one of the approved/authorized vaccines	NAAT negative and seronegative	May 2021 to January 2022	Primary COVID-19; CDC COVID-19	14 d After dose 2
VAT00008 stage 1 BP (Sanofi/GSK)^5^				NAAT positive or seropositive			
VAT00008 stage 2 BN (Sanofi/GSK)^6^	CoV2 preS dTM-AS03 (D614 plus B.1.351) or placebo (2 doses 21 d apart)	Colombia, Ghana, India, Kenya, Mexico, Nepal, Uganda, Ukraine		NAAT negative and seronegative	October 2021 to March 2022		
VAT00008 stage 2 BP (Sanofi/GSK)^6^				NAAT positive or seropositive			

Abbreviations: BN, baseline SARS-CoV-2 negative; BP, baseline SARS-CoV-2 positive; CDC, Centers for Disease Control and Prevention; COVE, Coronavirus Vaccine Efficacy and Safety; mRNA, messenger RNA; NAAT, nucleic acid amplification test; PCR, polymerase chain reaction; PREVENT-19, Prefusion Protein Subunit Vaccine Efficacy Novavax Trial COVID-19.

^a^ Our study refers to individuals randomized to receive the vaccine or placebo intervention as vaccinated or unvaccinated, respectively.

COVID-19 vaccination status was defined as in the final blinded phase of each RCT’s primary efficacy analysis cohort with no missingness (Table and eTable 1 in Supplement 1).^1,2,3,4,5,6^ We refer to participants randomized to the vaccine as vaccinated and those randomized to the placebo intervention as unvaccinated. Participants’ age, sex, race and ethnicity, region, and comorbidities were collected at RCT enrollment, with some missing data for race and ethnicity (eTable 2 and eMethods in Supplement 1).

All trials instructed participants to monitor and report symptoms, which would trigger SARS-CoV-2 polymerase chain reaction or nucleic acid amplification testing (NAAT). Each trial’s primary efficacy end point, or primary COVID-19, consisted of a harmonized symptom definition and virological confirmation.^1,2,3,4,5,6,7^ All trials also studied a Centers for Disease Control and Prevention (CDC) COVID-19 end point consisting of a CDC-recommended symptom definition^49,50^ and virological confirmation (eTable 1 in Supplement 1).

The follow-up periods of the trials’ blinded phase started between July 27, 2020, and October 19, 2021; cutoff was between March 26, 2021, and March 15, 2022. While most trials’ blinded follow-up occurred before the Delta and Omicron variants emerged,^7^ ENSEMBLE reported Delta COVID-19 end points in South African sites,^3^ and more than 90% of VAT00008 primary end points were from Omicron variants.^5,6^

### TND Sampling Methods

We considered participants as enrolled in a primary and/or CDC COVID-19 end point TND study if they experienced an illness episode that met the study’s symptom definition, obtained at least one eligible SARS-CoV-2 positive or negative polymerase chain reaction or NAAT result, and had complete demographic and comorbidity information (eTable 2, eFigure 2, and eMethods in Supplement 1). Eligible SARS-CoV-2 tests occurred at least 1 or 2 weeks after completing the intervention (depending on RCT protocol), within 10 days after symptom onset, after meeting the symptom definition, while blinded, and before receiving any nonstudy COVID-19 vaccinations. We selected these eligibility criteria to obtain well-defined end points, reduce selection bias, and ensure high diagnostic test accuracy.^13,28,29,41^ Under the RCT symptom reporting and testing protocols, participants had similar health care–seeking behavior and multiple eligible SARS-CoV-2 tests across and/or within illness episodes. We assumed that illness episodes triggering at least one eligible SARS-CoV-2 positive test result were caused by SARS-CoV-2 (ie, positive episodes) and illness episodes triggering only eligible SARS-CoV-2 negative test results were not caused by SARS-CoV-2 (ie, negative episodes).

To investigate SARS-CoV-2 test selection and case status misclassification in TND analyses, we applied 4 sampling methods to the enrolled participants to construct TND study datasets for each trial cohort and symptom definition (eFigure 3 in Supplement 1). In the participant-based sample without censoring for COVID-19,^37,40^ cases were participants with at least 1 positive episode, and noncases were participants with at least 1 negative episode (even if they experienced a positive episode during the study). In the participant-based sample with censoring for COVID-19,^37,40^ cases were defined identically and noncases were participants with only negative episodes (ie, one SARS-CoV-2 test per participant). In the specimen-based sample,^37,40^ positive and negative episodes were defined as cases and noncases, respectively. Given the low probability of SARS-CoV-2 reinfection within 40 weeks,^51^ participants contributed only their first positive episode and every negative episode. These 3 sampling methods assume accurate case status classification and identify the same number of cases. In the random specimen-based sample, case status was determined by randomly selecting a single eligible SARS-CoV-2 test per illness episode to assess case status misclassification that may occur if the specimen-based method were applied in a postmarketing TND study. SARS-CoV-2 tests from participants’ first positive episode and all negative episodes were considered to ensure both specimen-based samples included the same number of illness episodes and SARS-CoV-2 tests. Additional details are given in eFigure 3 in Supplement 1.

### Statistical Analysis

All analyses were conducted in R, version 4.2.2,^52^ using survival,^53,54^ causalglm,^55^ hal9001,^56^ and DescTools^57^ packages (R Program for Statistical Computing). Data were analyzed from May 11, 2023, to February 25, 2025.

#### Placebo-Controlled RCT Vaccine Efficacy

We reported primary COVID-19 vaccine efficacy estimates and 95% CIs from the trials’ final blinded phase analysis publications (eTable 1 in Supplement 1).^1,2,3,4,5,6^ We estimated CDC COVID-19 vaccine efficacy as 1 minus the hazard ratio (with Wald 95% CIs) of CDC-defined COVID-19 for vaccinated vs unvaccinated individuals using an unadjusted Cox proportional hazards model and the Efron method for handling ties.^58^

#### TND Vaccine Effectiveness

For each TND study dataset, we estimated primary or CDC COVID-19 vaccine effectiveness defined as 1 minus a causal conditional risk ratio of primary or CDC COVID-19 for vaccinated vs unvaccinated individuals in a health care–seeking population, conditional on covariates. Under a semiparametric logistic regression model, we used targeted maximum likelihood estimation of the conditional odds ratio (eMethods in Supplement 1),^48^ which can be interpreted as a causal conditional risk ratio under noncase exchangeability and standard causal assumptions.^35,36^ This method provides valid causal inference and flexible data-driven covariate adjustment. We applied the partially linear first-order smooth highly adaptive lasso^59^ for estimation, adjusting for age, sex, race and ethnicity, region, comorbidities, and 2-week testing date intervals (eTable 2 in Supplement 1)^10,20,60^ and allowing 2-way covariate interactions. We adjusted for covariates to mimic a postmarketing TND analysis that must adjust for confounding and to assess precision. We reported 2-sided Wald 95% CIs for vaccine effectiveness, transforming symmetric confidence limits about the natural logarithm conditional odds ratio, using the sample variance of the efficient influence function.

As a sensitivity analysis, we estimated vaccine effectiveness using 1 minus the conditional odds ratio from an ordinary logistic regression of vaccination status on case status, adjusted for the aforementioned covariates’ linear main effects. This conditional odds ratio can also be interpreted as a causal conditional risk ratio (eMethods in Supplement 1).^35,36^ We computed both statistical approaches’ bias, variance, and mean squared error, using the RCT estimates as the ground truth. We computed the concordance correlation coefficient with 95% CI via *z* transformation to compare RCT and TND estimates.^61^

#### Noncase Exchangeability

We assessed noncase exchangeability violations by estimating vaccine efficacy against non–COVID-19 illness using the RCT cohorts. We analyzed each RCT cohort overall and subgroups younger than 60 years vs 60 years or older. Follow-up began 1 or 2 weeks after completing the intervention, mirroring the primary efficacy analyses of the final blinded phase. We defined non–COVID-19 illness as a participant’s first negative episode from primary COVID-19–like symptoms with right-censoring by the first event of unblinding, receipt of nonstudy COVID-19 vaccination, loss to follow-up, or end of the blinded phase. We defined non–COVID-19 illness vaccine efficacy as 1 minus the hazard ratio of non–COVID-19 illness for vaccinated vs unvaccinated individuals, estimated from an unadjusted Cox proportional hazards model with the Efron method for ties.^58^ We used 2-sided Wald 95% CIs and 2-sided *P* values from score tests to assess whether vaccine efficacy departed from 0%; *P* < .05 indicated statistical significance.

## Results

**Figure 1.**
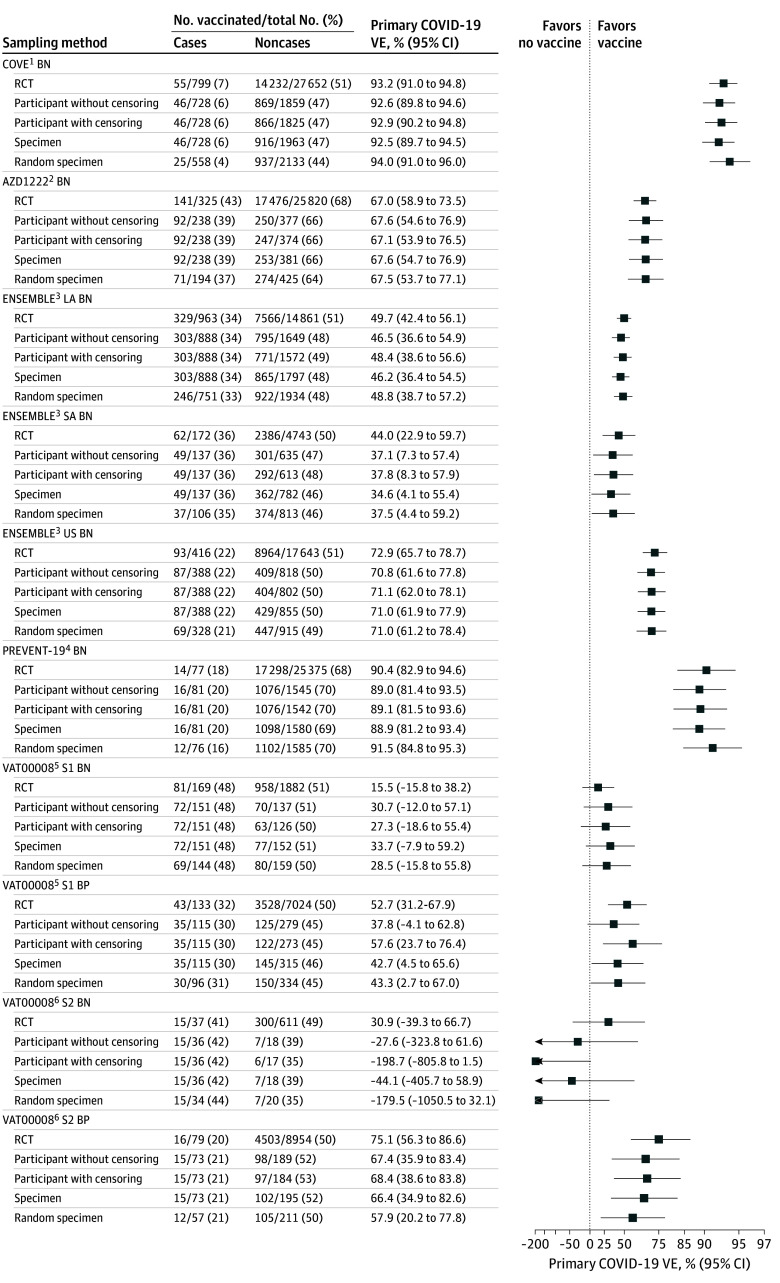
Primary COVID-19 Vaccine Efficacy and Semiparametric Logistic Regression Vaccine Effectiveness Estimates by Sampling Method Vaccine efficacy was estimated from 10 phase 3 COVID-19 Prevention Network randomized clinical trial (RCT) cohorts using the primary statistical approach in the final blinded phase analysis publications (Methods section and eTable 1 in Supplement 1).^1,2,3,4,5,6^ Test-negative design (TND) study datasets were constructed from the RCT cohorts using 4 TND sampling methods (Methods section). Vaccine effectiveness was defined as 1 minus the COVID-19 conditional risk ratio (vaccine vs placebo) and estimated on the TND study datasets using targeted maximum likelihood estimation under a semiparametric logistic regression model that adjusts for age, sex, race and ethnicity, region, comorbidities, and testing date (Methods section and eTable 2 in Supplement 1).^48^ Estimates and 95% CIs are compared on the natural logarithm (1 minus vaccine efficacy or effectiveness) scale, with plotting labels on the vaccine efficacy or effectiveness scale. BN indicates baseline SARS-CoV-2 negative; BP, baseline SARS-CoV-2 positive; LA, Latin America; S1, stage 1; S2, stage 2; and SA, South Africa.

In total, 12 157 participants were analyzed in the TND study datasets (eFigures 4 and 5 in Supplement 1), with a mean (SD) age of 45 (15) years. Of these, 6414 participants were female (53%) and 5743 were male (47%). A total of 5858 participants were vaccinated (48%), 2835 experienced primary COVID-19 (23%), and 2992 experienced CDC-defined COVID-19 (25%). Of 3181 primary and 3314 CDC-defined COVID-19 cases identified in the RCTs, 2788 (88%) and 2833 (85%), respectively, were accurately classified in TND analyses. Remaining RCT cases were excluded or misclassified as TND noncases due to ineligible positive SARS-CoV-2 test results. Differences in the number of noncases across both participant-based samples and the specimen-based sample reflect participants with multiple illness episodes (Figure 1 and eFigures 6-8 in Supplement 1). Differences in specimen-based and random specimen-based samples’ case numbers indicate case status misclassification rates from 5% to 28%, with the highest misclassification rates in COVE BN and VAT00008 stage 2 BP.

**Figure 2.**
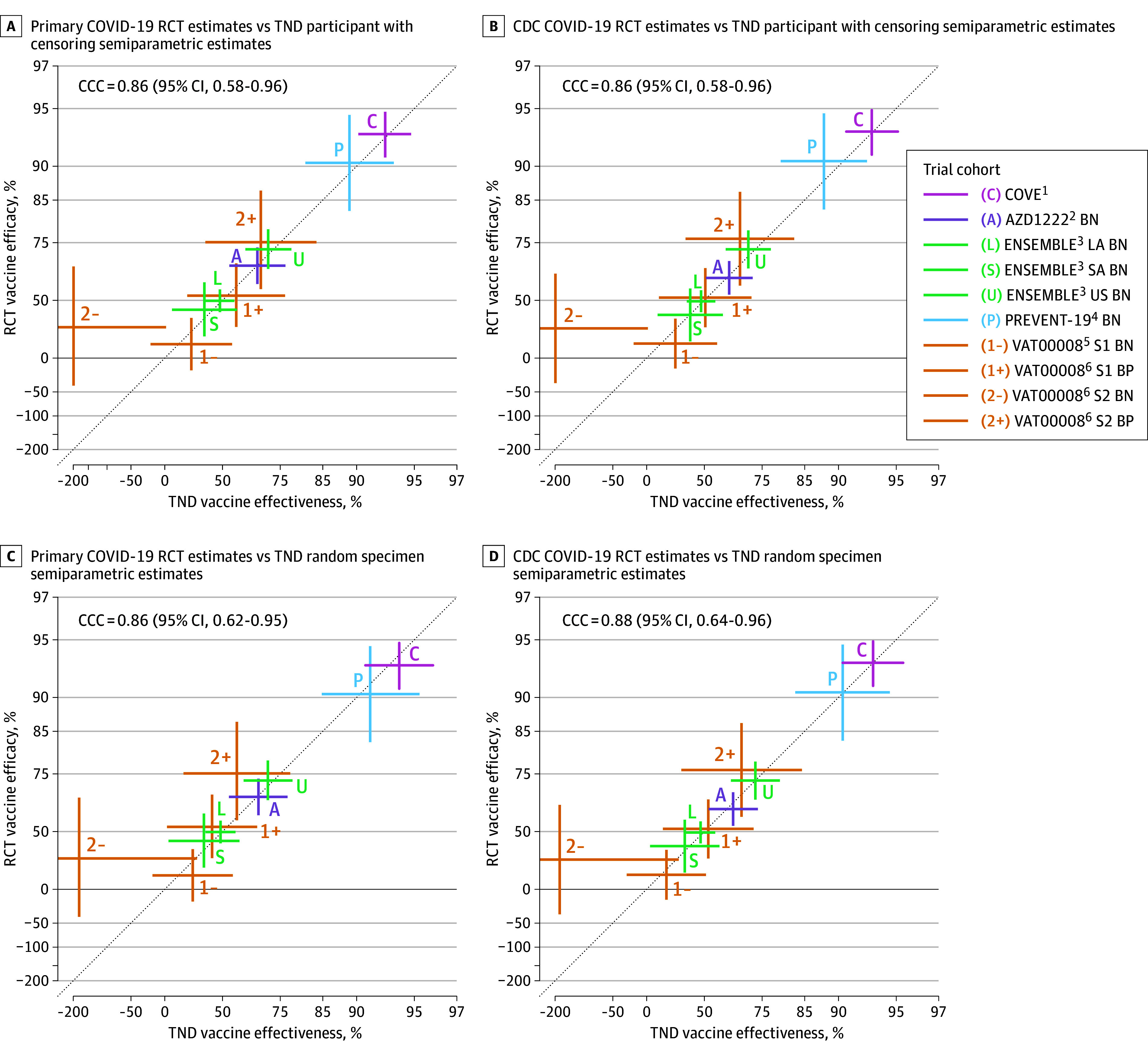
Placebo-Controlled Randomized Clinical Trial (RCT) Vaccine Efficacy Estimates vs Test-Negative Design (TND) Semiparametric Logistic Regression Vaccine Effectiveness Estimates A, Primary COVID-19 vaccine efficacy estimates from RCT cohorts and semiparametric logistic regression vaccine effectiveness estimates from primary COVID-19 TND participant-based samples with censoring for COVID-19. B, Centers for Disease Control and Prevention (CDC) COVID-19 vaccine efficacy estimates from RCT cohorts and semiparametric logistic regression vaccine effectiveness estimates from CDC COVID-19 TND participant-based samples with censoring for COVID-19. C, Primary COVID-19 vaccine efficacy estimates from RCT cohorts and semiparametric logistic regression vaccine effectiveness estimates from primary COVID-19 TND random specimen-based samples. D, CDC COVID-19 vaccine efficacy estimates from RCT cohorts and semiparametric logistic regression vaccine effectiveness estimates from CDC COVID-19 TND random specimen-based samples. Vaccine efficacy and effectiveness were estimated from 10 final blinded phase, primary efficacy analysis cohorts from 5 phase 3 COVID-19 Prevention Network RCTs.^1,2,3,4,5,6^ Vaccine efficacy was estimated using each trial’s primary efficacy analysis approach for primary COVID-19^1,2,3,4,5,6^ and an unadjusted Cox proportional hazards model for CDC COVID-19. Vaccine effectiveness was estimated using targeted maximum likelihood estimation under a semiparametric logistic regression model that adjusted for age, sex, race and ethnicity, region, comorbidities, and testing date.^48^ Estimates (symbols) and 95% CIs (vertical and horizontal line segments) are compared on the natural logarithm (1 minus vaccine efficacy or effectiveness) scale, with plotting labels on the vaccine efficacy or effectiveness scale. The 95% CI lower bounds for VAT00008 Stage 2 baseline SARS-CoV-2 negative (BN) TND estimates extend beyond the plotting region. Concordance correlation coefficient (CCC) estimates and 95% CIs are reported.^61^ BP indicates baseline SARS-CoV-2 positive; COVE Coronavirus Vaccine Efficacy and Safety; LA, Latin America; PREVENT-19, Prefusion Protein Subunit Vaccine Efficacy Novavax Trial COVID-19; S1, stage 1; S2, stage 2; and SA, South Africa.

The TND and RCT estimates were highly concordant for most trial cohorts and all symptom definitions, sampling methods, and statistical approaches, with concordance correlation coefficient estimates ranging from 0.85 to 0.95 (Figure 1 and Figure 2 and eTable 3 and eFigures 6-9 in Supplement 1). COVE BN primary COVID-19 TND estimates ranged from 92.5% to 94.0% compared with the RCT estimate of 93.2%; ENSEMBLE US BN primary COVID-19 TND estimates ranged from 70.8% to 71.1% compared with the RCT estimate of 72.9%; and VAT00008 stage 2 BP primary COVID-19 TND estimates ranged from 57.9% to 68.4% compared with the RCT estimate of 75.1%. TND variances were approximately 2 to 3 times larger than RCT variances (eTable 4 and eFigure 10 in Supplement 1), and all TND and RCT CIs overlapped. VAT00008 stage 2 BN TND estimates were negative with wide CIs that overlapped with RCT CIs. The semiparametric logistic regression and ordinary logistic regression estimates had similar bias, but the semiparametric regression had 29% to 48% smaller variance, depending on TND sampling method and symptom definition (eTable 4 and eFigure 10 in Supplement 1).

**Figure 3.**
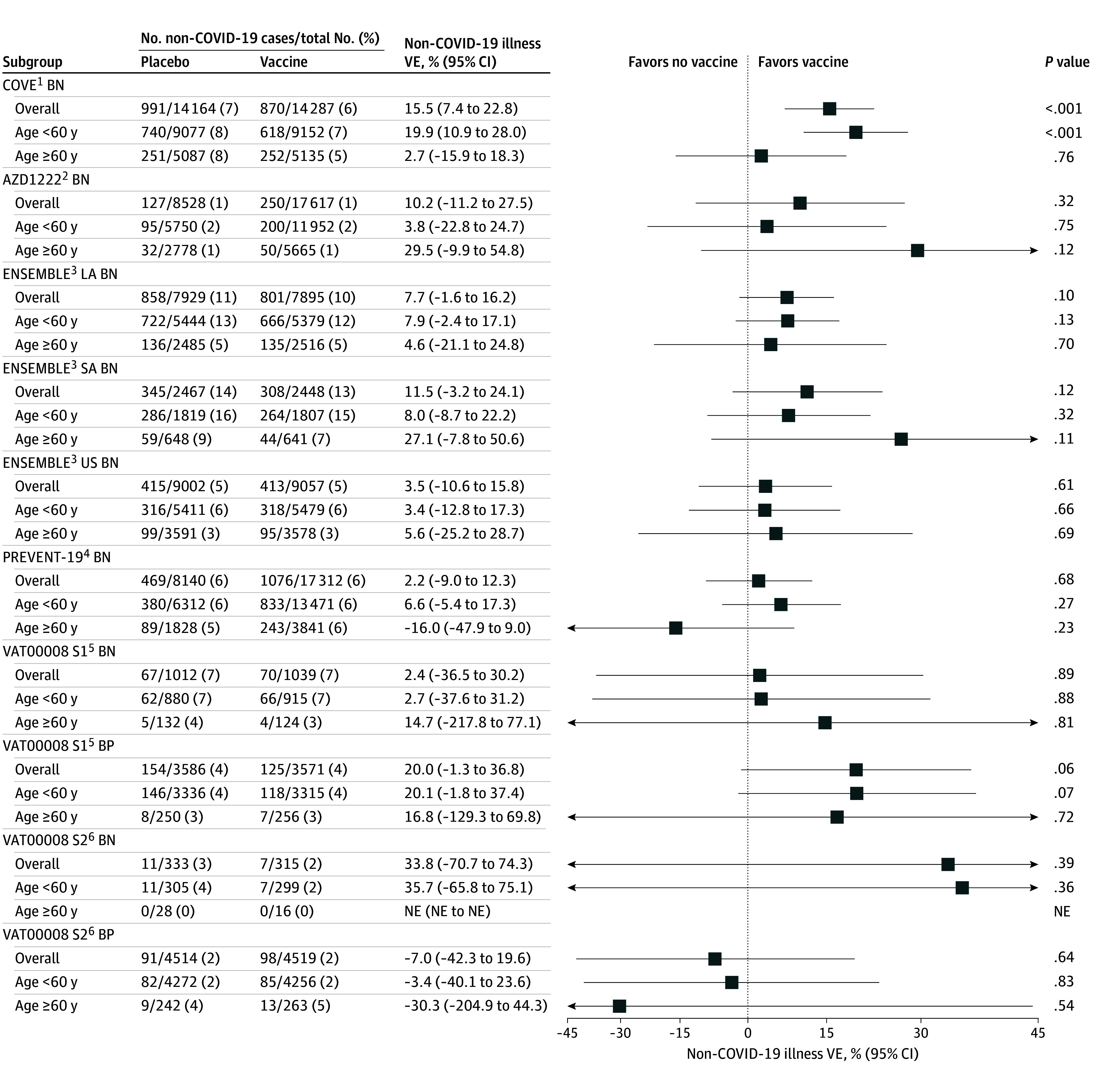
Vaccine Efficacy (VE) Against Non–COVID-19 Illness to Assess Noncase Exchangeability Violations VE against non–COVID-19 illness was defined as 1 minus the hazard ratio (vaccine vs placebo) of non–COVID-19 illness, estimated from an unadjusted Cox proportional hazards model using the Efron method for handling ties and score test *P* values. Models were fit on the overall randomized clinical trial (RCT) cohort and on subgroups younger than 60 years and 60 years or older for each final blinded phase of the phase 3 RCT primary efficacy analysis cohorts: COVE (Coronavirus Vaccine Efficacy and Safety) baseline SARS-CoV-2 negative (BN), AZD1222 BN, ENSEMBLE BN (analyzed separately as Latin America [LA], South Africa [SA], and the US), PREVENT-19 (Prefusion Protein Subunit Vaccine Efficacy Novavax Trial COVID-19) BN, and VAT00008 (analyzed separately by stage 1 [S1] monovalent and stage 2 [S2] bivalent vaccine BN and BP). Estimates and 95% CIs are compared on the natural logarithm (1 minus VE) scale, with plotting labels on the VE scale. Vaccine efficacy was not estimated in subgroups with no non–COVID-19 illness cases. BP indicates baseline SARS-CoV-2 positive; NE, not estimated.

When evaluating noncase exchangeability, all trials’ vaccine efficacy estimates against non–COVID-19 illness were near zero except in COVE BN (Figure 3). The COVE vaccine reduced non–COVID-19 illness overall (vaccine efficacy, 15.5%; 95% CI, 7.4%-22.8%; *P* < .001) and in individuals younger than 60 years (vaccine efficacy, 19.9%; 95% CI, 10.9%-28.0%; *P* < .001). In uniform quantile-quantile plots, the age-subgroup *P* values approximately follow a uniform distribution, but the overall cohort *P* value distribution deviates slightly from the identity line, suggesting some concern for noncase exchangeability (eFigure 11 in Supplement 1).

## Discussion

We evaluated the TND for estimation and inference on virologically confirmed symptomatic COVID-19 vaccine effectiveness in a health care–seeking population using 10 phase 3 trial cohorts with variable vaccine efficacies, COVID-19 incidences, SARS-CoV-2 variants, and demographic characteristics. We found high concordance between TND and RCT estimates. We also introduced a robust machine-learning approach that provides more flexible covariate adjustment, similar accuracy, and greater precision compared with ordinary logistic regression.

By analyzing TND study datasets from RCTs, we identified when the TND can reliably assess COVID-19 vaccine effectiveness in a health care–seeking population when confounding and selection bias are controlled. All TND sampling methods gave similar results to the RCTs; thus, the simple participant-based sampling method with censoring may be advantageous. The random specimen-based method illustrated that case status misclassification is present in postmarketing TND studies. NAAT accuracy may vary by time since exposure,^41^ type and manner of specimen collection,^62,63^ SARS-CoV-2 variants,^64^ and viral load^65^; regardless, resulting biases were negligible using these diagnostic tests.^13,41^ Both statistical approaches had more biased and variable TND estimates in the smaller TND study datasets with imbalances in vaccination status.^5,6^ Bias from the semiparametric logistic regression approach may be reduced by tailoring the approach for small samples (eg, using log-likelihood loss with leave-one-out cross-validation and machine-learning models with few input variables). When adjusting for many covariates in a small sample, ordinary logistic regression estimates can be severely biased and highly variable.^44,45,46^ Consequently, TND studies evaluating vaccines with presumably low efficacy should tailor the semiparametric approach for small samples or recruit more participants to avoid publishing negative vaccine effectiveness estimates with wide CIs and limit misinterpretation.

We also evaluated the effects of several COVID-19 vaccines in non–COVID-19 illness to detect noncase exchangeability violations. Only COVE BN demonstrated low-level statistically significant COVID-19 vaccine efficacy against non–COVID-19 illness. This vaccine may protect against some non–COVID-19 illnesses, which would lower TND vaccine effectiveness estimates. Alternatively, non–COVID-19 illness from false-negative SARS-CoV-2 test results could artificially cause the positive vaccine efficacy. Because the COVE vaccine reduces viral load^65^ and protects against COVID-19,^1^ vaccinated participants may be misclassified more than unvaccinated participants. This was observed in the COVE BN random specimen-based samples and increased vaccine effectiveness estimates.^66^ Unrecognized unblinding caused by high vaccine reactogenicity compared with placebo^1^ could also explain this statistical finding if it affected participants’ mask wearing, hygiene, and/or health care–seeking behavior. Despite the statistically significant result, COVE BN TND estimates were still close to RCT estimates. Future studies could evaluate vaccine effectiveness for non–COVID-19 illness in additional covariate subgroups adjusted for in TND analyses. Thus, TND analyses can assume noncase exchangeability for unbiased estimation, given that they also adjust for confounders of COVID-19 vaccination and non–COVID-19 illness, such as influenza vaccination status.^23,36^

Our study also illustrates the efficiency of the TND and semiparametric logistic regression approach. TND study datasets were less than one-quarter the size of their respective RCT cohorts, yet retained most RCT cases, with variance estimates only 2 to 3 times larger. Additionally, the semiparametric logistic regression approach produced smaller variance estimates than ordinary logistic regression because they were derived using the efficient influence function, which provides the smallest possible variance among regular asymptotically linear estimators using semiparametric efficiency theory.^48,67,68,69^ With smaller variance estimates, postmarketing studies can enroll fewer participants.

### Limitations

Our study has several limitations. First, postmarketing TND studies typically enroll a broader population than the CoVPN RCTs, which underrepresented or excluded some subpopulations such as children, immunocompromised groups, and individuals otherwise unlikely to receive vaccines.^1,2,3,4,5,6^ Additionally, we only evaluated TND studies in individuals who have identical health care–seeking behavior and always seek SARS-CoV-2 testing when experiencing COVID-19–like symptoms. This behavior is reasonable for individuals with severe COVID-19–like symptoms,^15,16,17,21^ but future studies should investigate more representative testing behavior for nonsevere symptoms that varies by vaccination status and covariates associated with COVID-19 diagnosis and potentially modifies vaccine effectiveness. To reduce selection bias, our semiparametric approach limits inference to a health care–seeking population, which may be difficult to define; however, recent causal TND methods leverage negative control variables to approximate health care–seeking behavior^70^ or stratify by reasons for testing^71^ to account for selection bias and generalize to the entire population. Moreover, our TND study datasets have larger ratios of cases to noncases than postmarketing TND studies due to fewer non–SARS-CoV-2 pathogens circulating early in the pandemic.^72^ Last, we evaluated the TND in an ideal setting that lacks the confounding, missing data, and misclassified data prevalent in many postmarketing TND studies. We applied our semiparametric logistic regression approach to illustrate covariate adjustment in a TND analysis but should conduct simulations with complex confounding and missing-at-random vaccination status to formally assess this method’s advantages. While we investigated case status misclassification from imperfect diagnostic tests, we did not assess vaccination status misclassification, which can arise from inaccurate self-reports and vaccine records.^31,40,73,74^ Misclassification of vaccination status and case status can be complex and induce bias in either direction in observational studies.^16,33,40,66,75,76^

## Conclusions

Since the COVID-19 pandemic, numerous TND studies have been conducted to evaluate COVID-19 vaccine effectiveness. Our analysis found that the TND can be applied to various COVID-19 settings and provide interpretable estimates, assuming other sources of bias are addressed. Future studies should evaluate TND performance for changes in health care–seeking behavior, time since vaccination, immunological biomarkers, and SARS-CoV-2 variants to ensure valid interpretations.

## Notes

Supplement 1. **eTable 1.** COVID-19 Prevention Network Phase 3 Randomized Placebo-Controlled Trial Characteristics **eTable 2.** Test-Negative Design Vaccine Effectiveness Covariate Adjustments for Semiparametric and Ordinary Logistic Regression **eTable 3.** Concordance Correlation Coefficients Between Randomized Placebo-Controlled Trial Vaccine Efficacy and Test-Negative Design Vaccine Effectiveness Estimates **eTable 4.** Semiparametric Logistic Regression and Ordinary Logistic Regression Bias, Relative Efficiency, and Mean Squared Error **eFigure 1.** Directed Acyclic Graph of Causal Relationships in a Test-Negative Design Study **eFigure 2.** Randomized Placebo-Controlled Trial vs Test-Negative Design for a Given COVID-19 End Point **eFigure 3.** Test-Negative Design Sampling Methods **eFigure 4.** Derivation of Primary COVID-19 End Point Test-Negative Design Participant-Based Samples With Censoring for COVID-19 **eFigure 5.** Derivation of CDC COVID-19 End Point Test-Negative Design Participant-Based Samples With Censoring for COVID-19 **eFigure 6.** CDC COVID-19 Vaccine Efficacy and Semiparametric Logistic Regression Vaccine Effectiveness Estimates by Sampling Method **eFigure 7.** Primary COVID-19 Vaccine Efficacy and Ordinary Logistic Regression Vaccine Effectiveness Estimates by Sampling Method **eFigure 8.** CDC COVID-19 Vaccine Efficacy and Ordinary Logistic Regression Vaccine Effectiveness Estimates by Sampling Method. **eFigure 9.** Randomized Placebo-Controlled Trial Vaccine Efficacy Estimates vs Test-Negative Design Ordinary Logistic Regression Vaccine Effectiveness Estimates **eFigure 10.** Semiparametric Logistic Regression and Ordinary Logistic Regression Bias and Variance Across All Trial Cohorts **eFigure 11.** Uniform Quantile-Quantile Plots of *P* Values Overall and by Age Subgroups to Assess Noncase Exchangeability Violations **eMethods.** Statistical Analysis
eReferences

Supplement 2. **Nonauthor Collaborators.** The COVID-19 Prevention Network (CoVPN)

Supplement 3.
Data Sharing Statement
