# Supplementary material for: Evaluating the Test-Negative Design for COVID-19 Vaccine Effectiveness Using Randomized Trial Data: A Secondary Cross-Protocol Analysis of 5 Randomized Clinical Trials
Source: JAMA Netw Open. 2025 May 28;8(5):e2512763. doi: 10.1001/jamanetworkopen.2025.12763 (PMC12120655; doi:10.1001/jamanetworkopen.2025.12763)
Supplement: Supplement 1. — eTable 1. COVID-19 Prevention Network Phase 3 Randomized Placebo-Controlled Trial Characteristics eTable 2. Test-Negative Design Vaccine Effectiveness Covariate Adjustments for Semiparametric and Ordinary Logistic Regression eTable 3. Concordance Correlation Coefficients Between Randomized Placebo-Controlled Trial Vaccine Efficacy and Test-Negative Design Vaccine Effectiveness Estimates eTable 4. Semiparametric Logistic Regression and Ordinary Logistic Regression Bias, Relative Efficiency, and Mean Squared Error eFigure 1. Directed Acyclic Graph of Causal Relationships in a Test-Negative Design Study eFigure 2. Randomized Placebo-Controlled Trial vs Test-Negative Design for a Given COVID-19 End Point eFigure 3. Test-Negative Design Sampling Methods eFigure 4. Derivation of Primary COVID-19 End Point Test-Negative Design Participant-Based Samples With Censoring for COVID-19 eFigure 5. Derivation of CDC COVID-19 End Point Test-Negative Design Participant-Based Samples With Censoring for COVID-19 eFigure 6. CDC COVID-19 Vaccine Efficacy and Semiparametric Logistic Regression Vaccine Effectiveness Estimates by Sampling Method eFigure 7. Primary COVID-19 Vaccine Efficacy and Ordinary Logistic Regression Vaccine Effectiveness Estimates by Sampling Method eFigure 8. CDC COVID-19 Vaccine Efficacy and Ordinary Logistic Regression Vaccine Effectiveness Estimates by Sampling Method. eFigure 9. Randomized Placebo-Controlled Trial Vaccine Efficacy Estimates vs Test-Negative Design Ordinary Logistic Regression Vaccine Effectiveness Estimates eFigure 10. Semiparametric Logistic Regression and Ordinary Logistic Regression Bias and Variance Across All Trial Cohorts eFigure 11. Uniform Quantile-Quantile Plots of P Values Overall and by Age Subgroups to Assess Noncase Exchangeability Violations eMethods. Statistical Analysis eReferences [file jamanetwopen-e2512763-s001.pdf]

Supplemental Online Content

Andrews LIB, Halloran ME, Neuzil KM, et al; COVID-19 Prevention Network (CoVPN). Evaluating the test-negative design for COVID-19 vaccine effectiveness using randomized trial data: a secondary cross-protocol analysis of 5 randomized trials. *JAMA Network Open*. 2025;8(5):e2512763. doi:10.1001/jamanetworkopen.2025.12763

eTable 1. COVID-19 Prevention Network Phase 3 Randomized Placebo-Controlled Trial Characteristics

eTable 2. Test-Negative Design Vaccine Effectiveness Covariate Adjustments for Semiparametric and Ordinary Logistic Regression

eTable 3. Concordance Correlation Coefficients Between Randomized Placebo-Controlled Trial Vaccine Efficacy and Test-Negative Design Vaccine Effectiveness Estimates

eTable 4. Semiparametric Logistic Regression and Ordinary Logistic Regression Bias, Relative Efficiency, and Mean Squared Error

eFigure 1. Directed Acyclic Graph of Causal Relationships in a Test-Negative Design Study

eFigure 2. Randomized Placebo-Controlled Trial vs Test-Negative Design for a Given COVID-19 End Point

eFigure 3. Test-Negative Design Sampling Methods

eFigure 4. Derivation of Primary COVID-19 End Point Test-Negative Design Participant-Based Samples With Censoring for COVID-19

eFigure 5. Derivation of CDC COVID-19 End Point Test-Negative Design Participant-Based Samples With Censoring for COVID-19

eFigure 6. CDC COVID-19 Vaccine Efficacy and Semiparametric Logistic Regression Vaccine Effectiveness Estimates by Sampling Method

eFigure 7. Primary COVID-19 Vaccine Efficacy and Ordinary Logistic Regression Vaccine Effectiveness Estimates by Sampling Method

eFigure 8. CDC COVID-19 Vaccine Efficacy and Ordinary Logistic Regression Vaccine Effectiveness Estimates by Sampling Method.

eFigure 9. Randomized Placebo-Controlled Trial Vaccine Efficacy Estimates vs Test-Negative Design Ordinary Logistic Regression Vaccine Effectiveness Estimates

eFigure 10. Semiparametric Logistic Regression and Ordinary Logistic Regression Bias and Variance Across All Trial Cohorts

eFigure 11. Uniform Quantile-Quantile Plots of *P* Values Overall and by Age Subgroups to Assess Noncase Exchangeability Violations

© 2025 Andrews LIB et al. *JAMA Network Open*.

eMethods. Statistical Analysis

eReferences

This supplementary material has been provided by the authors to give readers additional information about their work.

© 2025 Andrews LIB et al. *JAMA Network Open*.

eTable 1. COVID-19 Prevention Network Phase 3 Randomized Placebo-Controlled Trial Characteristics[1]

Characteristic	Moderna COVE[2]	AstraZeneca/ Oxford AZD1222[3]	Janssen ENSEMBLE[4]	Novavax PREVENT-19[5]	Sanofi/GSK VAT00008 Stage 1[6]	Sanofi/GSK VAT00008 Stage 2[7]
Location	United States	Chile, Peru, United States	Argentina, Brazil, Chile, Colombia, Mexico, Peru, South Africa, United States	Mexico, United States	Columbia, Ghana, Honduras, India, Japan, Kenya, Nepal, United States	Colombia, Ghana, India, Kenya, Mexico, Nepal, Uganda, Ukraine
Final Blinded Phase, Primary Efficacy Analysis Cohort	Per-protocol cohort (baseline SARS-CoV-2 negative and received two doses of assigned intervention)	Fully vaccinated analysis set (baseline SARS-CoV-2 negative and received two doses of intervention) and were retained on study for 15 days after dose 2	Per-protocol at-risk cohort (baseline SARS-CoV-2 negative, received assigned intervention, and did not have SARS-CoV-2 positive test before 15 days after dose)	Per-protocol efficacy cohort (baseline SARS-CoV-2 negative and received two doses of assigned intervention) and did not have a censoring event at any time before 7 days after the second dose	Modified full analysis set post-dose 2 (received two doses of intervention) and did not discontinue within 14 days of the second dose, excluding participants missing baseline SARS-CoV-2 status	
Baseline SARS-CoV-2 Negative Definition (Baseline SARS-CoV-2 Positive = Complement)	SARS-CoV-2 negative PCR test and SARS-CoV-2 nucleocapsid binding antibody levels below the LLOQ on day 1	SARS-CoV-2 nucleocapsid binding antibody levels below the LLOQ on day 1	SARS-CoV-2 negative PCR test and SARS-CoV-2 nucleocapsid binding antibody levels below the positivity cut-off on day 1 (missing values also classified as baseline SARS-CoV-2 negative)	SARS-CoV-2 negative PCR test and nucleocapsid binding antibody levels below the LLOQ on day 0	No detection of SARS-CoV-2 nucleic acids via NAAT (day 1 and day 22), no detection of anti-nucleocapsid antibodies (day 1 and day 22), and no detection of anti-spike antibodies (day 1)	
Primary Efficacy COVID-19 Endpoint	Pneumonia, shortness of breath/difficulty breathing, or	Pneumonia, oxygen saturation ≤ 94% or require supplemental oxygen, or	Pneumonia, respiratory symptoms, deep vein thrombosis,	Pneumonia, evidence of significant lower respiratory tract infection, abnormal lung	Pneumonia, shortness of breath, altered level of consciousness, myocarditis, thromboembolic event, purpura fulminans, fever, chilblains (≥ 1 symptom); OR	

© 2025 Andrews LIB et al. *JAMA Network Open*.

Characteristic	Moderna COVE[2]	AstraZeneca/ Oxford AZD1222[3]	Janssen ENSEMBLE[4]	Novavax PREVENT-19[5]	Sanofi/GSK VAT00008 Stage 1[6]	Sanofi/GSK VAT00008 Stage 2[7]
	cough (≥1 symptom); OR fever, chills, myalgia, headache, sore throat, or loss of taste or smell (≥2 symptoms) plus PCR confirmation	dyspnea/shortness of breath (≥ 1 symptom); OR fever/feverishness, cough, myalgia/muscle pain, fatigue, vomiting/diarrhea, or loss of taste or smell anosmia (≥ 2 symptoms) plus PCR confirmation	severe systemic illness, respiratory rate >20 beats/minute, oxygen saturation ≤ 94%, shock, (≥ 1 symptom); OR fever or chills, cough, elevated heart rate, muscle or body pain, headache, red or bruised-looking feet or toes, gastrointestinal symptoms, loss of taste or smell (≥ 2 symptoms) plus PCR confirmation	auscultation, fever, cough, elevated heart rate, abnormal oxygen saturation, organ dysfunction, ICU admission, or death (≥1 symptom); OR shortness of breath, fatigue, body aches, headache, loss of taste or smell; sore throat/congestion/runny nose, or nausea/vomiting/diarrhea (≥2 symptoms) plus PCR confirmation	cough, loss of smell, loss of taste (≥ 1 symptom, ≥ 24 hours); OR sore throat, chills, myalgia, fatigue, malaise, headache, nasal congestion/rhinorrhea, abdominal pain, or vomiting/diarrhea/nausea (≥ 2 symptoms, ≥ 24 hours) plus NAAT confirmation	
CDC COVID-19 Endpoint	Fever/chills, cough, shortness of breath/difficulty breathing, fatigue, muscle aches/body aches, headache, loss of taste or smell, sore throat, nasal congestion/rhinorrhea, nausea/vomiting, or diarrhea (≥ 1 symptom) plus PCR or NAAT confirmation					
RCT Statistical Analysis	VE = 1 – HR (vaccine vs. placebo), estimated using a Cox proportional hazards model stratified by randomization factors (≥18 to <65 years and not at risk, ≥18 to <65 years and at risk, and	VE = 1 – RR (vaccine vs. placebo), estimated using a Poisson regression model and robust standard errors, adjusting for study arm, age group (<65 and ≥65 years), and follow-up time[9]	VE = 1– ratio of incidence rates of vaccine vs. placebo, which was estimated using exact Poisson regression[10]	VE = 1 – RR (vaccine vs. placebo), estimated using a Poisson regression model and robust standard errors,[9] adjusting for study arm and age group (< 65 and ≥ 65 years)	VE = 1 – ratio of incidence rates per 1000 person-years of vaccine vs. placebo, with confidence intervals calculated by the exact method,[11] assuming vaccinated cases follow a binomial distribution after conditioning on total cases	

© 2025 Andrews LIB et al. *JAMA Network Open*.

Characteristic	Moderna COVE[2]	AstraZeneca/ Oxford AZD1222[3]	Janssen ENSEMBLE[4]	Novavax PREVENT-19[5]	Sanofi/GSK VAT00008 Stage 1[6]	Sanofi/GSK VAT00008 Stage 2[7]
	≥65 years) and Efron's method for handling ties[8]					

Abbreviations: COVID-19 = Coronavirus Disease of 2019; SARS-CoV-2 = Severe Acute Respiratory Syndrome Coronavirus 2; PCR = Polymerase Chain Reaction; NAAT = Nucleic Acid Amplification Test; CDC = Centers for Disease Control and Prevention; RCT = Randomized Placebo-Controlled Trial; ICU = Intensive Care Unit; LLOQ = Lower Limit of Quantification; VE = Vaccine Efficacy; HR = Hazard Ratio; RR = Relative Risk

© 2025 Andrews LIB et al. *JAMA Network Open*.

eTable 2. Test-Negative Design Vaccine Effectiveness Covariate Adjustments for Semiparametric and Ordinary Logistic Regression

Trial Cohort	Age	Sex	Race/Ethnicity	Geographic Region	Comorbidities	SARS-CoV-2 Testing Date
**Moderna COVE BN[2]**	Continuous, in years	Male and female	People of Color and Non-Hispanic/Latino White. Participants who could not be classified were considered missing and excluded from TND analyses[a]	Northeast, Midwest, South, and West, using United States Census Groupings[12]	At least one risk factor for severe COVID-19 (chronic lung disease, significant cardiac disease, severe obesity, diabetes, liver disease, human immunodeficiency virus infection) and no risk factor for severe COVID-19	Two-week bins
**AstraZeneca/Oxford AZD1222 BN[3]**	Continuous, in years	Male and female	No adjustment, given limited racial/ethnic heterogeneity in some geographical regions	Chile, Peru, and United States	At least one COVID-19 comorbidity (chronic kidney disease, chronic obstructive pulmonary disease, lower immune health because of a solid organ transplant, history of obesity, serious heart conditions, sickle cell disease, type 2 diabetes, asthma, dementia, cerebrovascular disease, cystic fibrosis, high blood pressure, liver disease, scarring in the lungs/pulmonary fibrosis, type 1 diabetes, thalassemia, history of smoking) and no COVID-19 comorbidities	Two-week bins
**Janssen ENSEMBLE Latin America BN[4]**<br>**Janssen ENSEMBLE South Africa BN[4]**<br>**Janssen ENSEMBLE United States BN[4]**	Continuous, in years	Male and female	People of Color and Non-Hispanic/Latino White. Participants who could not be classified were considered missing and excluded from TND analyses[a]	No additional adjustment	At least one coexisting condition associated with severe COVID-19 (asthma, cancer, cerebrovascular disease, cystic fibrosis, chronic kidney disease, chronic obstructive pulmonary disease, serious heart condition, hypertension, immunocompromised state from blood transplant or organ transplant, liver disease, neurologic condition, obesity, pulmonary fibrosis, sickle cell disease, type 1 or type 2 diabetes, thalassemia) and no coexisting conditions associated with severe COVID-19	Two-week bins

© 2025 Andrews LIB et al. *JAMA Network Open*.

<table>
<tr><th>Trial Cohort</th><th>Age</th><th>Sex</th><th>Race/Ethnicity</th><th>Geographic Region</th><th>Comorbidities</th><th>SARS-CoV-2 Testing Date</th></tr>
<tr><td>Novavax PREVENT-19 BN[5]</td><td>Continuous, in years</td><td>Male and female</td><td>No adjustment, given limited racial/ethnic heterogeneity in some geographical regions</td><td>Mexico and United States</td><td>At least one coexisting condition associated with severe COVID-19 comorbidities (obesity, chronic kidney or lung disease, cardiovascular disease, diabetes mellitus type 2) and no coexisting conditions associated with severe COVID-19</td><td>Two-week bins</td></tr>
<tr><td>Sanofi/GSK VAT00008 Stage 1 BN[6]</td><td rowspan="4">Continuous, in years</td><td rowspan="4">Male and female</td><td rowspan="4">No adjustment, given limited racial/ethnic heterogeneity in some geographical regions</td><td rowspan="2">Africa (Ghana, Kenya), Asia (India, Japan, Nepal), Latin America (Columbia, Honduras), and United States</td><td rowspan="4">At least one high-risk medical condition associated with severe COVID-19 (cancer, chronic kidney disease, chronic obstructive pulmonary disease, obesity, heart conditions such as heart failure, coronary artery disease or cardiomyopathies, sickle cell disease, thalassemia, type 1 or type 2 diabetes mellitus, moderate-to-severe asthma, cerebrovascular disease, cystic fibrosis, hypertension/high blood pressure, neurologic conditions, hepatic disease, pulmonary fibrosis, smoking, immunocompromised state from solid organ transplant, blood or bone marrow transplant, immune deficiencies, human immunodeficiency virus, use of corticosteroids, or use of immunosuppressors) and no high-risk medical conditions associated with severe COVID-19</td><td rowspan="4">Two-week bins</td></tr>
<tr><td>Sanofi/GSK VAT00008 Stage 1 BP[6]</td></tr>
<tr><td>Sanofi/GSK VAT00008 Stage 2 BN[7]</td><td rowspan="2">Africa (Ghana, Kenya, Uganda), Asia (India, Nepal), and Latin America (Colombia, Mexico). As was done in the final blinded phase, primary efficacy analyses, Ukraine was omitted from TND analyses</td></tr>
<tr><td>Sanofi/GSK VAT00008 Stage 2 BP[7]</td></tr>
</table>

Age was adjusted for as a continuous variable and all other covariates were adjusted for as binary or categorical unordered covariates.

[a] People of Color was defined as participants who reported their race as American Indian or Alaska Native, Asian, Black or African American, Multiple, Native Hawaiian or Other Pacific Islander, or Other, and/or reported their ethnicity as Hispanic or Latino. Non-Hispanic/Latino White was defined as participants who reported their race as White and reported their ethnicity as Not Hispanic or Latino or did not report their ethnicity. Participants who did not report their race and either did not report their ethnicity or reported their ethnicity as Not Hispanic or Latino were classified as missing and excluded from TND analyses.

All covariates except date of testing were recorded at randomized placebo-controlled trial enrollment.

Abbreviations: BN = Baseline SARS-CoV-2 Negative; BP = Baseline SARS-CoV-2 Positive; SARS-CoV-2 = Severe Acute Respiratory Syndrome Coronavirus 2; TND = Test-Negative Design; COVID-19 = Coronavirus Disease 2019

© 2025 Andrews LIB et al. *JAMA Network Open*.

eTable 3. Concordance Correlation Coefficients Between Randomized Placebo-Controlled Trial Vaccine Efficacy and Test-Negative Design Vaccine Effectiveness Estimates

TND Sampling Method	Primary COVID-19		CDC COVID-19	
	**Semiparametric Logistic Regression** CCC (95% CI)	**Ordinary Logistic Regression** CCC (95% CI)	**Semiparametric Logistic Regression** CCC (95% CI)	**Ordinary Logistic Regression** CCC (95% CI)
Participant w/o Censoring	0.95 (0.84, 0.99)	0.93 (0.76, 0.98)	0.95 (0.83, 0.99)	0.93 (0.74, 0.98)
Participant w/ Censoring	0.86 (0.58, 0.96)	0.87 (0.61, 0.96)	0.86 (0.58, 0.96)	0.87 (0.59, 0.96)
Specimen	0.94 (0.79, 0.98)	0.93 (0.76, 0.98)	0.94 (0.79, 0.98)	0.93 (0.75, 0.98)
Random Specimen	0.86 (0.62, 0.95)	0.85 (0.60, 0.95)	0.88 (0.64, 0.96)	0.86 (0.59, 0.95)

The concordance correlation coefficient compared RCT vaccine efficacy estimates and TND vaccine effectiveness estimates from ten trial cohorts on the natural logarithm scale, ln(1-VE), for a given COVID-19 symptom definition, TND sampling method, and statistical method. 95% confidence intervals were computed via Z-transformation.[13]
Vaccine efficacy and effectiveness estimates were computed from ten phase 3 RCT final blinded phase, primary efficacy analysis cohorts: Moderna COVE BN, AstraZeneca/Oxford AZD1222 BN, Janssen ENSEMBLE BN (analyzed separately as Latin America, South Africa, and United States), Novavax PREVENT-19 BN, and Sanofi/GSK VAT00008 (analyzed separately by Stage 1 monovalent and Stage 2 bivalent vaccine and by baseline SARS-CoV-2 negative and positive status). RCT vaccine efficacy results were derived using trials' final blinded phase, primary efficacy analysis approaches for primary COVID-19 and unadjusted Cox proportional hazards models for CDC COVID-19.
TND vaccine effectiveness estimates derived from semiparametric logistic regression were estimated using targeted maximum likelihood estimation under a partially linear logistic regression model that flexibly adjusted for age, sex, race/ethnicity, region, comorbidities, and two-week testing date intervals and allowed for two-way covariate interactions. TND vaccine effectiveness estimates derived from the ordinary logistic regression adjusted for age, sex, race/ethnicity, region comorbidities, and two-week testing date linear main effects.
Abbreviations: TND = Test-Negative Design; COVID-19 = Coronavirus Disease 2019; CDC = Centers for Disease Control and Prevention; CCC = Concordance Correlation Coefficient; CI = Confidence Interval; w/o = without; w/ = with; VE = Vaccine Efficacy or Vaccine Effectiveness; ln = natural logarithm

© 2025 Andrews LIB et al. *JAMA Network Open*.

eTable 4. Semiparametric Logistic Regression and Ordinary Logistic Regression Bias, Relative Efficiency, and Mean Squared Error

**Case Definition**	**TND Sampling Method**	**Semiparametric Logistic Regression**			**Ordinary Logistic Regression**			**Semiparametric Logistic Regression vs. Ordinary Logistic Regression**
		Mean Bias[a]	Relative Efficiency to RCT[b]	Mean Squared Error[c]	Mean Bias[a]	Relative Efficiency to RCT[b]	Mean Squared Error[c]	Relative Efficiency[d]
Primary COVID-19	Participant w/o Censoring	0.14	1.87	0.10	0.11	2.85	0.14	0.66
	Participant w/ Censoring	0.18	1.79	0.11	0.17	3.37	0.18	0.53
	Specimen	0.15	1.93	0.11	0.14	2.80	0.14	0.69
	Random Specimen	0.19	2.29	0.14	0.18	3.23	0.17	0.71
CDC COVID-19	Participant w/o Censoring	0.11	1.97	0.09	0.12	2.94	0.14	0.67
	Participant w/ Censoring	0.19	1.84	0.11	0.17	3.50	0.17	0.52
	Specimen	0.14	2.02	0.10	0.14	2.89	0.14	0.70
	Random Specimen	0.16	2.47	0.13	0.17	3.46	0.17	0.71

[a] Mean bias was computed on the ln scale across all ten trial cohorts, using $\frac{1}{10}\sum_{i=1}^{10}\left[\ln\left(1-\widehat{VE}_{TND,i}\right)-\ln\left(1-\widehat{VE}_{RCT,i}\right)\right]$.

[b] Relative efficiency to RCT was computed using the ratio of the average variance of $\ln\left(1-\widehat{VE}\right)$ across all ten trial cohorts for a given TND sampling method and the RCT method. For simplicity, RCT variance was approximated assuming the published RCT $\widehat{VE}$ confidence intervals were Wald 95% confidence intervals.

[c] Mean squared error was computed using the sum of the average variance of $\ln\left(1-\widehat{VE}\right)$ across all ten trial cohorts and the mean bias across all ten trial cohorts squared.

[d] Relative efficiency was computed as the ratio between the average variance of $\ln\left(1-\widehat{VE}\right)$ across all ten trial cohorts for a given TND sampling method estimated using semiparametric logistic regression vs. ordinary logistic regression.

Abbreviations: COVID-19 = Coronavirus Disease 2019; CDC = Centers for Disease Control and Prevention; RCT = Randomized Placebo-Controlled Trial; TND = Test-Negative Design; w/o = without; w/ = with; VE = Vaccine Efficacy or Vaccine Effectiveness; $\widehat{VE}$ = Estimated VE; ln = natural logarithm

© 2025 Andrews LIB et al. *JAMA Network Open*.

eFigure 1. Directed Acyclic Graph of Causal Relationships in a Test-Negative Design Study

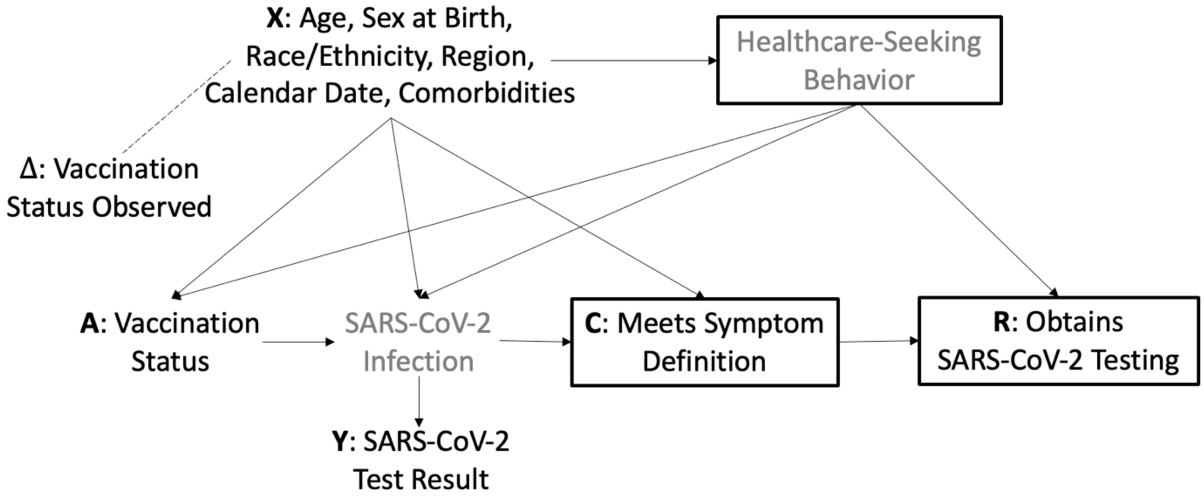

Directed acyclic graph illustrating possible relationships between variables in a test-negative design study. Solid black arrows indicate causal relationships and dashed lines indicate associations. Black variables are measured in a test-negative design while gray variables are unknown. The black boxes around C, R, and healthcare-seeking behavior (i.e., propensity to seek care when ill) reflect how the test-negative design restricts to individuals who meet the symptom definition and obtain SARS-CoV-2 testing, thereby assuming all TND individuals have the same healthcare-seeking behavior. Consequently, most TND analyses either generalize to a healthcare-seeking population only or assume no effect modification by healthcare-seeking behavior and generalize to the entire population.

© 2025 Andrews LIB et al. *JAMA Network Open*.

eFigure 2. Randomized Placebo-Controlled Trial vs Test-Negative Design for a Given COVID-19 End Point

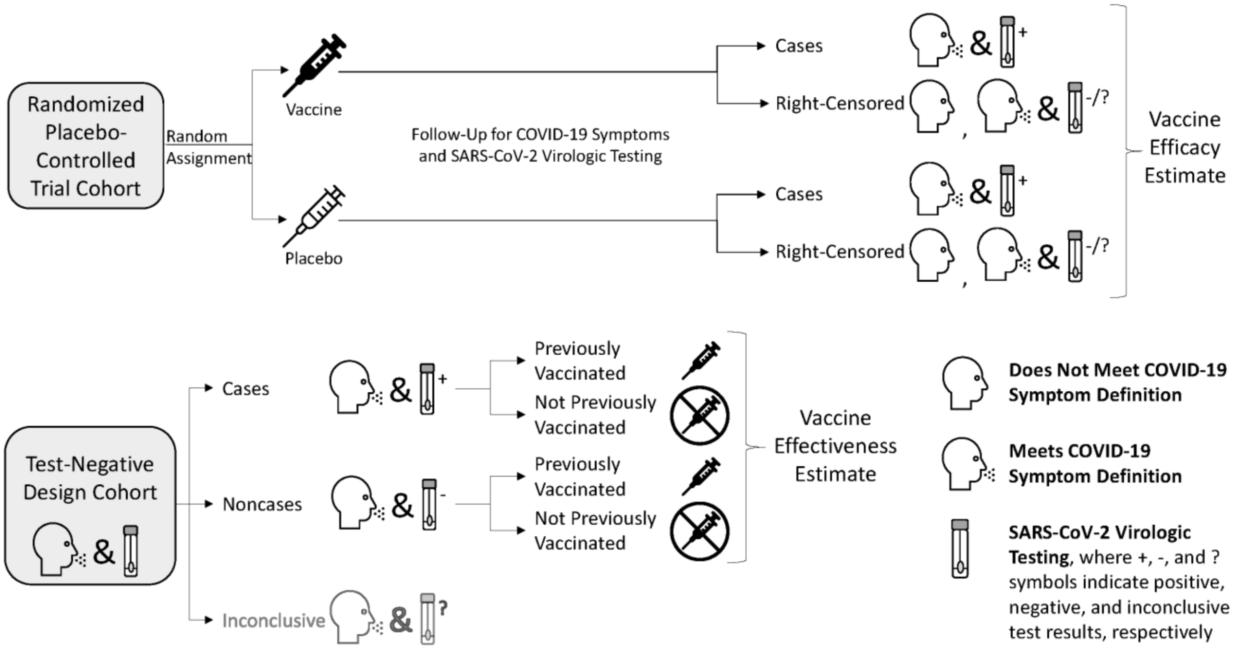

In an RCT, enrolled participants are randomly assigned to receive the vaccine or placebo and followed for months or years for occurrence of a given COVID-19 endpoint. Participants are RCT cases if they meet the COVID-19 symptom definition and obtain a positive SARS-CoV-2 viral RNA test and are right-censored at unblinding, receipt of non-study COVID-19 vaccination, loss to follow-up, or end of blinded phase if they never meet the symptom definition or meet the symptom definition but only obtain negative or inconclusive SARS-CoV-2 tests. Causal vaccine efficacy estimates can be obtained given randomization and blinding. In a TND, enrolled participants meet the COVID-19 symptom definition and are seeking SARS-CoV-2 testing. TND cases test SARS-CoV-2 positive and TND noncases test SARS-CoV-2 negative. Participants self-report vaccination status and additional covariates or release relevant medical records at testing. Causal vaccine effectiveness estimates are obtained under stronger assumptions than needed for an RCT analysis. If the same COVID-19 symptom definition and testing criteria are applied, participants who classify as cases in an RCT would also classify as cases in a TND.
Abbreviations: RCT = Randomized Placebo-Controlled Trial; COVID-19 = Coronavirus Disease of 2019; RNA = Ribonucleic Acid; SARS-CoV-2 = Severe Acute Respiratory Syndrome Coronavirus 2; TND = Test-Negative Design

© 2025 Andrews LIB et al. *JAMA Network Open*.

eFigure 3. Test-Negative Design Sampling Methods

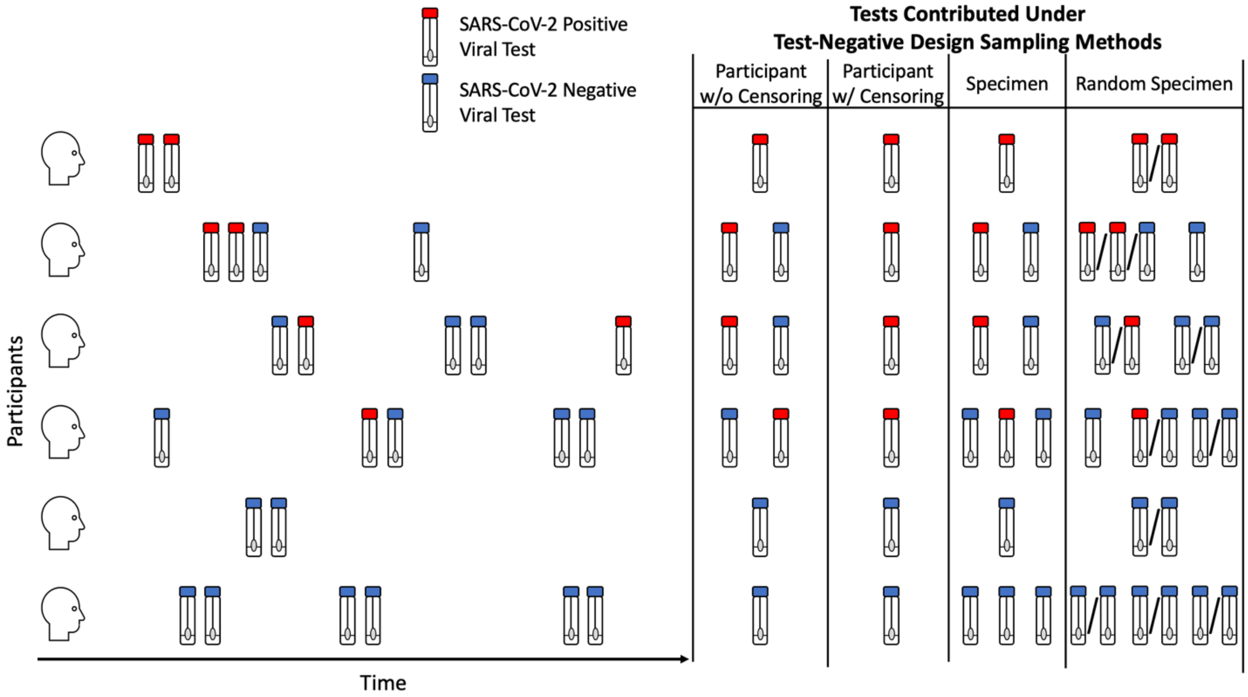

Four TND sampling methods applied to six RCT participants' eligible SARS-CoV-2 tests to form distinct TND study datasets. The SARS-CoV-2 tests in the figure occur at least one or two weeks after completing the intervention (depending on RCT protocol), within ten days of symptom onset, after meeting a given symptom definition, while blinded, and before receiving any non-study COVID-19 vaccinations. SARS-CoV-2 test groupings are from the same illness episode or symptomatic period and are at least 30 days apart. We assume illness episodes with at least one SARS-CoV-2 positive test are caused by SARS-CoV-2 (i.e., a positive episode) and illness episodes with only SARS-CoV-2 negative tests are not caused by SARS-CoV-2 (i.e., a negative episode). In the participant-based sample without censoring for COVID-19, cases are participants with at least one positive episode and contribute the first SARS-CoV-2 positive test from their first positive episode. For simplicity, we select the first SARS-CoV-2 positive test as all eligible SARS-CoV-2 positive tests from an illness episode are within ten days apart. We select the first positive episode because subsequent SARS-CoV-2 positive tests are likely from non-viable virus shedding from the first positive episode given the low probability of SARS-CoV-2 reinfection in 40 weeks.[14-17] Noncases are participants with at least one negative episode (including participants with positive episodes) and contribute the first SARS-CoV-2 negative test from their first negative episode. We select the first SARS-CoV-2 negative test and first negative episode for simplicity. Under this sampling method, each participant contributes one or two SARS-CoV-2 tests. In the participant-based sample with censoring for COVID-19, cases are defined identically and noncases are participants with no positive episodes and at least one negative episode. Noncases contribute their first SARS-CoV-2 negative test from their first negative episode. Under this sampling method, each participant contributes one SARS-CoV-2 test. In the specimen-based and random specimen-based samples, episodes rather than participants are the units of analysis. In the specimen-based sample, cases are participants' first positive episode and contribute the first SARS-CoV-2 positive test from that illness episode. We ignore subsequent positive episodes as before. Noncases are participants' negative episodes and contribute the first SARS-CoV-2 negative test from each negative episode. Under this sampling method, each participant contributes one SARS-CoV-2 positive test from their first positive episode (if they have one) and one SARS-CoV-2 negative test from every negative episode. By construction, the two participant-based samples and the specimen-based sample accurately classify case status and have the same number of cases. In the random specimen-based sample, participants contribute one randomly selected SARS-CoV-2 test per illness episode (denoted with "/" in the figure) to determine that illness episode's (potentially misclassified) case status. Participants' first positive episode and all negative episodes are considered, such that both specimen-based samples have the same number of illness episodes and SARS-CoV-2 tests to ease comparisons.

Abbreviations: SARS-CoV-2 = Severe Acute Respiratory Syndrome; w/ = with; w/o = without; TND = Test-Negative Design; RCT = Randomized Placebo-Controlled Trial; COVID-19 = Coronavirus Disease of 2019

© 2025 Andrews LIB et al. *JAMA Network Open*.

eFigure 4. Derivation of Primary COVID-19 End Point Test-Negative Design Participant-Based Samples With Censoring for COVID-19

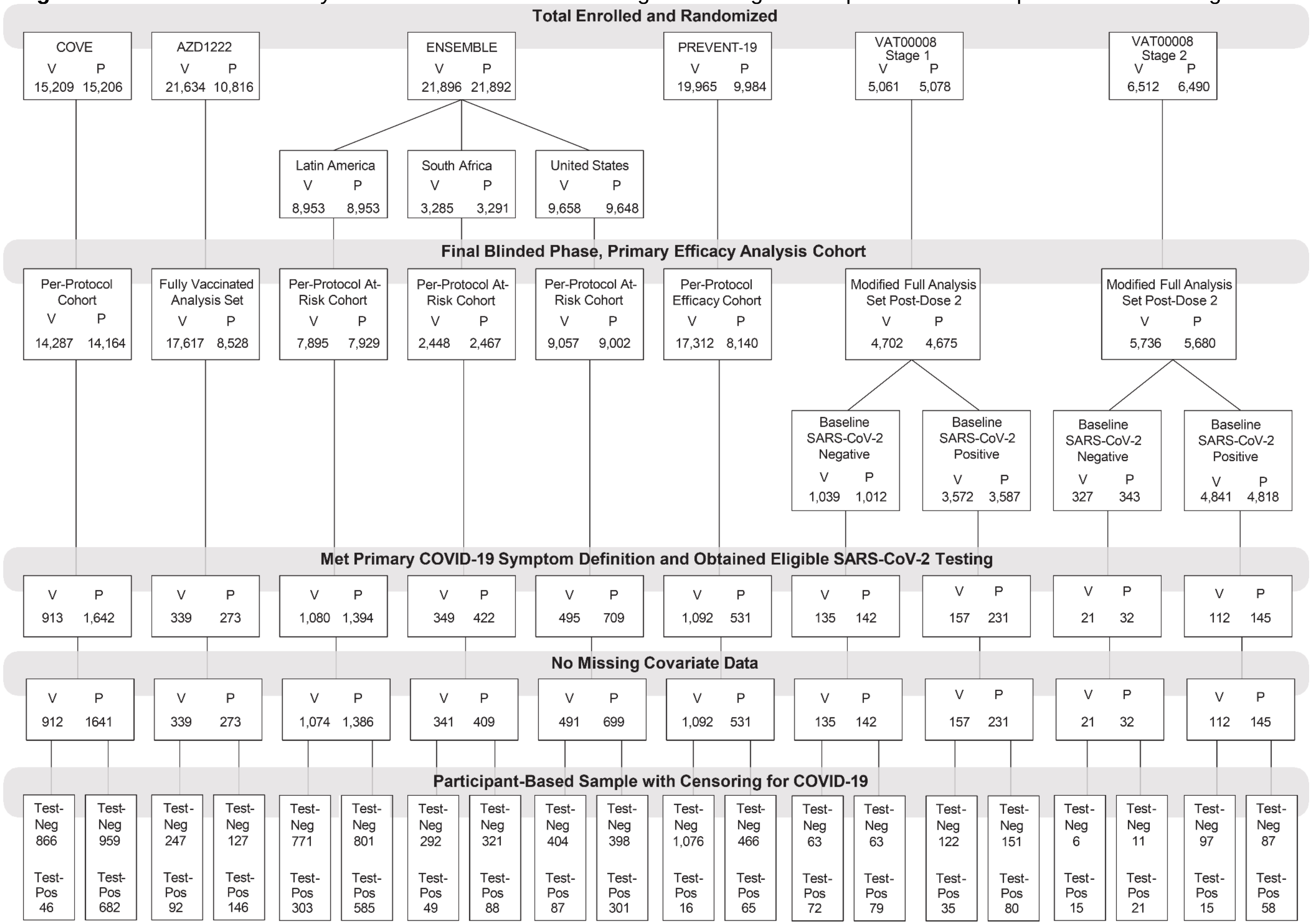

© 2025 Andrews LIB et al. *JAMA Network Open*.

This flow chart describes the participants from five phase 3 COVID-19 Prevention Network RCTs that are eligible for the primary COVID-19 endpoint TND study. For clarity, only SARS-CoV-2 test results from participant-based samples with censoring for COVID-19 (i.e., one test per participant) are shown. V and P represent the number of individuals randomized to receive the vaccine or placebo intervention, respectively. The first level denotes participants who were enrolled and randomized to an intervention in Moderna COVE,[2] AstraZeneca/Oxford AZD1222,[3] Janssen ENSEMBLE,[4] Novavax PREVENT-19,[5] Sanofi/GSK VAT00008 Stage 1,[6] and Sanofi/GSK VAT00008 Stage 2.[7] Participants from Ukrainian sites were excluded due to data completeness issues from the Ukrainian war, as mentioned in the Sanofi/GSK VAT00008 Stage 2 final publication. The second level denotes participants in the final blinded phase, primary efficacy analysis cohorts, which were defined to assess vaccine efficacy against the primary efficacy endpoint in each RCT's final blinded phase analysis publication.[2-7] Most participant exclusions were due to protocol deviations. We assessed vaccine efficacy against primary COVID-19, CDC COVID-19, and non-COVID-19 illness in our study using these cohorts or the subset of each Sanofi/GSK VAT00008 final blinded phase, primary efficacy analysis cohort that had at least 14 days of follow up after their second dose and known baseline SARS-CoV-2 status. The third level denotes participants who obtained a positive or negative SARS-CoV-2 test at least one or two weeks after completing the intervention (depending on RCT protocol), within ten days after symptom onset, after meeting the primary COVID-19 symptom definition, while blinded, and before receiving any non-study COVID-19 vaccinations (i.e., participants eligible to enroll in a TND study). Since RCT protocols instructed participants to frequently report symptoms and obtain SARS-CoV-2 testing, we assumed all RCT participants had identical healthcare-seeking behavior and would have sought testing if they had been ill. Assuming vaccinated and unvaccinated healthcare-seeking individuals have the same probability of obtaining SARS-CoV-2 testing after conditioning on meeting the primary symptom definition and controlling for SARS-CoV-2 test result and additional covariates, the participants enrolled in the TND are representative of the entire healthcare-seeking population. The fourth level denotes participants with complete information on covariates adjusted for in the TND analyses, with race/ethnicity being the only source of missingness. We assumed race/ethnicity data were missing completely at random since few participants were missing data and the probability of missing data was unlikely to be related to other covariates. All participants at this level contribute at least one SARS-CoV-2 test to the primary COVID-19 participant-based sample without censoring for COVID-19, participant-based sample with censoring for COVID-19, specimen-based sample, and random specimen-based sample. The final level denotes the number of participants with a negative SARS-CoV-2 test (i.e., noncases) and a positive SARS-CoV-2 test (i.e., cases) in the participant-based sample with censoring for COVID-19.
Abbreviations: V = Vaccine; P = Placebo; SARS-CoV-2 = Severe Acute Respiratory Syndrome; COVID-19 = Coronavirus Disease of 2019; RCT = Randomized Placebo-Controlled Trial; TND = Test-Negative Design; CDC = Centers for Disease Control and Prevention

© 2025 Andrews LIB et al. *JAMA Network Open*.

eFigure 5. Derivation of CDC COVID-19 End Point Test-Negative Design Participant-Based Samples With Censoring for COVID-19

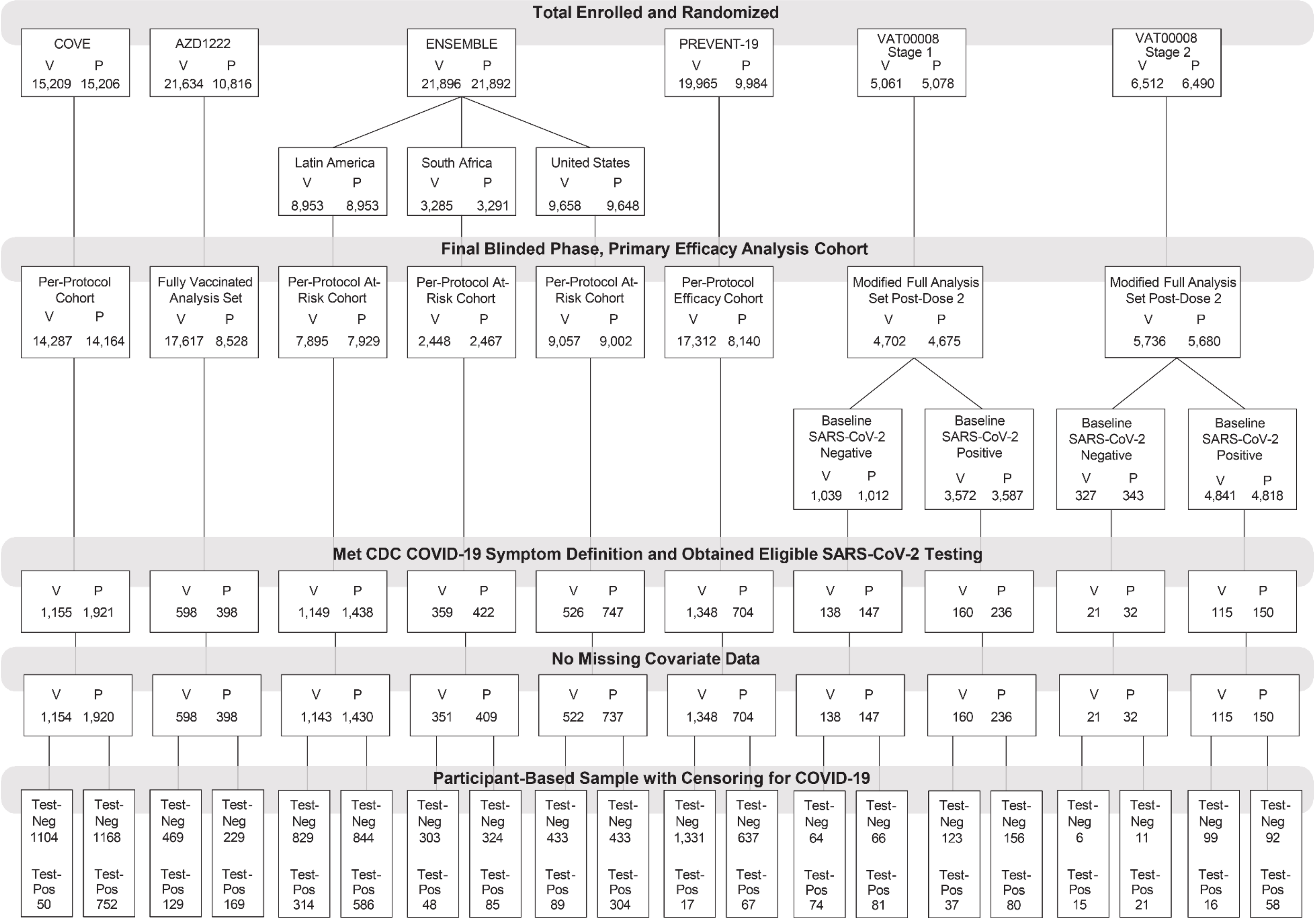

© 2025 Andrews LIB et al. *JAMA Network Open*.

This flow chart describes the participants from five phase 3 COVID-19 Prevention Network RCTs that are eligible for the CDC COVID-19 endpoint TND study. For clarity, only SARS-CoV-2 test results from participant-based samples with censoring for COVID-19 (i.e., one test per participant) are shown. V and P represent the number of individuals randomized to receive the vaccine or placebo intervention, respectively. The first level denotes participants who were enrolled and randomized to an intervention in Moderna COVE,[2] AstraZeneca/Oxford AZD1222,[3] Janssen ENSEMBLE,[4] Novavax PREVENT-19,[5] Sanofi/GSK VAT00008 Stage 1,[6] and Sanofi/GSK VAT00008 Stage 2.[7] Participants from Ukrainian sites were excluded due to data completeness issues from the Ukrainian war, as mentioned in the Sanofi/GSK VAT00008 Stage 2 final publication. The second level denotes participants in the final blinded phase, primary efficacy analysis cohorts, which were defined to assess vaccine efficacy against the primary efficacy endpoint in each RCT's final blinded phase analysis publication.[2-7] Most participant exclusions were due to protocol deviations. We assessed vaccine efficacy against primary COVID-19, CDC COVID-19, and non-COVID-19 illness in our study using these cohorts or the subset of each Sanofi/GSK VAT00008 final blinded phase, primary efficacy analysis cohort that had at least 14 days of follow up after their second dose and known baseline SARS-CoV-2 status. The third level denotes participants who obtained a positive or negative SARS-CoV-2 test at least one or two weeks after completing the intervention (depending on RCT protocol), within ten days after symptom onset, after meeting the CDC COVID-19 symptom definition, while blinded, and before receiving any non-study COVID-19 vaccinations (i.e., participants eligible to enroll in a TND study). Since RCT protocols instructed participants to frequently report symptoms and obtain SARS-CoV-2 testing, we assumed all RCT participants had identical healthcare-seeking behavior and would have sought testing if they had been ill. Assuming vaccinated and unvaccinated healthcare-seeking individuals have the same probability of obtaining SARS-CoV-2 testing after conditioning on meeting the CDC symptom definition and controlling for SARS-CoV-2 test result and additional covariates, the participants enrolled in the TND are representative of the healthcare-seeking population. The fourth level denotes participants with complete information on covariates adjusted for in the TND analyses, with race/ethnicity being the only source of missingness. We assumed race and ethnicity data were missing completely at random since few participants were missing data and the probability of missing data was unlikely to be related to other covariates. All participants at this level contribute at least one SARS-CoV-2 test to the CDC COVID-19 participant-based sample without censoring for COVID-19, participant-based sample with censoring for COVID-19, specimen-based sample, and random specimen-based sample. The final level denotes the number of participants with a negative SARS-CoV-2 test (i.e., noncases) and a positive SARS-CoV-2 test (i.e., cases) in the participant-based sample with censoring for COVID-19.

Abbreviations: V = Vaccine; P = Placebo; SARS-CoV-2 = Severe Acute Respiratory Syndrome; COVID-19 = Coronavirus Disease of 2019; RCT = Randomized Placebo-Controlled Trial; TND = Test-Negative Design; CDC = Centers for Disease Control and Prevention

© 2025 Andrews LIB et al. *JAMA Network Open*.

eFigure 6. CDC COVID-19 Vaccine Efficacy and Semiparametric Logistic Regression Vaccine Effectiveness Estimates by Sampling Method

Sampling Method	Cases	Noncases	CDC COVID−19 VE (95% CI)
	Vaccinated / Total (% Vaccinated)		
**COVE BN**			
RCT	58/865 (7)	14229/27586 (52)	93.4 (91.3, 94.9)
Participant w/o Censoring	50/802 (6)	1107/2320 (48)	93.0 (90.6, 94.9)
Participant w/ Censoring	50/802 (6)	1104/2272 (49)	93.3 (90.9, 95.1)
Specimen	50/802 (6)	1168/2459 (47)	92.9 (90.4, 94.7)
Random Specimen	31/619 (5)	1187/2642 (45)	93.4 (90.5, 95.4)
**AZD1222 BN**			
RCT	177/380 (47)	17440/25765 (68)	61.8 (53.3, 68.8)
Participant w/o Censoring	129/298 (43)	473/707 (67)	62.4 (50.4, 71.5)
Participant w/ Censoring	129/298 (43)	469/698 (67)	62.9 (51.0, 72.0)
Specimen	129/298 (43)	482/716 (67)	63.0 (51.2, 71.9)
Random Specimen	99/242 (41)	512/772 (66)	64.6 (52.5, 73.7)
**ENSEMBLE LA BN**			
RCT	337/972 (35)	7558/14852 (51)	49.4 (42.3, 55.7)
Participant w/o Censoring	314/900 (35)	851/1744 (49)	46.3 (36.3, 54.7)
Participant w/ Censoring	314/900 (35)	829/1673 (50)	48.0 (38.3, 56.2)
Specimen	314/900 (35)	927/1906 (49)	45.6 (35.8, 53.9)
Random Specimen	258/770 (34)	983/2036 (48)	47.7 (37.7, 56.1)
**ENSEMBLE SA BN**			
RCT	61/163 (37)	2387/4752 (50)	40.4 (18.1, 56.6)
Participant w/o Censoring	48/133 (36)	311/653 (48)	39.1 (9.7, 58.9)
Participant w/ Censoring	48/133 (36)	303/627 (48)	40.8 (12.4, 60.0)
Specimen	48/133 (36)	379/810 (47)	37.4 (7.9, 57.4)
Random Specimen	40/113 (35)	387/830 (47)	36.8 (4.1, 58.3)
**ENSEMBLE US BN**			
RCT	94/424 (22)	8963/17635 (51)	72.9 (65.9, 78.4)
Participant w/o Censoring	89/393 (23)	438/883 (50)	70.0 (60.7, 77.1)
Participant w/ Censoring	89/393 (23)	433/866 (50)	70.5 (61.3, 77.5)
Specimen	89/393 (23)	460/926 (50)	69.5 (60.2, 76.6)
Random Specimen	66/330 (20)	483/989 (49)	72.9 (63.7, 79.8)
**PREVENT−19 BN**			
RCT	14/78 (18)	17298/25374 (68)	90.6 (83.2, 94.7)
Participant w/o Censoring	17/84 (20)	1331/1971 (68)	88.0 (79.9, 92.8)
Participant w/ Censoring	17/84 (20)	1331/1968 (68)	88.1 (80.0, 92.9)
Specimen	17/84 (20)	1377/2043 (67)	87.9 (79.8, 92.7)
Random Specimen	13/79 (16)	1381/2048 (67)	90.5 (83.2, 94.6)
**VAT00008 S1 BN**			
RCT	83/172 (48)	956/1879 (51)	15.9 (−13.4, 37.7)
Participant w/o Censoring	74/155 (48)	72/142 (51)	33.1 (−8.7, 58.8)
Participant w/ Censoring	74/155 (48)	64/130 (49)	29.2 (−16.7, 57.1)
Specimen	74/155 (48)	79/157 (50)	33.1 (−7.7, 58.4)
Random Specimen	70/145 (48)	83/167 (50)	21.2 (−26.8, 51.0)
**VAT00008 S1 BP**			
RCT	46/136 (34)	3525/7021 (50)	51.6 (30.9, 66.0)
Participant w/o Censoring	37/117 (32)	128/287 (45)	53.5 (17.4, 73.8)
Participant w/ Censoring	37/117 (32)	123/279 (44)	50.6 (13.8, 71.6)
Specimen	37/117 (32)	148/323 (46)	49.8 (13.2, 71.0)
Random Specimen	31/98 (32)	154/342 (45)	52.3 (17.8, 72.3)
**VAT00008 S2 BN**			
RCT	15/37 (41)	300/611 (49)	30.0 (−35.0, 63.7)
Participant w/o Censoring	15/36 (42)	7/18 (39)	−27.6 (−323.8, 61.6)
Participant w/ Censoring	15/36 (42)	6/17 (35)	−198.7 (−805.8, 1.5)
Specimen	15/36 (42)	7/18 (39)	−44.1 (−405.7, 58.9)
Random Specimen	15/34 (44)	7/20 (35)	−183.6 (−1082.3, 32.0)
**VAT00008 S2 BP**			
RCT	15/77 (19)	4504/8956 (50)	76.1 (58.1, 86.4)
Participant w/o Censoring	16/74 (22)	100/196 (51)	66.4 (34.8, 82.7)
Participant w/ Censoring	16/74 (22)	99/191 (52)	67.4 (37.4, 83.0)
Specimen	16/74 (22)	104/202 (51)	65.6 (34.0, 82.0)
Random Specimen	10/53 (19)	110/223 (49)	68.0 (34.1, 84.5)

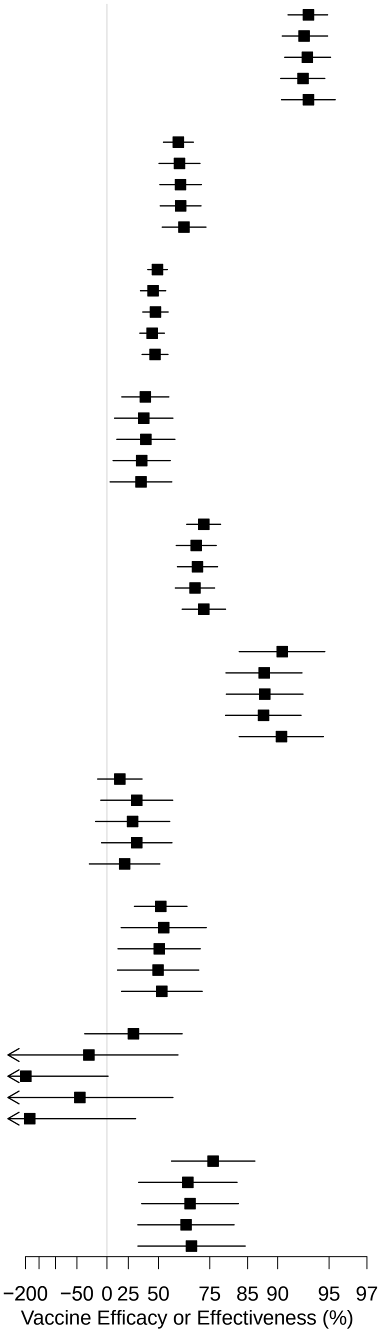

CDC COVID-19 RCT vaccine efficacy was defined as one minus the COVID-19 hazard ratio (vaccine vs. placebo), estimated using an unadjusted Cox proportional hazards model and Efron's method for handling ties.[8]
CDC COVID-19 TND vaccine effectiveness was defined as one minus the COVID-19 risk ratio (vaccine vs. placebo), estimated using targeted maximum likelihood estimation under a semiparametric partially linear logistic regression model.[18] A partially linear

© 2025 Andrews LIB et al. *JAMA Network Open*.

first-order smooth highly adaptive lasso that flexibly adjusts for age, sex, race/ethnicity, region, comorbidities, and two-week testing date intervals was applied for estimation and allowed for two-way interactions.
Estimates and 95% confidence intervals are compared on the multiplicative natural logarithm scale, ln(1-VE), with plotting labels on the VE scale.
Abbreviations: CDC = Centers for Disease Control and Prevention; COVID-19 = Coronavirus Disease of 2019; RCT = Randomized Placebo-Controlled Trial; TND = Test-Negative Design; VE = Vaccine Efficacy or Vaccine Effectiveness; CI = Confidence Interval; w/o = without; w/ = with; LA = Latin America; SA = South Africa; US = United States; S1 = Stage 1; S2 = Stage 2; BN = Baseline SARS-CoV-2 Negative; BP = Baseline SARS-CoV-2 Positive; SARS-CoV-2 = Severe Acute Respiratory Syndrome Coronavirus 2; HR = Hazard Ratio; RR = Risk Ratio; ln = natural logarithm

© 2025 Andrews LIB et al. *JAMA Network Open*.

eFigure 7. Primary COVID-19 Vaccine Efficacy and Ordinary Logistic Regression Vaccine Effectiveness Estimates by Sampling Method

Sampling Method	Cases	Noncases	Primary COVID−19 VE (95% CI)
	Vaccinated / Total (% Vaccinated)		
**COVE BN**			
RCT	55/799 (7)	14232/27652 (51)	93.2 (91.0, 94.8)
Participant w/o Censoring	46/728 (6)	869/1859 (47)	92.7 (89.9, 94.7)
Participant w/ Censoring	46/728 (6)	866/1825 (47)	92.9 (90.3, 94.9)
Specimen	46/728 (6)	916/1963 (47)	92.5 (89.7, 94.6)
Random Specimen	25/558 (4)	937/2133 (44)	94.1 (91.1, 96.1)
**AZD1222 BN**			
RCT	141/325 (43)	17476/25820 (68)	67.0 (58.9, 73.5)
Participant w/o Censoring	92/238 (39)	250/377 (66)	67.4 (53.4, 77.1)
Participant w/ Censoring	92/238 (39)	247/374 (66)	66.9 (52.8, 76.8)
Specimen	92/238 (39)	253/381 (66)	67.5 (53.7, 77.2)
Random Specimen	71/194 (37)	274/425 (64)	67.4 (52.6, 77.5)
**ENSEMBLE LA BN**			
RCT	329/963 (34)	7566/14861 (51)	49.7 (42.4, 56.1)
Participant w/o Censoring	303/888 (34)	795/1649 (48)	46.7 (36.6, 55.3)
Participant w/ Censoring	303/888 (34)	771/1572 (49)	48.5 (38.6, 56.9)
Specimen	303/888 (34)	865/1797 (48)	46.3 (36.3, 54.7)
Random Specimen	246/751 (33)	922/1934 (48)	48.3 (38.0, 56.8)
**ENSEMBLE SA BN**			
RCT	62/172 (36)	2386/4743 (50)	44.0 (22.9, 59.7)
Participant w/o Censoring	49/137 (36)	301/635 (47)	37.8 (5.3, 59.2)
Participant w/ Censoring	49/137 (36)	292/613 (48)	37.7 (5.0, 59.1)
Specimen	49/137 (36)	362/782 (46)	33.3 (−0.7, 55.8)
Random Specimen	37/106 (35)	374/813 (46)	36.1 (−0.8, 59.5)
**ENSEMBLE US BN**			
RCT	93/416 (22)	8964/17643 (51)	72.9 (65.7, 78.7)
Participant w/o Censoring	87/388 (22)	409/818 (50)	70.8 (61.3, 77.9)
Participant w/ Censoring	87/388 (22)	404/802 (50)	71.0 (61.6, 78.1)
Specimen	87/388 (22)	429/855 (50)	71.0 (61.6, 78.1)
Random Specimen	69/328 (21)	447/915 (49)	71.0 (60.9, 78.6)
**PREVENT−19 BN**			
RCT	14/77 (18)	17298/25375 (68)	90.4 (82.9, 94.6)
Participant w/o Censoring	16/81 (20)	1076/1545 (70)	88.3 (79.4, 93.4)
Participant w/ Censoring	16/81 (20)	1076/1542 (70)	88.4 (79.4, 93.4)
Specimen	16/81 (20)	1098/1580 (69)	88.2 (79.1, 93.3)
Random Specimen	12/76 (16)	1102/1585 (70)	91.2 (83.3, 95.3)
**VAT00008 S1 BN**			
RCT	81/169 (48)	958/1882 (51)	15.3 (−15.8, 38.2)
Participant w/o Censoring	72/151 (48)	70/137 (51)	38.7 (−12.2, 66.5)
Participant w/ Censoring	72/151 (48)	63/126 (50)	32.8 (−24.5, 63.7)
Specimen	72/151 (48)	77/152 (51)	36.8 (−13.0, 64.6)
Random Specimen	69/144 (48)	80/159 (50)	32.0 (−20.5, 61.6)
**VAT00008 S1 BP**			
RCT	43/133 (32)	3528/7024 (50)	52.7 (31.2, 67.9)
Participant w/o Censoring	35/115 (30)	125/279 (45)	55.5 (14.7, 76.8)
Participant w/ Censoring	35/115 (30)	122/273 (45)	55.3 (14.4, 76.6)
Specimen	35/115 (30)	145/315 (46)	52.9 (12.5, 74.6)
Random Specimen	30/96 (31)	150/334 (45)	49.9 (6.5, 73.2)
**VAT00008 S2 BN**			
RCT	15/37 (41)	300/611 (49)	30.9 (−39.3, 66.7)
Participant w/o Censoring	15/36 (42)	7/18 (39)	−56.6 (−682.5, 68.7)
Participant w/ Censoring	15/36 (42)	6/17 (35)	−170.0 (−1628.3, 57.8)
Specimen	15/36 (42)	7/18 (39)	−56.6 (−682.5, 68.7)
Random Specimen	15/34 (44)	7/20 (35)	−204.8 (−1665.1, 47.4)
**VAT00008 S2 BP**			
RCT	16/79 (20)	4503/8954 (50)	75.1 (56.3, 86.6)
Participant w/o Censoring	15/73 (21)	98/189 (52)	69.3 (34.6, 85.6)
Participant w/ Censoring	15/73 (21)	97/184 (53)	70.6 (37.2, 86.2)
Specimen	15/73 (21)	102/195 (52)	68.3 (32.7, 85.1)
Random Specimen	12/57 (21)	105/211 (50)	60.9 (16.4, 81.7)

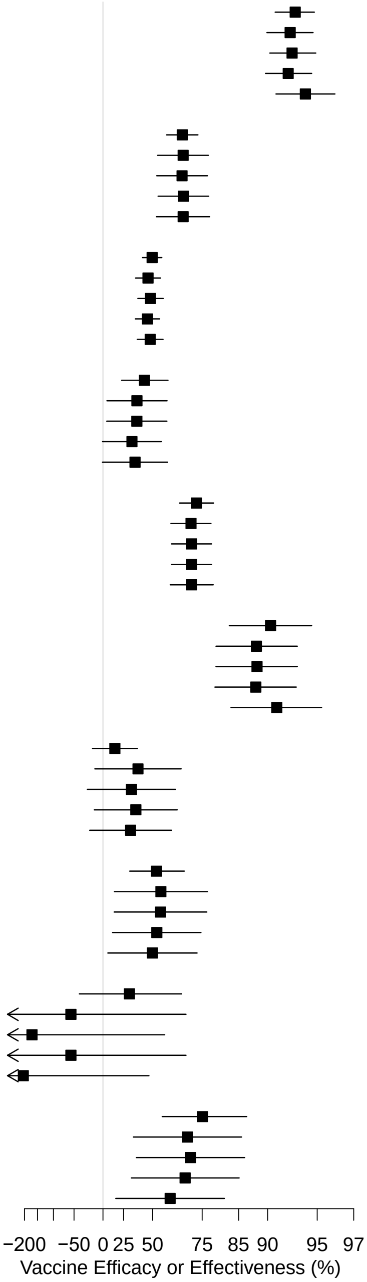

Primary COVID-19 RCT vaccine efficacies were estimated using the primary statistical approach defined in each trial's final blinded phase analysis publications. Moderna COVE vaccine efficacy was defined as one minus the COVID-19 hazard ratio (vaccine vs. placebo), estimated using a Cox proportional hazards model stratified on randomization factors and using Efron's method for handling ties.[2,8] AstraZeneca/Oxford AZD1222 vaccine efficacy was defined as one minus the COVID-19 risk ratio (vaccine vs. placebo), estimated using a Poisson regression model and robust standard errors, adjusting for study arm, age group (< 65 and ≥ 65 years), and follow-up time.[3,9] Janssen ENSEMBLE region-specific vaccine efficacies were defined as one minus the ratio of COVID-19 incidence rates (vaccine vs. placebo), estimated using exact Poisson regression.[4,10] Novavax PREVENT-19 vaccine efficacy was defined as one minus the COVID-19 risk ratio (vaccine vs. placebo), estimated using a Poisson regression model and robust standard errors,[9] adjusting for study arm and age group (< 65 and ≥ 65 years).[5] Sanofi/GSK VAT00008 trial cohorts' vaccine

© 2025 Andrews LIB et al. *JAMA Network Open*.

efficacies were defined as one minus the ratio of COVID-19 incidence rates per 1000 person-years (vaccine vs. placebo), with confidence intervals calculated by the exact method,[11] assuming vaccinated cases follow a binomial distribution after conditioning on total cases.[6,7]
Primary COVID-19 TND vaccine effectiveness was defined as one minus the COVID-19 risk ratio (vaccine vs. placebo), estimated using an ordinary logistic regression model, adjusted for age, sex, race/ethnicity, region, comorbidities, and two-week testing date linear main effects.
Estimates and 95% confidence intervals are compared on the multiplicative natural logarithm scale, ln(1-VE), with plotting labels on the VE scale.
Abbreviations: COVID-19 = Coronavirus Disease of 2019; RCT = Randomized Placebo-Controlled Trial; TND = Test-Negative Design; VE = Vaccine Efficacy or Vaccine Effectiveness; CI = Confidence Interval; w/o = without; w/ = with; LA = Latin America; SA = South Africa; US = United States; S1 = Stage 1; S2 = Stage 2; BN = Baseline SARS-CoV-2 Negative; BP = Baseline SARS-CoV-2 Positive; SARS-CoV-2 = Severe Acute Respiratory Syndrome Coronavirus 2; ln = natural logarithm

© 2025 Andrews LIB et al. *JAMA Network Open*.

eFigure 8. CDC COVID-19 Vaccine Efficacy and Ordinary Logistic Regression Vaccine Effectiveness Estimates by Sampling Method

Sampling Method	Cases	Noncases	CDC COVID−19 VE (95% CI)
	Vaccinated / Total (% Vaccinated)		
**COVE BN**			
RCT	58/865 (7)	14229/27586 (52)	93.4 (91.3, 94.9)
Participant w/o Censoring	50/802 (6)	1107/2320 (48)	93.1 (90.7, 94.9)
Participant w/ Censoring	50/802 (6)	1104/2272 (49)	93.4 (91.0, 95.1)
Specimen	50/802 (6)	1168/2459 (47)	92.9 (90.4, 94.8)
Random Specimen	31/619 (5)	1187/2642 (45)	93.5 (90.6, 95.5)
**AZD1222 BN**			
RCT	177/380 (47)	17440/25765 (68)	61.8 (53.3, 68.8)
Participant w/o Censoring	129/298 (43)	473/707 (67)	62.4 (49.7, 71.9)
Participant w/ Censoring	129/298 (43)	469/698 (67)	63.4 (50.9, 72.7)
Specimen	129/298 (43)	482/716 (67)	62.8 (50.2, 72.2)
Random Specimen	99/242 (41)	512/772 (66)	64.7 (51.9, 74.1)
**ENSEMBLE LA BN**			
RCT	337/972 (35)	7558/14852 (51)	49.4 (42.3, 55.7)
Participant w/o Censoring	314/900 (35)	851/1744 (49)	46.0 (35.8, 54.6)
Participant w/ Censoring	314/900 (35)	829/1673 (50)	47.6 (37.7, 55.9)
Specimen	314/900 (35)	927/1906 (49)	45.3 (35.3, 53.8)
Random Specimen	258/770 (34)	983/2036 (48)	47.4 (37.3, 56.0)
**ENSEMBLE SA BN**			
RCT	61/163 (37)	2387/4752 (50)	40.4 (18.1, 56.6)
Participant w/o Censoring	48/133 (36)	311/653 (48)	41.3 (10.4, 61.6)
Participant w/ Censoring	48/133 (36)	303/627 (48)	42.9 (12.6, 62.7)
Specimen	48/133 (36)	379/810 (47)	37.0 (4.8, 58.3)
Random Specimen	40/113 (35)	387/830 (47)	36.5 (1.6, 59.0)
**ENSEMBLE US BN**			
RCT	94/424 (22)	8963/17635 (51)	72.9 (65.9, 78.4)
Participant w/o Censoring	89/393 (23)	438/883 (50)	69.9 (60.3, 77.1)
Participant w/ Censoring	89/393 (23)	433/866 (50)	70.2 (60.7, 77.4)
Specimen	89/393 (23)	460/926 (50)	69.9 (60.4, 77.1)
Random Specimen	66/330 (20)	483/989 (49)	73.4 (63.9, 80.3)
**PREVENT−19 BN**			
RCT	14/78 (18)	17298/25374 (68)	90.6 (83.2, 94.7)
Participant w/o Censoring	17/84 (20)	1331/1971 (68)	87.5 (78.3, 92.8)
Participant w/ Censoring	17/84 (20)	1331/1968 (68)	87.6 (78.4, 92.8)
Specimen	17/84 (20)	1377/2043 (67)	87.4 (78.1, 92.7)
Random Specimen	13/79 (16)	1381/2048 (67)	90.1 (81.8, 94.6)
**VAT00008 S1 BN**			
RCT	83/172 (48)	956/1879 (51)	15.9 (−13.4, 37.7)
Participant w/o Censoring	74/155 (48)	72/142 (51)	40.9 (−8.5, 67.8)
Participant w/ Censoring	74/155 (48)	64/130 (49)	34.1 (−22.5, 64.5)
Specimen	74/155 (48)	79/157 (50)	38.8 (−9.5, 65.8)
Random Specimen	70/145 (48)	83/167 (50)	27.9 (−26.9, 59.0)
**VAT00008 S1 BP**			
RCT	46/136 (34)	3525/7021 (50)	51.6 (30.9, 66.0)
Participant w/o Censoring	37/117 (32)	128/287 (45)	52.7 (10.7, 75.0)
Participant w/ Censoring	37/117 (32)	123/279 (44)	52.2 (9.7, 74.7)
Specimen	37/117 (32)	148/323 (46)	49.7 (7.5, 72.6)
Random Specimen	31/98 (32)	154/342 (45)	50.1 (8.2, 72.9)
**VAT00008 S2 BN**			
RCT	15/37 (41)	300/611 (49)	30.0 (−35.0, 63.7)
Participant w/o Censoring	15/36 (42)	7/18 (39)	−56.6 (−682.5, 68.7)
Participant w/ Censoring	15/36 (42)	6/17 (35)	−170.0 (−1628.3, 57.8)
Specimen	15/36 (42)	7/18 (39)	−56.6 (−682.5, 68.7)
Random Specimen	15/34 (44)	7/20 (35)	−230.0 (−1898.7, 45.5)
**VAT00008 S2 BP**			
RCT	15/77 (19)	4504/8956 (50)	76.1 (58.1, 86.4)
Participant w/o Censoring	16/74 (22)	100/196 (51)	67.7 (32.8, 84.5)
Participant w/ Censoring	16/74 (22)	99/191 (52)	68.8 (35.0, 85.0)
Specimen	16/74 (22)	104/202 (51)	66.8 (31.3, 84.0)
Random Specimen	10/53 (19)	110/223 (49)	68.8 (30.5, 86.0)

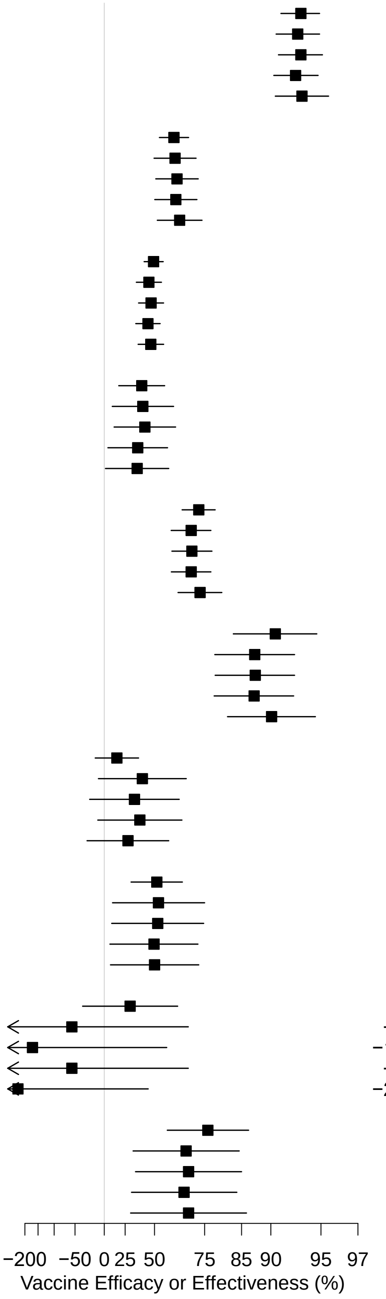

CDC COVID-19 RCT vaccine efficacy was defined as one minus the COVID-19 hazard ratio (vaccine vs. placebo), estimated using an unadjusted Cox proportional hazards model and Efron's method for handling ties.[8]
CDC COVID-19 TND vaccine effectiveness was defined as one minus the COVID-19 risk ratio (vaccine vs. placebo), estimated using an ordinary logistic regression, adjusted for age, sex, race/ethnicity, region, comorbidities, and two-week testing date linear main effects.
Estimates and 95% confidence intervals are compared on the multiplicative natural logarithm scale, ln(1-VE), with plotting labels on the VE scale.

© 2025 Andrews LIB et al. *JAMA Network Open*.

Abbreviations: CDC = Centers for Disease Control and Prevention; COVID-19 = Coronavirus Disease of 2019; RCT = Randomized Placebo-Controlled Trial; TND = Test-Negative Design; VE = Vaccine Efficacy or Vaccine Effectiveness; CI = Confidence Interval; w/o = without; w/ = with; LA = Latin America; SA = South Africa; US = United States; S1 = Stage 1; S2 = Stage 2; BN = Baseline SARS-CoV-2 Negative; BP = Baseline SARS-CoV-2 Positive; SARS-CoV-2 = Severe Acute Respiratory Syndrome Coronavirus 2; ln = natural logarithm

© 2025 Andrews LIB et al. *JAMA Network Open*.

eFigure 9. Randomized Placebo-Controlled Trial Vaccine Efficacy Estimates vs Test-Negative Design Ordinary Logistic Regression Vaccine Effectiveness Estimates

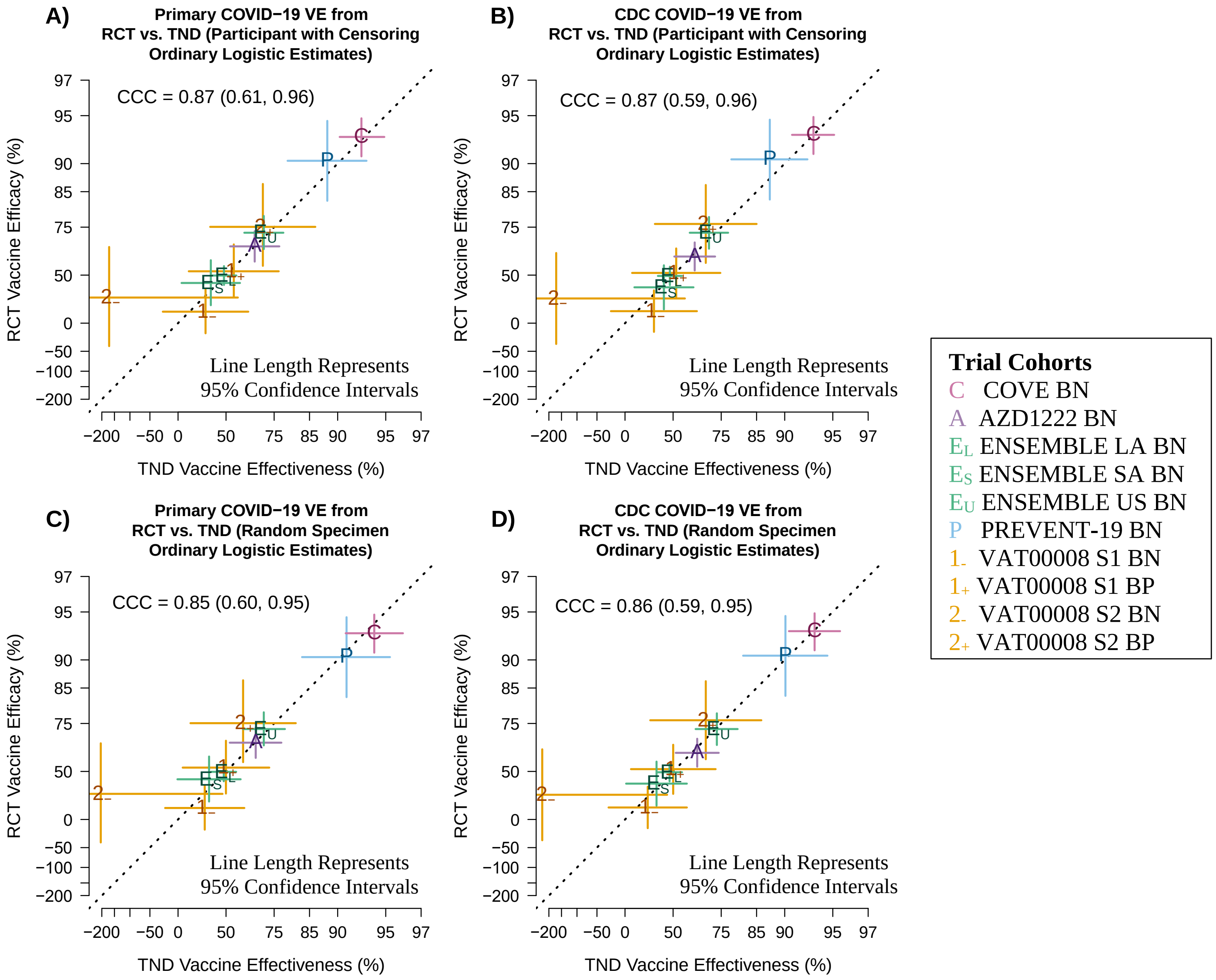

A) Primary COVID-19 vaccine efficacy estimates from RCT cohorts and ordinary logistic regression vaccine effectiveness estimates from primary COVID-19 TND participant-based samples with censoring for COVID-19. **B)** CDC COVID-19 vaccine efficacy estimates from RCT cohorts and ordinary logistic regression vaccine effectiveness estimates from CDC COVID-19 TND participant-based samples with censoring for COVID-19. **C)** Primary COVID-19 vaccine efficacy estimates from RCT cohorts and ordinary logistic regression vaccine effectiveness estimates from the primary COVID-19 TND random specimen-based samples. **D)** CDC COVID-19 vaccine efficacy estimates from RCT cohorts and ordinary logistic regression vaccine effectiveness estimates from CDC COVID-19 TND random specimen-based samples.

Vaccine efficacy and effectiveness estimates were computed from ten phase 3 RCT final blinded phase, primary efficacy analysis cohorts: Moderna COVE BN, AstraZeneca/Oxford AZD1222 BN, Janssen ENSEMBLE BN (analyzed separately as Latin America, South Africa, and United States), Novavax PREVENT-19 BN, and Sanofi/GSK VAT00008 (analyzed separately by Stage 1 monovalent and Stage 2 bivalent vaccine and by baseline SARS-CoV-2 negative and positive status). RCT vaccine efficacy results were derived using trials' final blinded phase, primary efficacy analysis approaches for primary COVID-19 and unadjusted Cox proportional hazards models for CDC COVID-19. TND vaccine effectiveness was estimated using an ordinary logistic regression adjusted for age, sex, race/ethnicity, region, comorbidities, and two-week testing date linear main effects. Estimates (symbols) and 95% confidence intervals (vertical and horizontal line segments) are compared on the multiplicative natural logarithm scale, ln(1-VE), with plotting labels on the VE scale. The 95% confidence interval lower bounds for Sanofi Stage 2 BN TND estimates extend beyond the plotting region. Concordance correlation coefficient estimates and 95% confidence intervals via Z-transformation are reported.

© 2025 Andrews LIB et al. *JAMA Network Open*.

Abbreviations: COVID-19 = Coronavirus Disease 2019; VE = Vaccine Efficacy or Vaccine Effectiveness; RCT = Randomized Placebo-Controlled Trial; TND = Test-Negative Design; CCC = Concordance Correlation Coefficient; CDC = Centers for Disease Control and Prevention; LA = Latin America; SA: South Africa; US = United States; S1 = Stage 1; S2 = Stage 2; BN = Baseline SARS-CoV-2 Negative; BP = Baseline SARS-CoV-2 Positive; ln = natural logarithm; SARS-CoV-2 = Severe Acute Respiratory Syndrome Coronavirus 2

© 2025 Andrews LIB et al. *JAMA Network Open*.

eFigure 10. Semiparametric Logistic Regression and Ordinary Logistic Regression Bias and Variance Across All Trial Cohorts

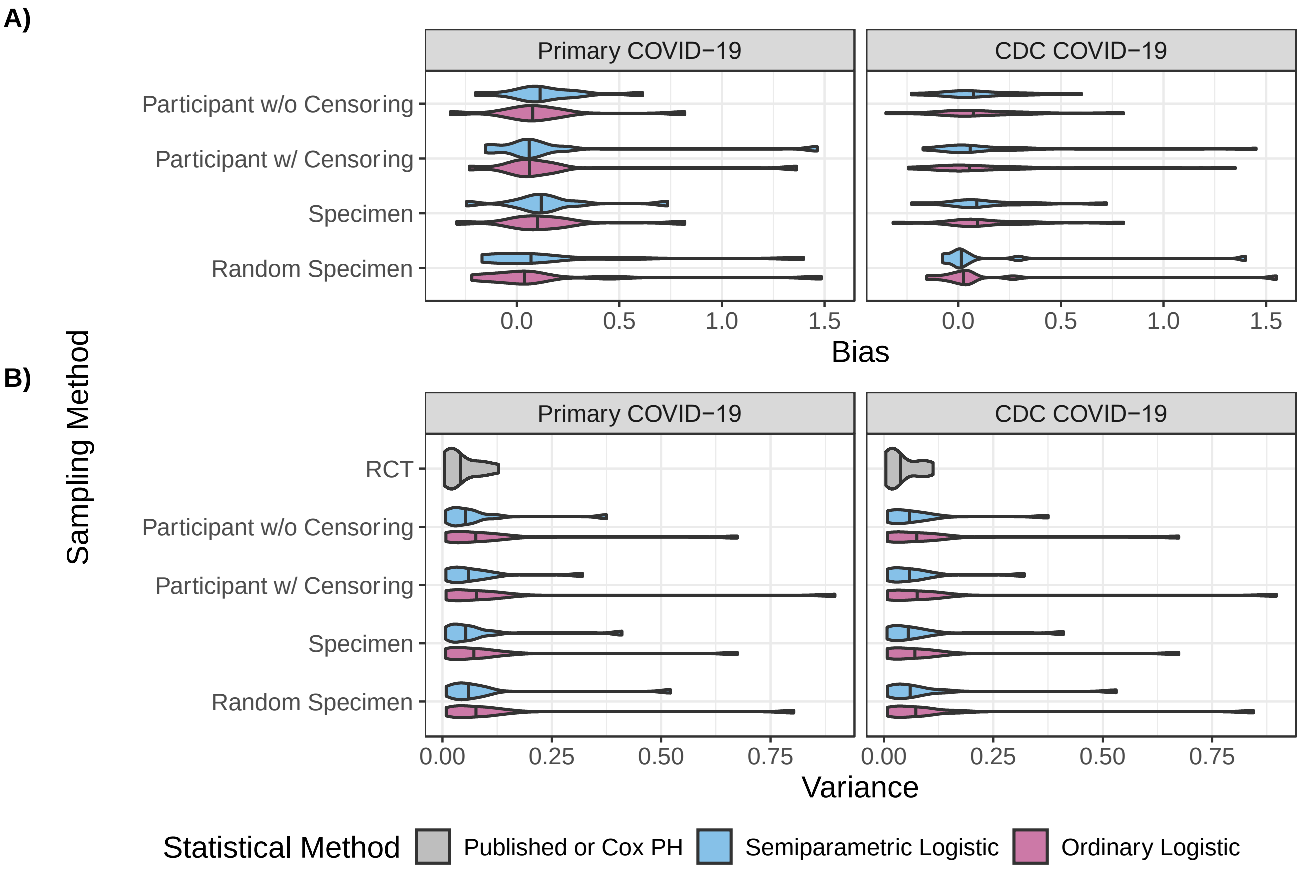

A) For each trial cohort, the biases in the TND vaccine effectiveness estimates derived from the semiparametric logistic regression and ordinary logistic regression were computed using $\ln(1-\widehat{VE}_{TND}) - \ln(1-\widehat{VE}_{RCT})$, with RCT vaccine efficacy estimates as the ground truth. The bias distributions are displayed as violin plots for the primary and CDC COVID-19 definitions and the four TND sampling methods. The black vertical line in each violin plot represents the median bias. **B)** For each trial cohort, the estimated variances of $\ln(1-\widehat{VE}_{TND})$ derived from the semiparametric logistic regression and ordinary logistic regression were computed. The estimated variance of $\ln(1-\widehat{VE}_{RCT})$ was approximated, assuming all published RCT $\widehat{VE}$ confidence intervals were Wald 95% confidence intervals for simplicity. The estimated variance distributions are displayed as violin plots for the primary and CDC COVID-19 definitions and the RCT and TND sampling methods. The black vertical line in each violin plot represents the median estimated variance.

Abbreviations: COVID-19 = Coronavirus Disease 2019; CDC = Centers for Disease Control and Prevention; w/o = without; w/ = with; RCT = Randomized Placebo-Controlled Trial; PH: Proportional Hazards; TND = Test-Negative Design; VE = Vaccine Efficacy or Vaccine Effectiveness

© 2025 Andrews LIB et al. *JAMA Network Open*.

eFigure 11. Uniform Quantile-Quantile Plots of *P* Values Overall and by Age Subgroups to Assess Noncase Exchangeability Violations

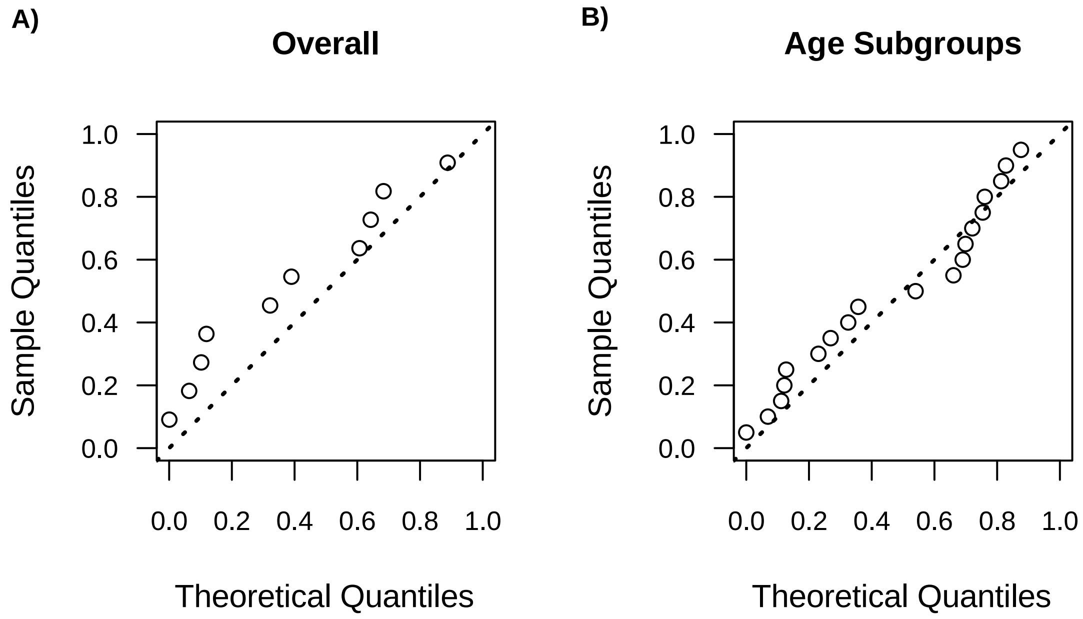

A) Probability plot with uniform theoretical quantiles of the ten p-values from the Cox proportional hazard models of non-COVID-19 illness by vaccination status for each trial cohort overall. **B)** Probability plot with uniform theoretical quantiles of the 20 p-values from the Cox proportional hazard models of non-COVID-19 illness by vaccination status for each trial cohort age subgroup.
If none of the COVID-19 vaccines affect non-COVID-19 illness, the p-values should follow a uniform distribution and adhere to the identity line in the uniform quantile-quantile plots.

© 2025 Andrews LIB et al. *JAMA Network Open*.

eMethods

Semiparametric Logistic Regression Approach

We extended a recently developed semiparametric logistic approach developed by van der Laan and Gilbert[18] to analyze data from a test-negative design study. This section summarizes the estimand of interest, identifiability assumptions, estimation, and inference for this approach.

Causal Question

Suppose we are interested in assessing the effect of a binary exposure variable on virologically confirmed, symptomatic COVID-19 in a healthcare-seeking population. We could investigate various binary vaccine regimens, such as completing a primary COVID-19 vaccination series (vs. not receiving any COVID-19 vaccines), receiving an mRNA vs. protein primary vaccination series, or being up to date with all COVID-19 vaccines (vs. receiving all but the most recent COVID-19 vaccine). Other exposure variables of interest may be defined by immunological biomarkers measured at the time of SARS-CoV-2 viral testing, such as anti-SARS-CoV-2 antibodies or T cells, to further mechanistic understanding of COVID-19 surrogate endpoints (immune correlates of protection).[19] This supplement focuses on comparing individuals who completed a given COVID-19 primary vaccination series to those who did not receive any COVID-19 vaccine, as investigated in the TND study described in our manuscript. Assumptions and generalizability should be reassessed for every TND study and application.

Our population of interest is a healthcare-seeking population, which we define as independent and identically distributed individuals that would seek care if ill. We assume all individuals in this population have the same probability of seeking SARS-CoV-2 testing if they experienced COVID-19-like symptoms. An individual in this population has the unobserved full data structure $O_F = (A, C, Y, X)$, generated by data-generating distribution P_F, where A is an indicator of vaccination, C is an indicator of meeting the symptom definition, Y is an indicator of testing SARS-CoV-2 positive if they were to obtain testing, and $X \in \mathbb{R}^d$ is a vector of demographic and clinical covariates. Individuals also have the causal data structure $O_{F,ca} = (O_F, Y(0), Y(1), C(0), C(1))$, where $Y(a)$ and $C(a)$ represent the potential outcomes for the SARS-CoV-2 test result (if they were to obtain testing) and meeting the symptom definition, respectively, of an individual with vaccination status a. In this causal framework, every individual has potential outcomes for both vaccination statuses but only one set of observed values that may or may not coincide with their hypothetical statuses. Our estimand of interest is a full data causal conditional risk ratio,

$$RR_{F,ca}\left(P_{F,ca}\right)(x) = \frac{P_{F,ca}(Y(1) = 1, C(1) = 1 | X = x)}{P_{F,ca}(Y(0) = 1, C(0) = 1 | X = x)},$$

which represents the risk reduction of virologically confirmed, symptomatic COVID-19 for vaccinated vs. unvaccinated healthcare-seeking individuals with the same covariates. We target the conditional risk ratio rather than the marginal risk ratio that averages across individual characteristics. Because the characteristics of the observed TND study sample may differ from the healthcare-seeking population, we may not be able to identify the marginal risk ratio. Additionally, if desired, we can identify subgroup-specific associations between vaccination and COVID-19 using a conditional risk ratio that may generalize better to subpopulations with differing characteristics than a marginal risk ratio.

Observed TND Data

For our observed TND study sample of n participants, we define R as the indicator of obtaining an eligible SARS-CoV-2 test (e.g., after meeting the symptom definition and within 10 days of symptom onset) and Δ as the indicator of observing vaccination status A (i.e., not missing). We introduce Δ because our statistical approach can handle TND studies that include participants who are missing A at random,[20] though in our study all participants' vaccination statuses are known. Since TND studies only sample individuals who meet the symptom definition ($C = 1$) and obtain an eligible SARS-CoV-2 test ($R = 1$), an individual's observed data structure according to this TND is $O = CR(\Delta, \Delta A, Y, X)$ and is generated by data-generating distribution P (eFigure 1 in Supplement 1). Thus, TND cases meet the symptom definition, obtain SARS-CoV-2 testing, and test SARS-CoV-2 positive, such that $O = (\Delta, \Delta A, 1, X)$. TND noncases meet the symptom definition, obtain SARS-CoV-2 testing, and test SARS-CoV-2

© 2025 Andrews LIB et al. *JAMA Network Open*.

negative, such that $O = (\Delta, \Delta A, 0, X)$. Individuals who do not meet the symptom definition ($C = 0$) or do not obtain an eligible SARS-CoV-2 test ($R = 0$) are not enrolled in a TND study.

All information in a TND can be collected at TND enrollment, or time of SARS-CoV-2 testing. In our study, X included age, sex, race, ethnicity, region, comorbidities, and SARS-CoV-2 testing dates in 2-week testing date bins (eTable 2 in Supplement 1). Most covariates were collected at RCT enrollment, which is not a meaningful timepoint in an observational TND study. Thus, we treated these covariates as if they were collected at the time of TND enrollment since the RCTs had relatively short follow-up.

Identifying Assumptions

While we are interested in our target estimand, $RR_{F,ca}(P_{F,ca})(x)$, we cannot obtain this estimand directly from the observed TND data. First, observed TND data only consist of individuals who obtain SARS-CoV-2 testing ($R = 1$) and may have incomplete vaccination status information ($\Delta = 0$), though missing vaccination status is not a concern in our TND study. Additionally, TND sampling schemes are outcome-dependent, meaning the proportion of COVID-19 cases in a TND study sample (e.g., individuals with COVID-19-like symptoms) likely differs from the proportion of COVID-19 cases in the entire healthcare-seeking population. Because of this sampling scheme, we can directly estimate the probability of vaccination given COVID-19 from a TND study sample but not the probability of COVID-19 given vaccination.[21] Consequently, from our observed TND data, we can obtain the following observed data conditional odds ratio.,

$$OR(P)(x) = \frac{P(A = 1|Y = 1, X = x, C = 1, R = 1, \Delta = 1)/P(A = 0|Y = 1, X = x, C = 1, R = 1, \Delta = 1)}{P(A = 1|Y = 0, X = x, C = 1, R = 1, \Delta = 1)/P(A = 0|Y = 0, X = x, C = 1, R = 1, \Delta = 1)}.$$

To identify the full data causal conditional risk ratio, we must introduce the following identifying assumptions such that the observed data estimand $OR(P)(x)$ equals the full data conditional risk ratio $RR_F(P_F)(x)$.[18,22-25] These identifying assumptions are nonparametric, as they do not instill model restrictions on P or P_F. The symbol $\perp\!\!\!\perp$ denotes that two variables are independent of each other, conditional on other covariates.

1. $P(1 > P(Y = 1|C = 1, X, R = 1) > 0) = 1$
2. $P(P(\Delta = 1|Y, C = 1, X, R = 1) > 0) = 1$
3. $P_F(P_F(\mathrm{R} = 1|Y, C = 1, X) > 0) = 1$
4. $R \perp\!\!\!\perp A|Y, C = 1, \mathrm{X}$
5. $\Delta \perp\!\!\!\perp A|Y, C = 1, X, R = 1$
6. No SARS-CoV-2 Test Result (Y) Misclassification
7. No Vaccination Status (A) Misclassification
8. $P_F(Y = 0, C = 1 \mid A = 1, X) = P_F(Y = 0, C = 1 \mid A = 0, X)$ (Noncase Exchangeability)

The first assumption requires that every subgroup that meets the symptom definition and obtains SARS-CoV-2 testing has some probability of testing SARS-CoV-2 positive and some probability of testing SARS-CoV-2 negative. While the COVID-19 pandemic setting and seasonality affected circulation of other respiratory pathogens, there was still some non-zero probability for each subgroup to acquire SARS-CoV-2 infection or other respiratory pathogens that induce similar symptoms. When implementing future TND studies, care should be taken to enroll participants during time frames and in regions in which SARS-CoV-2 and other respiratory pathogens are circulating.

The second assumption requires every subgroup that meets the symptom definition, has either SARS-CoV-2 test result, and obtains SARS-CoV-2 testing to have some probability of observing their vaccination status. This assumption likely holds in TND studies.

The third assumption requires that for a given SARS-CoV-2 test result (if they were to seek testing), every subgroup that meets the symptom definition has some probability of obtaining a SARS-CoV-2 test. TND studies must be implemented during time frames and in regions in which SARS-CoV-2 testing is available, so this assumption restricts the source population to individuals who have access to testing, which likely includes healthcare-seeking individuals.

© 2025 Andrews LIB et al. *JAMA Network Open*.

The fourth assumption requires that vaccinated and unvaccinated healthcare-seeking individuals who meet the symptom definition and share other characteristics have the same probability of obtaining a SARS-CoV-2 test. Vaccination may reduce the probability of infection and symptom severity[26,27] and decrease symptom-triggered testing. In contrast, individuals at higher risk of COVID-19 or who are more health conscious may be more likely to obtain COVID-19 vaccination and/or SARS-CoV-2 testing. By conditioning on meeting the symptom definition and only generalizing to a healthcare-seeking population, this assumption is reasonable. While this assumption holds in our TND setting with blinded and randomized vaccination, observational TND studies should collect and adjust for additional covariates, such as comorbidities, infection history, and behavioral variables correlated with seeking SARS-CoV-2 testing, to help this assumption hold. Additionally, studying a severe COVID-19 endpoint could allow inferences to a broader population.

The fifth assumption states that missing vaccination status is conditionally independent of vaccination status, given meeting the symptom definition, obtaining SARS-CoV-2 testing, SARS-CoV-2 test result, and covariates. Vaccination status could be missing because individuals do not remember, are missing vaccination records, or decline to answer. Since vaccination was blindly administered and well-documented in the RCTs, this assumption holds in our TND study. In observational TND studies, additional covariates that could be related to vaccination status missingness, such as region and age, can be adjusted for to enhance plausibility of this assumption. Obtaining vaccination status from vaccine registries only may violate this assumption because unvaccinated individuals are more likely to be missing than vaccinated individuals.

The sixth and seventh assumptions assume that the data collected on SARS-CoV-2 testing and vaccination accurately reflect SARS-CoV-2 infection and true vaccination status. To ensure high SARS-CoV-2 test sensitivity and specificity, our study only considered test results from SARS-CoV-2 PCR and NAAT tests that occur within 10 days after symptom onset, following World Health Organization recommendations.[28,29] Additionally, some simulation studies have concluded that high test specificity is more important than sensitivity to limit bias in TND settings.[30,31] While we know which participants received the intervention they were randomized to in our TND study, observational studies rely on self-report or vaccine records, which are subject to vaccine misclassification.[32-35] However, since vaccine information is collected before SARS-CoV-2 status is known, TND studies are less subject to differential vaccine misclassification, which causes greater bias than nondifferential vaccine misclassification.[24,33]

The eighth assumption is Noncase Exchangeability, which states that the probability of meeting the symptom definition and testing SARS-CoV-2 negative is the same in vaccinated and unvaccinated individuals in a healthcare-seeking population who have the same characteristics that are adjusted for in a TND analysis.[22,23,36,37] This assumption could be violated if the COVID-19 vaccine affects non-COVID-19 illness in healthcare-seeking individuals with the same covariates. In our study, we formally assessed this assumption for each vaccine in the overall RCT cohort and for two age groups. We informally assessed this assumption for calendar date and geographic region by comparing across the diverse trial cohorts (Figure 3, eFigure 11 in Supplement 1). Since RCT protocols instructed participants to frequently report symptoms and obtain SARS-CoV-2 testing, we assumed RCT participants would have identical healthcare-seeking behavior. For the vaccines studied, Noncase Exchangeability seems reasonable, though additional subgroups could be investigated since the assumption depends on the covariates adjusted for in a TND analysis. Noncase Exchangeability could be violated if characteristics associated with COVID-19 vaccination and non-COVID-19 illness are not adjusted for, which we could not assess in our study. Thus, observational TND studies without randomized vaccination should measure and adjust for variables, such as influenza vaccination status, that may violate Noncase Exchangeability.[38]

Our approach also requires that the observed TND data $O = (O_1, \ldots, O_n)$ consist of independent and identically distributed individuals. However, some common TND sampling methods involve multiple SARS-CoV-2 tests per participant, which violates this independence assumption. Thus, the reported standard error estimates involving these sampling methods are likely smaller than if all SARS-CoV-2 tests were from independent participants. While the semiparametric logistic regression (and the ordinary logistic regression) provides inaccurate variance estimates for dependent data, our study's goal was to assess how well this statistical approach can handle common TND sampling methods. Future work may investigate how applying the semiparametric logistic regression to dependent data affects its finite and asymptotic properties.

© 2025 Andrews LIB et al. *JAMA Network Open*.

Under Bayes Rule and identifying assumptions 1-7, we can estimate $OR(P)(x)$ using our observed TND data and interpret it as $OR_F(P_F)(x)$, the odds ratio of virologically confirmed, symptomatic COVID-19 comparing symptomatic vaccinated and unvaccinated individuals in a healthcare-seeking population who have the same characteristics:

$$OR(P)(x) = \frac{P(A=1|Y=1,X=x,C=1,R=1,\Delta=1)/P(A=0|Y=1,X=x,C=1,R=1,\Delta=1)}{P(A=1|Y=0,X=x,C=1,R=1,\Delta=1)/P(A=0|Y=0,X=x,C=1,R=1,\Delta=1)}$$
$$= \frac{P(Y=1,C=1|A=1,X=x,R=1,\Delta=1)/P(Y=0,C=1|A=1,X=x,R=1,\Delta=1)}{P(Y=1,C=1|A=0,X=x,\ R=1,\Delta=1)/P(Y=0,C=1|A=0,X=x,R=1,\Delta=1)}$$
$$= \frac{P_F(Y=1,C=1|A=1,X=x)/P_F(Y=0,C=1|A=1,X=x)}{P_F(Y=1,C=1|A=0,X=x)/P_F(Y=0,C=1|A=0,X=x)}$$
$$= OR_F(P_F)(x)$$

To obtain the interpretable full data conditional risk ratio of COVID-19 comparing vaccinated to unvaccinated healthcare-seeking individuals who have the same characteristics, we apply Noncase Exchangeability:[22,23,39,40]

$$OR_F(P_F)(x) = \frac{P_F(Y=1,C=1|A=1,X=x)/P_F(Y=0,C=1|A=1,X=x)}{P_F(Y=1,C=1|A=0,X=x)/P_F(Y=0,C=1|A=0,X=x)}$$
$$= \frac{P_F(Y=1,C=1|A=1,X=x)}{P_F(Y=1,C=1|A=0,X=x)}$$
$$= RR_F(P_F)(x).$$

This identification result holds regardless of how the odds ratio is estimated; thus, Noncase Exchangeability is necessary in typical TND analyses involving ordinary logistic regression.

Causal Assumptions

With standard causal assumptions, we can identify the full data causal conditional risk ratio $RR_F(P_F)(x)$.[18,22] The symbol $=^d$ denotes that two distributions are identical.

9. $\{Y(a), C(a)|A=a\} =^d \{Y, C|A=a\}$ for $a=\{0,1\}$ (Consistency)
10. $Y_i(j),\ C_i(j) \perp\!\!\!\perp A_k$ for $a=\{0,1\}$ and all pairs of individuals $i \neq k$ (No Interference)
11. $Y(a), C(a) \perp\!\!\!\perp A|X$ for $a=\{0,1\}$ (No Unmeasured Confounding)

The ninth assumption is the consistency assumption, which assumes that hypothetical assignment to vaccination status $A=a$ results in the same SARS-CoV-2 test result (if they obtained testing) and symptom status as when the observed vaccination status is $A=a$. This is a standard assumption in causal inference[41,42] that requires no variability in vaccination statuses that may affect the potential outcomes. This assumption should be reasonable if the observed vaccination status represents a single version of treatment (e.g., everyone receives all planned vaccine doses of the same vaccine). Since our TND study restricts to individuals who received the intended number of doses and analyzes each trial cohort separately (Supplemental Table 1), this assumption is reasonable. Observational TND studies should carefully define vaccination status and the population of interest or adjust for additional covariates regarding individuals' vaccine history.

The tenth assumption is the no interference assumption,[39,43] which requires that the potential outcomes of each individual do not depend on other individuals' vaccination statuses. Since COVID-19 is transmissible through person-person contact and vaccination may reduce individuals' infectiousness,[26,27] the status of being vaccinated could reduce nearby individuals' risk of infection. This violates the assumption and could make the causal parameter poorly defined. However, bias from violating this assumption is mitigated since the RCTs were broadly geographically dispersed with many study sites and somewhat preceded public health vaccine campaigns, such that most individuals in the communities were unvaccinated. In the future, additional strategies could be implemented to account for this interference,[22,44] where one simple idea allows only one participant per household to enroll into the TND study.

The eleventh assumption requires that all covariates that are associated with vaccination status and affect meeting the symptom definition and/or SARS-CoV-2 test results are measured and fully accounted for. While our TND study does not have confounding because vaccination was blinded and randomized, in an observational TND study,

© 2025 Andrews LIB et al. *JAMA Network Open*.

biological characteristics like age, infection history, immune system effectiveness, and comorbidities, may influence individuals' decisions regarding vaccination and affect symptom presentation. Exposure characteristics, such as occupation, calendar date, community behavior, and geographical region, may affect individuals' probabilities of vaccination and SARS-CoV-2 infection. Thus, our study adjusts for covariates to mimic typical TND analysis strategies to meet this assumption.

Under these three causal assumptions, the full data conditional risk ratio $RR_F(P_F)(x)$ can be interpreted as the full data causal conditional risk ratio $RR_{F,ca}(P_{F,ca})(x)$.

Partially Linear Logistic Regression Model

Now that we have illustrated how the observed TND data conditional odds ratio $OR(P)(x)$ can identify the full data causal conditional risk ratio $RR_{F,ca}(P_{F,ca})(x)$, next we demonstrate how to estimate $OR(P)(x)$. First, we impose a partially linear logistic regression model onto the outcome regression function, $P_F(A = 1|Y = 1, X = x, C = 1)$,[45]

$$\text{logit}\, P_F(A = 1|Y = y, X = x, C = 1) = y\beta_F^T f(x) + h_{P_F}(x),$$

for an unknown vector of coefficients β_F of length s, a known vector-valued function $f: \mathbb{R}^d \to \mathbb{R}^s$, and unspecified $h_{P_F}(x)$= logit $P_F(A = 1|Y = 0, X = x, C = 1)$. From this semiparametric assumption on P_F,
we maintain an interpretable relationship between vaccination status and SARS-CoV-2 test result, $\log OR_F(P_F)(x) = \beta_F^T f(x)$, that can nonparametrically adjust for covariates using flexible, machine-learning methods to minimize bias from model misspecification.[46,47] This model can also specify effect modification via $f(x)$. If we believe the association between vaccination and COVID-19 is the same for all subgroups, then we let $f(x) = \langle 1 \rangle$, such that $\log OR_F(P_F)(x) = \beta_F$. Alternatively, if we believe vaccine effectiveness differs by sex and age, we could set $f(x) = \langle 1, male, age \rangle$, or some other function of those covariates, to allow for effect modification amongst subgroups. Then, we obtain vaccine effectiveness for each subgroup using $\log OR_F(P_F)(x) = \beta_{F,0} + \beta_{F,1} male + \beta_{F,2} age$.

We assume the observed TND data distribution satisfies the partially linear logistic regression model constraint and estimate $\log OR(P)(x)$ using the efficient influence function of the coefficient vector from a partially linear logistic regression model of vaccination status A on SARS-CoV-2 test result Y and covariates X using TND study sample individuals with complete vaccination information ($C = R = \Delta = 1$). We can use any machine-learning algorithms that can meet the model constraint, including highly adaptive lasso,[48] generalized additive models,[49] and smoothing splines.[50] For our study, we used the highly adaptive lasso because of its flexibility and fast convergence rate.

Targeted Maximum Likelihood Estimation

The estimator $\hat{\beta}(P_n)$ from the observed TND data is biased up to second order terms, so we use targeted maximum likelihood estimation (TMLE) to update and debias our estimator. We used the TMLE framework[46,51] rather than one-step estimation[52] or estimating equation approaches[53-55] to construct substitution estimators that respect model constraints and may have better finite sample properties.[56] The resulting TMLEs, $\hat{\beta}(P_n^*)$ and $\widehat{OR}(P_n^*)(x)$, are efficient, asymptotically normal estimators that are valid for inference under regularity conditions. Theoretical details regarding the efficient influence functions, variances, and conditions for inference are described in van der Laan and Gilbert (2023).[18] Code and toy datasets to implement this method in R version 4.2.2[57] using causalglm[58] and hal9001[59,60] R packages are on GitHub at https://github.com/leahandrews/eval-tnd-w-rcts. To our knowledge, this is the first application of semiparametric robust targeted maximum likelihood estimation/inference for assessing TND vaccine effectiveness.

Data Collection on Race and Ethnicity

Race and ethnicity were collected by self-report. Race was categorized as American Indian or Alaska Native, Asian, Black or African American, Native Hawaiian or other Pacific Islander, White, other, and multiple. Indigenous people from South America were classified together with the American Indian or Alaska Native US and Mexico demographic according to the US Food and Drug definition (i.e., a person having origins in any of the original peoples of North and South America [including Central America] and who maintains tribal affiliation or community

© 2025 Andrews LIB et al. *JAMA Network Open*.

attachment). Participants also had the option to select other race without specifying. Ethnicity was categorized as Hispanic or Latino or not Hispanic or Latino.

To mimic other TND analyses, which may adjust for race and ethnicity as a potential confounder depending on the region being studied,[61-63] we planned to adjust for race and ethnicity in all trial cohorts' TND analyses. Since each CoVPN trial enrolled participants from different countries and continents, we defined a binary race and ethnicity indicator (People of Color vs. Non-Hispanic/Latino White) to simplify the covariate adjustment and manage small subgroup sizes when also adjusting for geographic region and two-week testing date intervals (eTable 2 in Supplement 1). We defined People of Color as participants who reported their race as American Indian or Alaska Native, Asian, Black or African American, Multiple, Native Hawaiian or Other Pacific Islander, or other, and/or who reported their ethnicity as Hispanic or Latino. Non-Hispanic/Latino White were participants who reported their race as White and reported their ethnicity as Not Hispanic or Latino or did not report their ethnicity. Participants who did not report their race and either did not report their ethnicity or reported their ethnicity as Not Hispanic or Latino were classified as missing. Though we planned to adjust for race and ethnicity in all trial cohorts, in our final analyses, we only adjusted for race and ethnicity in Moderna COVE BN and the three Janssen ENSEMBLE BN trial cohorts because they had racial/ethnic heterogeneity within geographic regions, but the other trial cohorts did not.

© 2025 Andrews LIB et al. *JAMA Network Open*.

eReferences

1. Mena Lora AJ, Long JE, Huang Y, et al. Rapid Development of an Integrated Network Infrastructure to Conduct Phase 3 COVID-19 Vaccine Trials. *JAMA Netw Open.* 2023;6(1):e2251974.
2. El Sahly HM, Baden LR, Essink B, et al. Efficacy of the mRNA-1273 SARS-CoV-2 Vaccine at Completion of Blinded Phase. *N Engl J Med.* 2021;385(19):1774-1785.
3. Sobieszczyk ME, Maaske J, Falsey AR, et al. Durability of protection and immunogenicity of AZD1222 (ChAdOx1 nCoV-19) COVID-19 vaccine over 6 months. *J Clin Invest.* 2022;132(18).
4. Sadoff J, Gray G, Vandebosch A, et al. Final Analysis of Efficacy and Safety of Single-Dose Ad26.COV2.S. *N Engl J Med.* 2022;386(9):847-860.
5. Dunkle LM, Kotloff KL, Gay CL, et al. Efficacy and Safety of NVX-CoV2373 in Adults in the United States and Mexico. *N Engl J Med.* 2022;386(6):531-543.
6. Dayan GH, Rouphael N, Walsh SR, et al. Efficacy of a monovalent (D614) SARS-CoV-2 recombinant protein vaccine with AS03 adjuvant in adults: a phase 3, multi-country study. *EClinicalMedicine.* 2023;64:102168.
7. Dayan GH, Rouphael N, Walsh SR, et al. Efficacy of a bivalent (D614 + B.1.351) SARS-CoV-2 recombinant protein vaccine with AS03 adjuvant in adults: a phase 3, parallel, randomised, modified double-blind, placebo-controlled trial. *Lancet Respir Med.* 2023;11(11):975-990.
8. Efron B. The efficiency of Cox's likelihood function for censored data. *Journal of the American Statistical Association.* 1977;72(359):557-565.
9. Zou G. A modified poisson regression approach to prospective studies with binary data. *Am J Epidemiol.* 2004;159(7):702-706.
10. Nauta J. Statistics in Clinical Vaccine Trials. Springer Berlin, Heidelberg. 153 pages. ISBN: 978-3-642-44191-2. 2011.
11. Breslow N, Day N. Statistical methods in cancer research. Volume II--The design and analysis of cohort studies. IARC Sci Publ. 1987;(82):1–406.
12. United States Census Bureau. Guidance for Economic Census Geographies Users: Geographic Levels. Page last revised 8 October, 2021. Access date 15 November, 2024. Available from: https://www.census.gov/programs-surveys/economic-census/guidance-geographies/levels.html.
13. Lin LI. A concordance correlation coefficient to evaluate reproducibility. *Biometrics.* 1989;45(1):255-268.
14. COVID-Forecasting Team. Past SARS-CoV-2 infection protection against re-infection: a systematic review and meta-analysis. *Lancet.* 2023;401(10379):833-842.
15. Xiao AT, Tong YX, Zhang S. Profile of RT-PCR for SARS-CoV-2: A Preliminary Study From 56 COVID-19 Patients. *Clin Infect Dis.* 2020;71(16):2249-2251.
16. Wajnberg A, Mansour M, Leven E, et al. Humoral response and PCR positivity in patients with COVID-19 in the New York City region, USA: an observational study. *Lancet Microbe.* 2020;1(7):e283-e289.
17. Rhee C, Kanjilal S, Baker M, Klompas M. Duration of Severe Acute Respiratory Syndrome Coronavirus 2 (SARS-CoV-2) Infectivity: When Is It Safe to Discontinue Isolation? *Clin Infect Dis.* 2021;72(8):1467-1474.
18. van der Laan L, Gilbert PB. Semiparametric inference for relative heterogeneous vaccine efficacy between strains in observational case-only studies. arXiv:2303.11462 [stat.ME] [Preprint] Submitted 20 Mar 2023. Access date 10 Oct 2024. *arXiv.* 2023.
19. Follmann DA, Dodd L. Immune correlates analysis using vaccinees from test negative designs. *Biostatistics.* 2022;23(2):507-521.
20. Rubin DB. Inference and missing data. *Biometrika.* 1976;63(3):581-592.
21. Westreich D. Berkson's bias, selection bias, and missing data. *Epidemiology.* 2012;23(1):159-164.
22. Schnitzer ME. Estimands and Estimation of COVID-19 Vaccine Effectiveness Under the Test-Negative Design: Connections to Causal Inference. *Epidemiology.* 2022;33(3):325-333.
23. Jackson ML, Nelson JC. The test-negative design for estimating influenza vaccine effectiveness. *Vaccine.* 2013;31(17):2165-2168.
24. Sullivan SG, Tchetgen Tchetgen EJ, Cowling BJ. Theoretical Basis of the Test-Negative Study Design for Assessment of Influenza Vaccine Effectiveness. *Am J Epidemiol.* 2016;184(5):345-353.
25. Lewnard JA, Patel MM, Jewell NP, et al. Theoretical Framework for Retrospective Studies of the Effectiveness of SARS-CoV-2 Vaccines. *Epidemiology.* 2021;32(4):508-517.
26. Mohammed I, Nauman A, Paul P, et al. The efficacy and effectiveness of the COVID-19 vaccines in reducing infection, severity, hospitalization, and mortality: a systematic review. *Hum Vaccin Immunother.* 2022;18(1):2027160.

© 2025 Andrews LIB et al. *JAMA Network Open*.

27. Centers for Disease Control and Prevention. Science Brief: COVID-19 Vaccines and Vaccination. CDC Archive: https://archive.cdc.gov/www_cdc_gov/coronavirus/2019-ncov/science/science-briefs/fully-vaccinated-people.html Last updated 15 September, 2021. Access date 15 November, 2024.
28. Kucirka LM, Lauer SA, Laeyendecker O, Boon D, Lessler J. Variation in False-Negative Rate of Reverse Transcriptase Polymerase Chain Reaction-Based SARS-CoV-2 Tests by Time Since Exposure. *Ann Intern Med.* 2020;173(4):262-267.
29. Patel MK, Bergeri I, Bresee JS, et al. Evaluation of post-introduction COVID-19 vaccine effectiveness: Summary of interim guidance of the World Health Organization. *Vaccine.* 2021;39(30):4013-4024.
30. Orenstein EW, De Serres G, Haber MJ, et al. Methodologic issues regarding the use of three observational study designs to assess influenza vaccine effectiveness. *Int J Epidemiol.* 2007;36(3):623-631.
31. Jackson ML, Rothman KJ. Effects of imperfect test sensitivity and specificity on observational studies of influenza vaccine effectiveness. *Vaccine.* 2015;33(11):1313-1316.
32. Jackson ML. Use of self-reported vaccination status can bias vaccine effectiveness estimates from test-negative studies. *Vaccine X.* 2019;1:100003.
33. Liang Y, Driscoll AJ, Patel PD, et al. Typhoid conjugate vaccine effectiveness in Malawi: evaluation of a test-negative design using randomised, controlled clinical trial data. *Lancet Glob Health.* 2023;11(1):e136-e144.
34. Rolnick S, Parker E, Nordin J, et al. Self-report compared to electronic medical record across eight adult vaccines: do results vary by demographic factors? *Vaccine.* 2013;31(37):3928-3935.
35. Zimmerman RK, Raymund M, Janosky JE, Nowalk MP, Fine MJ. Sensitivity and specificity of patient self-report of influenza and pneumococcal polysaccharide vaccinations among elderly outpatients in diverse patient care strata. *Vaccine.* 2003;21(13-14):1486-1491.
36. Broome CV, Facklam RR, Fraser DW. Pneumococcal disease after pneumococcal vaccination: an alternative method to estimate the efficacy of pneumococcal vaccine. *N Engl J Med.* 1980;303(10):549-552.
37. Clemens JD, Shapiro ED. Resolving the pneumococcal vaccine controversy: are there alternatives to randomized clinical trials? *Rev Infect Dis.* 1984;6(5):589-600.
38. Doll MK, Pettigrew SM, Ma J, Verma A. Effects of Confounding Bias in Coronavirus Disease 2019 (COVID-19) and Influenza Vaccine Effectiveness Test-Negative Designs Due to Correlated Influenza and COVID-19 Vaccination Behaviors. *Clin Infect Dis.* 2022;75(1):e564-e571.
39. Rubin DB. Randomization Analysis of Experimental Data: The Fisher Randomization Test Comment. *Journal of the American Statistical Association.* 1980;75(371):591-593.
40. Jiang C, Talbot D, Carazo S, Schnitzer ME. A Double Machine Learning Approach for the Evaluation of COVID-19 Vaccine Effectiveness Under the Test-Negative Design: Analysis of Quebec Administrative Data. *Stat Med.* 2025;44(5):e70025.
41. Pearl J. Causality. Cambridge University Press; 2009. 487 p. .
42. Rubin DB. Causal Inference Using Potential Outcomes. *Journal of the American Statistical Association.* 2005;100(469):322-331.
43. Cox DR. Planning of experiments. Oxford, England: Wiley; 1958. 308 p. (Planning of experiments).
44. Hudgens MG, Halloran ME. Toward Causal Inference With Interference. *J Am Stat Assoc.* 2008;103(482):832-842.
45. Tchetgen Tchetgen EJ, Robins JM, Rotnitzky A. On doubly robust estimation in a semiparametric odds ratio model. *Biometrika.* 2010;97(1):171-180.
46. van der Laan MJ, Rose S. *Targeted Learning: Causal Inference for Observational and Experimental Data.* Springer New York, NY; 2011.
47. van der Laan M. CV-TMLE and double machine learning. 24 December, 2019. Access date 11 October, 2024. https://vanderlaan-lab.org/2019/12/24/cv-tmle-and-double-machine-learning/. Accessed.
48. Benkeser D, van der Laan M. The Highly Adaptive Lasso Estimator. *Proc Int Conf Data Sci Adv Anal.* 2016;2016:689-696.
49. Hastie T, Tibshirani R. Generalized Additive Models: Some Applications. *Journal of the American Statistical Association.* 1987;82(398):371-386.
50. Friedman JH. Multivariate Adaptive Regression Splines. *The Annals of Statistics.* 1991;19(1):1-67, 67.
51. van der Laan MJ, Rubin D. Targeted Maximum Likelihood Learning. *The International Journal of Biostatistics.* 2006;2(1).
52. Bickel PJ. Efficient and adaptive estimation for semiparametric models. Baltimore: Johns Hopkins University Press; 1993. (Johns Hopkins series in the mathematical sciences).

© 2025 Andrews LIB et al. *JAMA Network Open*.

53. van der Laan MJ, Robins JM. Unified Methods for Censored Longitudinal Data and Causality. Part of the Springer Series in Statistics. Springer New York, NY. 399 pages. ISBN: 978-0-387-95556-8. 2003.
54. Chernozhukov V, Chetverikov D, Demirer M, et al. Double/debiased machine learning for treatment and structural parameters. *The Econometrics Journal.* 2018;21(1):C1-C68.
55. Robins JM, Rotnitzky A, Zhao LP. Estimation of Regression Coefficients When Some Regressors Are Not Always Observed. *Journal of the American Statistical Association.* 1994;89(427):846-866.
56. Porter KE, Gruber S, Laan MJvd, Sekhon JS. The Relative Performance of Targeted Maximum Likelihood Estimators. *The International Journal of Biostatistics.* 2011;7(1).
57. R Core Team. R: A language and environment for statistical computing. R Foundation for Statistical Computing, Vienna, Austria. https://www.R-project.org/. 2024.
58. van der Laan L. causalglm: Interpretable and robust causal inference for heterogeneous treatment effects using generalized linear models with targeted machine-learning. https://github.com/tlverse/causalglm. Cited 15 November, 2024.
59. Hejazi NS, Coyle JR, van der Laan MJ. hal9001: Scalable highly adaptive lasso regression in R. *Journal of Open Source Software.* 2020;5(53):2526.
60. Hejazi NS, Coyle JR, van der Laan MJ. hal9001: Scalable highly adaptive lasso regression in R. Cited 10 October 2024. Zenodo. DOI: 10.5281/zenodo.3558313. 2020.
61. Tenforde MW, Olson SM, Self WH, et al. Effectiveness of Pfizer-BioNTech and Moderna Vaccines Against COVID-19 Among Hospitalized Adults Aged >/=65 Years - United States, January-March 2021. *MMWR Morb Mortal Wkly Rep.* 2021;70(18):674-679.
62. Olson SM, Newhams MM, Halasa NB, et al. Effectiveness of Pfizer-BioNTech mRNA Vaccination Against COVID-19 Hospitalization Among Persons Aged 12-18 Years - United States, June-September 2021. *MMWR Morb Mortal Wkly Rep.* 2021;70(42):1483-1488.
63. Lopez Bernal J, Andrews N, Gower C, et al. Effectiveness of the Pfizer-BioNTech and Oxford-AstraZeneca vaccines on covid-19 related symptoms, hospital admissions, and mortality in older adults in England: test negative case-control study. *BMJ.* 2021;373:n1088.

© 2025 Andrews LIB et al. *JAMA Network Open*.
