# Supplementary material for: Evaluating the Test-Negative Design for COVID-19 Vaccine Effectiveness Using Randomized Trial Data: A Secondary Cross-Protocol Analysis of 5 Randomized Clinical Trials
Source: JAMA Netw Open. 2025 May 28;8(5):e2512763. doi: 10.1001/jamanetworkopen.2025.12763 (PMC12120655; doi:10.1001/jamanetworkopen.2025.12763)
Supplement: Supplement 2. — Nonauthor Collaborators. The COVID-19 Prevention Network (CoVPN) [file jamanetwopen-e2512763-s002.pdf]

Supplemental Online Content: Nonauthor Collaborators
*First name, last name, and suffix (if applicable) are required and will appear in PubMed.

***Group Name(s): COVID-19 Prevention Network (CoVPN)**

***First Name and Middle Initial(s)**	***Last Name**	***Suffix (eg, Jr, III)**	Academic Degrees	Institution	Location (city, state/province, country)	Role or Contribution, eg, chair, principal investigator	Group (if more than 1 Group listed in the byline) and/or Subgroup (eg, Steering Committee)
Vanessa	Abad		NP				
Karina	Abalos						
Getahun	Abate		MD, PhD, MSc				
Anum	Abbas		MD				
Jose	Accini						
Ronald	Ackerman		MD				
Jamie	Ackerman						
Jeremy	Ackermann						
Atoya	Adams		MD, MBA				
Michael R.	Adams		MD				
Mark S.	Adams						
Michael	Adams						
Jeffrey M.	Adelglass		MD				
Sarah	Afework						
Nina	Ahmad		MD				
Christine	Akamine		MD				
Julie A.	Ake		MD, MSc				
Gary	Albert						
Maria L.	Alcaide						
Nathan	Alderson		PhD				
Lily	Aleman						
José O.	Alemán						
Mohamed S.	Al-Ibrahim						
Mohammed	Allaw		MD				
Leland N.	Allen	III	MD				
Mary	Allen		MS				
Naveena	Aloysia						
Hilario	Alvarado		MD				

Supplemental Online Content: Nonauthor Collaborators
*First name, last name, and suffix (if applicable) are required and will appear in PubMed.

*First Name and Middle Initial(s)	*Last Name	*Suffix (eg, Jr, III)	Academic Degrees	Institution	Location (city, state/province, country)	Role or Contribution, eg, chair, principal investigator	Group (if more than 1 Group listed in the byline) and/or Subgroup (eg, Steering Committee)
Winniberg Stephany	Alvarez León						
Carmen	Amador		ARNP				
John Humphrey	Amuasi						
James	Andersen		MD				
Evan J.	Anderson		MD				
Corey G.	Anderson		MD				
Victoria R.	Anderson		MS				
David R.	Andes						
Michele P.	Andrasik		PhD				
Stephanie	Andree		FNP				
Jeb	Andrews						
Jessica G.	Andriesen		PhD				
Victoria	Andriulis						
Germán	Áñez		MD				
Maria	Angeles Ceregido		PhD				
Nana Akosua	Ansah						
Jessica L.	Ansel		MSN				
Codi M.	Anthony		DNP, APRN, PMHNP-BC				
Johannes	Antony		PhD				
Mohammed	Antwi						
Roberto C.	Arduino						
Florida	Aristy		APRN				
Valerie K.	Arnold		MD				
Alicia	Arrazcaeta						
Hugo Macareno	Arroyo						
Kwaku Poku	Asante		PhD				

*First name, last name, and suffix (if applicable) are required and will appear in PubMed.

***First Name and Middle Initial(s)**	***Last Name**	***Suffix (eg, Jr, III)**	Academic Degrees	Institution	Location (city, state/province, country)	Role or Contribution, eg, chair, principal investigator	Group (if more than 1 Group listed in the byline) and/or Subgroup (eg, Steering Committee)
Robert L.	Atmar		MD				
Allison	August		MD				
Ayoade	Avworo		DNP				
Christina	Bacher		PAC				
Martín	Bäcker						
Lindsay R.	Baden		MD				
Diana	Badillo						
Emma	Bainbridge						
Michele	Baka		MD				
Sherrie	Baker		BS				
Bindu	Balani		MD				
Brandy	Ball						
Mira	Baron		MD				
Dan H.	Barouch		MD				
Elizabeth	Barranco-Santana		MD				
Alejandro Quintín	Barrat Hernández		MD				
Darlene	Bartilucci		MD				
Jennifer	Bashour		MD				
Teresa A.	Batteiger						
Clarisse	Baudelaire						
George H.	Bauer	Jr.	MD				
Jose A.	Bazan						
Maria G.	Becerra		MD				
Cynthia	Becher Strout		MD				
Teresa	Becker		MD				
David	Beckmann		MD				
Roger J.	Bedimo						
Linda R.	Belhorn		MD				
Genevieve	Beninati		FNP				

Supplemental Online Content: Nonauthor Collaborators
*First name, last name, and suffix (if applicable) are required and will appear in PubMed.

***First Name and Middle Initial(s)**	***Last Name**	***Suffix (eg, Jr, III)**	Academic Degrees	Institution	Location (city, state/province, country)	Role or Contribution, eg, chair, principal investigator	Group (if more than 1 Group listed in the byline) and/or Subgroup (eg, Steering Committee)
Jorge A.	Benitez						
David	Benkeser		PhD				
Annette R.	Bennett						
Elizabeth	Bennett		PA				
Tatiana	Beresnev		MD				
Aude	Berge						
Megan	Berman		MD				
Maria I.	Bermudez		MD				
Marcia	Bernard		NP				
David I.	Bernstein						
Jennifer	Berry		FNP-BC				
Andrea	Berry		MD				
Vladimir	Berthaud		MD, MPH				
Hanna	Beyko						
Kristin	Bialobok						
Joanne L.	Billings		MD, MPH				
Oleksandra	Bilotkach						
Zachary	Bittner						
Jeanne	Blevins						
Jill	Blumenthal		MD				
Rebecca	Boas						
Joseph	Bocchini		MD				
Tyler D.	Bold		MD, PhD				
Matthew I.	Bonaparte		PhD				
Alison	Bondell						
Paul	Bondy		MD				
Alberto Cadena	Bonfanti						
Judith	Borger		DO				
Scott	Borgetti						
Joseph	Boscia						
Dean M.	Boswell		BS				

Supplemental Online Content: Nonauthor Collaborators
*First name, last name, and suffix (if applicable) are required and will appear in PubMed.

***First Name and Middle Initial(s)**	***Last Name**	***Suffix (eg, Jr, III)**	Academic Degrees	Institution	Location (city, state/province, country)	Role or Contribution, eg, chair, principal investigator	Group (if more than 1 Group listed in the byline) and/or Subgroup (eg, Steering Committee)
Tana	Bourgeois		RN				
Maggie	Bowers		PA-C				
Paul S	Bradley		MD				
Judith	Brady						
Riemke	Brakema		MD				
Andres	Bran		MD				
Angela R.	Branche		MD				
Boerries	Brandenburg		PhD				
Donald M.	Brandon		MD				
Eric	Bravo		MD				
Thomas	Breuer						
Margaret	Brewinski Isaacs		MD, MPH				
Liz	Briesemeister						
Toby	Briskin		MD				
Gail	Broder						
Adam B.	Brosz		MD				
David	Browder		MD				
Lesley	Browder		MD				
Jeremy	Brown		DO				
Cynthia	Brown						
Robert	Brownlee						
Elizabeth	Bruce		MD				
Susan	Buchbinder		MD				
Cynthia	Bueno		PA-C				
Elisabeth	Bukusi						
Delia	Bullock		MD				
Catherine A.	Bunce						
Cortney	Burch						
Michelle L.	Burgett		RN				
Abram	Burgher		MD				

Supplemental Online Content: Nonauthor Collaborators
*First name, last name, and suffix (if applicable) are required and will appear in PubMed.

***First Name and Middle Initial(s)**	***Last Name**	***Suffix (eg, Jr, III)**	Academic Degrees	Institution	Location (city, state/province, country)	Role or Contribution, eg, chair, principal investigator	Group (if more than 1 Group listed in the byline) and/or Subgroup (eg, Steering Committee)
Larry	Bush		MD				
Michael B.	Butcher		MD				
Robert J.	Buynak		MD				
LaShondra	Cade						
Rafael	Calderon		MD				
Richard	Calderone		MD				
Amy	Caldwell		RN				
Cornell	Calinescu		MD				
Robert S.	Call						
Jim	Callis		PA-C				
Laura Julia	Camacho Choza						
Andres	Camacho-Gonzalez		MD				
Thomas B.	Campbell		MD				
Wesley	Campbell						
Michael	Cancilla		NP				
Michael	Cancilla		PA				
Kevin D.	Cannon		MD				
Karina	Cano Martínez		MD				
Richard	Canter						
Valeria D.	Cantos		MD				
Vicky	Cárdenas		PhD				
Martha	Carmen Medina		PA-C				
Ellie	Carmody						
Christopher	Carpenter						
Melissa	Carr		BA				
Monica B.	Carrascal						
Jaime Augusto	Carrillo						
Steven E.	Carsons						

Supplemental Online Content: Nonauthor Collaborators
*First name, last name, and suffix (if applicable) are required and will appear in PubMed.

*First Name and Middle Initial(s)	*Last Name	*Suffix (eg, Jr, III)	Academic Degrees	Institution	Location (city, state/province, country)	Role or Contribution, eg, chair, principal investigator	Group (if more than 1 Group listed in the byline) and/or Subgroup (eg, Steering Committee)
Marianela	Carvajal		APRN				
Denise	Casey		RN				
Jorge	Caso		MD, CPI				
Flora	Castellino		MD				
Marvin	Castellon						
Jose	Castillo Mancilla		MD				
Mario	Castro						
Hannah	Catan						
Nancy	Cauwenberghs		PhD				
Winston	Cavert		MD				
Médéric	Celle		MD				
Jorge A.	Chacón		MD				
Mesha M.	Chadwick		MD				
Amy	Chamberlain						
Austin	Chan		MD				
Jennifer	Chang						
Danaya	Chansinghakul						
Mouna G.	Chebib						
Corey M.	Chen						
Aiying	Chen		PhD				
Margaret	Cheng						
Roman M.	Chicz		PhD				
Iksung	Cho		MS				
Karin	Choquette		MSN, ABNP-C				
R. Michelle	Chouteau		MD				
Brian D. W.	Chow						
Lisa	Chrisley						

*First name, last name, and suffix (if applicable) are required and will appear in PubMed.

***First Name and Middle Initial(s)**	***Last Name**	***Suffix (eg, Jr, III)**	Academic Degrees	Institution	Location (city, state/province, country)	Role or Contribution, eg, chair, principal investigator	Group (if more than 1 Group listed in the byline) and/or Subgroup (eg, Steering Committee)
Shane G.	Christensen		MD				
Laurence	Chu		MD				
Hanh	Chu						
Hanh	Chu						
Waseem	Chughtai		BS, MBBS				
Michael	Chung		MD				
Annie	Ciambruschini						
Cristina	Cicogna						
Alexander	Clark						
Jesse L.	Clark		MD				
Phillip	Claybrook		MD				
Andrea	Clement		PA				
Lauren	Clement		NP-C				
Steve	Clemons						
Heather	Clouting		MSc				
Lisa	Cohen		DO				
Stuart H.	Cohen		MD				
Jacob	Coleman						
Joseph P.	Connor						
James H.	Conway						
Erin	Cooksey		MD				
Robert W.	Coombs		MD, PhD				
Maureen	Cooney						
Deanna	Cooper						
Paul	Coplan		ScD, MS, MBA				
Lawrence	Corey		MD				
Raymond	Cornelison		MD				
Barbara	Corral		APRN				
Jordan	Coslet		NP				
Erin	Coston						

*First name, last name, and suffix (if applicable) are required and will appear in PubMed.

*First Name and Middle Initial(s)	*Last Name	*Suffix (eg, Jr, III)	Academic Degrees	Institution	Location (city, state/province, country)	Role or Contribution, eg, chair, principal investigator	Group (if more than 1 Group listed in the byline) and/or Subgroup (eg, Steering Committee)
Michael J.	Cotugno		MD				
Florence	Coux						
Jessica	Cowden		MD, MSPH				
Paul	Cramer		APRN				
Clarence B.	Creech	II	MD				
Charnell	Cromer		RN, MSN				
Marshall A.	Cross		MD				
Aurelio	Cruz-Valdez		PhD				
Marcel	Curlin						
LaKondria	Curry						
Jerome	Custers		PhD				
Erika A.	Cutz		MD				
Eric S.	Daar		MD				
Michelle	DallaPiazza		MD				
Dima	Dandachi		MD, MPH				
Elizabeth	Danford		MD				
Lyly	Dang		DNP-BC				
Stephen	Daniels		DO				
Chandan	Das						
Santa Kumar	Das						
Matthew	Davies						
Yvonne	Davis						
Joseph D.	Davis		MD				
William S.	Davis	Jr.	MS, PA-C				
William B.	Davis		MD				
Matthew G.	Davis		MD				
Rajesh K.	Davit		MD				
Gustavo H.	Dayan		MD				
Anne Marit	de Groot		PhD				
Luis I.	De La Cruz		MD				

Supplemental Online Content: Nonauthor Collaborators
*First name, last name, and suffix (if applicable) are required and will appear in PubMed.

***First Name and Middle Initial(s)**	***Last Name**	***Suffix (eg, Jr, III)**	Academic Degrees	Institution	Location (city, state/province, country)	Role or Contribution, eg, chair, principal investigator	Group (if more than 1 Group listed in the byline) and/or Subgroup (eg, Steering Committee)
Claudia	De La Matta Rodriguez						
Ian	De Proost		PhD				
Christopher	Dedon		APRN, FNP-C				
Jon F.	Dedon						
Teresa	Deese						
Emily	Degan						
Elliot	DeHaan		MD				
Edwin	DeJesus		MD				
Carlos	del Rio		MD				
Michael	Delgado		MD				
Mike	Delgado						
Ernesto	Delgado		ARNP				
David	Dellaero		MD				
Weiping	Deng		PhD				
Douglas S.	Denham		DO				
Chinar	Desai						
Parul	Desai		NP				
Michael	Desjardins		MD				
Karen	Deutsch		NP				
Michael E.	Dever		MD				
Naomi	Devine		NP-C				
Louis	Devlin						
Carlos A.	Diazgranados		MD				
Secia	Díaz-Miralrio						
Michelle	Dickey						
David J.	Diemert		MD				
Craig	Dietz						
Susanne	Doblecki-Lewis		MD				

Supplemental Online Content: Nonauthor Collaborators
*First name, last name, and suffix (if applicable) are required and will appear in PubMed.

***First Name and Middle Initial(s)**	***Last Name**	***Suffix (eg, Jr, III)**	Academic Degrees	Institution	Location (city, state/province, country)	Role or Contribution, eg, chair, principal investigator	Group (if more than 1 Group listed in the byline) and/or Subgroup (eg, Steering Committee)
Dmytro	Dobrianskyi		MD				
Jennifer L	Dong						
Tambra	Dora						
Brenda	Dorcely						
Macaya	Douoguih		MD				
Matthew W.	Doust		MD				
Frances	Downing						
Michael P.	Dube						
Filip	Dubovsky		MD, MPH				
Pete	Ducheneaux		LPN				
Jeffrey	Dugas	Sr.	MD				
Aeiress	Duhart						
Elizabeth	Duke		MD				
Debora	Dunbar						
Haoua	Dunbar						
Daniel M.	Duncanson		MD				
Lisa M.	Dunkle		MD				
Hillary	Dunlevy						
Anna	Durbin		MD				
Amy	Dye						
Carmel B.	Dyer						
Sarah	Dzigiel						
Mary	Easley		BSN, RN				
Niles	Eaton						
Valentine M.	Ebuh		MD, MA, MSc				
Georgette	Ebuh		DNP, APRN, FNP-C				
Lynn	Eckert		PA-C				
Benjamin	Eckhardt						

Supplemental Online Content: Nonauthor Collaborators
*First name, last name, and suffix (if applicable) are required and will appear in PubMed.

***First Name and Middle Initial(s)**	***Last Name**	***Suffix (eg, Jr, III)**	Academic Degrees	Institution	Location (city, state/province, country)	Role or Contribution, eg, chair, principal investigator	Group (if more than 1 Group listed in the byline) and/or Subgroup (eg, Steering Committee)
Frank S.	Eder		MD				
Srilatha	Edupuganti		MD				
Leslie A.	Edwards		MSN, CRNP				
Mark	Eickhoff						
Hana M.	El Sahly		MD				
Edward	Ellerbeck						
Grant	Ellsworth		MD, MS				
Elwaleed	Elnagar		MD				
Victoria	Engler		FNP				
Kendra	Enright		RN				
David J.	Ensz		MD				
Sally	Eppenbach		NP				
Sandra	Erickson						
Joseph	Eron	Jr.	MD				
Joseph J.	Eron		MD				
Tiffany	Esinhart		PA-C				
Claudia	Espinosa		MD				
Luis	Espinoza						
Brandon J.	Essink		MD				
Elizabeth	Everette						
Evan C.	Ewers						
Beverly	Ewing		APRN, DNP, FNP-BC				
Amy	Falk						
Ann R.	Falsey						
Norberto	Fas		MD				
Michael	Fay		PhD				
Szheckera	Fearon		MSN, FNP-C				

Supplemental Online Content: Nonauthor Collaborators
*First name, last name, and suffix (if applicable) are required and will appear in PubMed.

*First Name and Middle Initial(s)	*Last Name	*Suffix (eg, Jr, III)	Academic Degrees	Institution	Location (city, state/province, country)	Role or Contribution, eg, chair, principal investigator	Group (if more than 1 Group listed in the byline) and/or Subgroup (eg, Steering Committee)
Brittany	Feijoo						
Gregory J.	Feldman		MD				
Uriel R.	Felsen						
Hein	Fennema		PhD				
Carl J.	Fichtenbaum		MD				
Tom	Fiel						
Carlos A.	Fierro		MD				
Kenneth	Finkelstein		DO				
Jon	Finley		MD				
Suzanne	Fiorillo		MSPH				
David	Fitz-Patrick						
Diana F.	Florescu		MD				
Patrick	Flume		MD				
Charles M.	Fogarty						
Jennifer	Foley						
Dean	Follmann		PhD				
Pink	Folmar		MD				
Lyndsea	Folsom						
Youyi	Fong		PhD				
Stacy	Ford						
Lina M.	Forero						
Les B.	Forgosh		MD				
Elizabeth	Formentini						
Rhodna	Fouts						
Carina	Frago						
Veronica G.	Fragoso		MD				
Doris	Franco-Vitteri						
Ian	Frank		MD				
George H.	Freeman		MD				
Lena R.	Freese						
Robert W.	Frenck	Jr.					

Supplemental Online Content: Nonauthor Collaborators
*First name, last name, and suffix (if applicable) are required and will appear in PubMed.

***First Name and Middle Initial(s)**	***Last Name**	***Suffix (eg, Jr, III)**	Academic Degrees	Institution	Location (city, state/province, country)	Role or Contribution, eg, chair, principal investigator	Group (if more than 1 Group listed in the byline) and/or Subgroup (eg, Steering Committee)
Karen	Freudemann						
Sharon E.	Frey		MD				
David L.	Fried		MD				
Luke	Friesen		PA				
James	Fry		PA-C				
Bo	Fu		PhD				
Hiroyuki	Fukase						
Dawn	Furey		BA				
Christopher	Galloway		MD				
Sarah	Galloway		BA				
Edgar	Garcia		PA-C				
Jenina	Garcia						
Iris	García		BSN, RN				
Sebastian	Garcia Escallon		BA				
Veronica	Garcia-Fragoso		MD				
Agnes	Garinga						
Janet	Garvey		DNP				
Cynthia L.	Gay		MD				
Bruce C.	Gebhardt		MD				
Grant	Geiger						
Huub G.	Gelderblom		MD, PhD, MPH				
Georgettea	Geuss						
Elham	Ghadishah						
Elie	Gharib						
Suzanne	Gharib						
Richard L.	Gibson		MD				
Peter B.	Gilbert						

Supplemental Online Content: Nonauthor Collaborators
*First name, last name, and suffix (if applicable) are required and will appear in PubMed.

***First Name and Middle Initial(s)**	***Last Name**	***Suffix (eg, Jr, III)**	Academic Degrees	Institution	Location (city, state/province, country)	Role or Contribution, eg, chair, principal investigator	Group (if more than 1 Group listed in the byline) and/or Subgroup (eg, Steering Committee)
Alfredo	Gilberto Guerreros Benavides						
Teletha	Gipson		PhD, MS				
Eneyda	Giuvanela Llerena Zegarra						
Gregory M.	Glenn		MD				
Richard M.	Glover	II	MD				
Erin A.	Goecker		MS				
Paul A.	Goepfert		MD				
James N.	Goldenberg						
Blanca	Gomez		FNP-C				
Martha Cecilia	Gómora Madrid						
Jose	Gonzales-Zamora						
Celia	Gonzalez						
Lazaro	Gonzalez		APRN				
Viviana	Gonzalez						
Claudia	Gonzalez						
Luis H.	González						
Denisse Alejandra	González Estrada						
Antonio	Gonzalez-Lopez		MD, PhD, MPH				
Sarah	Gordon		MS				
Bryan	Gordon						
Richard	Gorman		MD				
Gregory M.	Gottschlich		MD				
Melissa	Gottschlich		PA-C				

Supplemental Online Content: Nonauthor Collaborators
*First name, last name, and suffix (if applicable) are required and will appear in PubMed.

*First Name and Middle Initial(s)	*Last Name	*Suffix (eg, Jr, III)	Academic Degrees	Institution	Location (city, state/province, country)	Role or Contribution, eg, chair, principal investigator	Group (if more than 1 Group listed in the byline) and/or Subgroup (eg, Steering Committee)
Shanda	Gower		APRN, CNP				
Daniel S.	Graciaa		MD				
Barney S.	Graham		MD				
Irene	Graham		MD				
Maria Angelica	Granados						
Amber	Grant		APRN				
Brett	Gray						
Glenda	Gray		MBBCh				
Roni	Gray		APRN				
Sinikka L.	Green		MD				
Allison	Green						
Justin A.	Green		MD, PhD				
Rusty	Greene						
Caroline	Greene		ANP-BC				
Cathy	Greiwe						
Alex	Greninger		MD, PhD, MS, Mphil				
Carl P.	Griffin		MD				
Marie-Helene	Grillet		MD				
Joseph	Grillo		MD				
Beatriz	Grinsztejn		MD, PhD				
Robert M.	Grossberg						
Nicole	Grunenberg		MD				
Bernard	Grunstra		MD				
Juan V.	Guanira-Carranza						
Sonia	Guerrero						
Lea	Guillery						
Clint C.	Guillory						

Supplemental Online Content: Nonauthor Collaborators
*First name, last name, and suffix (if applicable) are required and will appear in PubMed.

***First Name and Middle Initial(s)**	***Last Name**	***Suffix (eg, Jr, III)**	Academic Degrees	Institution	Location (city, state/province, country)	Role or Contribution, eg, chair, principal investigator	Group (if more than 1 Group listed in the byline) and/or Subgroup (eg, Steering Committee)
Caitlin J.	Guiney		MSN				
Catherine Q.	Gular		PharmD				
Shauna H.	Gunaratne						
Sanjay	Gurunathan		MD				
Milton	Haber		MD				
Greg	Hachigian		MD				
Amina Z.	Haggag		MD				
William	Hahn		MD				
Evelyn	Hall		MMS, PA-C				
Jessicalee	Hall						
David	Halpert						
Holli	Hamilton						
Elizabeth	Hammershaimb		MD, MS				
Shu	Han		PhD				
Laurie J.	Han-Conrad		MD				
Kate	Harden		PA-C				
Karin	Hardt		PhD				
Esther L.	Harmon		ANP				
Wayne L.	Harper		MD				
Charles	Harper		MD				
Nicole	Harrell		MD				
Hannah	Harrington		MPH				
Tavane	Harrison		CNP				
Kristina	Harrison						
Shana	Harshell						
William R.	Hartman						
Robyn	Hartvickson		MD				
Michael	Hassman		DO				
Timothy J.	Hatlen		MD				

Supplemental Online Content: Nonauthor Collaborators
*First name, last name, and suffix (if applicable) are required and will appear in PubMed.

***First Name and Middle Initial(s)**	***Last Name**	***Suffix (eg, Jr, III)**	Academic Degrees	Institution	Location (city, state/province, country)	Role or Contribution, eg, chair, principal investigator	Group (if more than 1 Group listed in the byline) and/or Subgroup (eg, Steering Committee)
Kirsten	Hauge		MPH				
Lynne A.	Haughey		MSN, FNP				
Michelle	Haynes						
Gary	Headden		MD				
C. Mary	Healy		MD				
Parke	Hedges		MD				
Dirk	Heerwegh		PhD				
Theresa	Hegmann		PA				
Jessica	Heimonen		MPH				
Destiny S.	Heinzig-Cartwright		BA				
Sheryl L.	Henderson						
Jeffrey A.	Henderson		MD, MPH				
Renea	Henderson		DO				
Jenny	Hendriks		PhD				
Gayle D.	Hennekes		PA-C, MPAS				
Matthew	Hepburn		MD				
Ramin	Herati						
Erica	Herc		MD				
Will	Hernandez		NP				
Laura	Hernandez Guarin						
Maria	Hernandez Moran		APRN				
Robin	Hilder						
John M.	Hill		MD				
Janet	Hill		PA-C				
Ian	Hirsch		PhD				
Ken	Ho						

Supplemental Online Content: Nonauthor Collaborators
*First name, last name, and suffix (if applicable) are required and will appear in PubMed.

***First Name and Middle Initial(s)**	***Last Name**	***Suffix (eg, Jr, III)**	Academic Degrees	Institution	Location (city, state/province, country)	Role or Contribution, eg, chair, principal investigator	Group (if more than 1 Group listed in the byline) and/or Subgroup (eg, Steering Committee)
Alejandro	Hoberman		MD				
Daniel	Hoft		MD, PhD				
Leila	Hojat						
Troy	Holdeman		MD				
Ripley R.	Hollister		MD				
Lisa	Holloway		MD				
Gregory	Holt		MD				
Matthew	Hong		MD				
Stephan	Hong		MD				
Matthew	Hong		MD				
Rosamond	Hong		MD				
Amanda	Horn		APRN				
Manisha	Horton						
Gary	Horwith		MD				
Sybil G.	Hosek						
Cyrus	Hoseyni		PhD				
Patty	Howenstine						
Yunda	Huang		PhD				
Ying	Huang						
Kathy	Hudzina						
Cory J.	Huffine		FNP-C				
Esther S.	Huffine		FNP-C				
Christine	Hull						
Julie	Hunt		PhD				
John	Hural						
James W.	Hurst		MD				
Shamika	Huskey		FNP				
Mary	Hussain		BS				
Julie	Hussey		MSN, APRN, FNP-C				

Supplemental Online Content: Nonauthor Collaborators
*First name, last name, and suffix (if applicable) are required and will appear in PubMed.

*First Name and Middle Initial(s)	*Last Name	*Suffix (eg, Jr, III)	Academic Degrees	Institution	Location (city, state/province, country)	Role or Contribution, eg, chair, principal investigator	Group (if more than 1 Group listed in the byline) and/or Subgroup (eg, Steering Committee)
Morgan	Hussey						
Mark	Hutchens		MD				
Tanya	Hutchins		FNP				
Julia	Hutter		MD				
Vincent	Huynh		BSc				
Jessica	Ibarra		MD				
Lilly	Immergluck		MD				
Dilek	Ince		MD				
Sarah M.	Ingalsbe-Geno		RPA-C				
Asha	Inniss		MS, APRN				
Farah	Irani		PhD				
Mashrur	Islam Majumder						
Ryan	Israelsen		MD				
Leslie	Iverson		PA				
Genevieve	Iwuala		FNP				
Lisa A.	Jackson		MD, MPH				
Peta-Gay	Jackson-Booth		MD				
Michael	Jacobs		MD				
Colleen	Jacobsen						
Jeffrey M.	Jacobson						
Jeffry	Jacqmein		MD				
Kate	Jaegle		MSN				
Shireen H.	Jaffer						
Maryam	Jahromi		MD				
Manish	Jain						
Frank	James		MD				
Holly	Janes		PhD				

Supplemental Online Content: Nonauthor Collaborators
*First name, last name, and suffix (if applicable) are required and will appear in PubMed.

***First Name and Middle Initial(s)**	***Last Name**	***Suffix (eg, Jr, III)**	Academic Degrees	Institution	Location (city, state/province, country)	Role or Contribution, eg, chair, principal investigator	Group (if more than 1 Group listed in the byline) and/or Subgroup (eg, Steering Committee)
Melanie	Jay						
Robert	Jeanfreau		MD				
Susan	Jeanfreau		MD				
William	Jennings		MD				
Natasa	Jenson		MD				
Keith R.	Jerome		MD, PhD				
Jennifer A.	Johnson		MD				
John	Johnson		MD				
Danyel	Johnson						
Diane H.	Johnson						
Maria	Johnson		MD				
Lisa	Johnson		FNP				
Erica	Johnson		PhD				
Kristen	Johnson						
Jennie	Johnson		MD				
Carrie	Johnston		MD, MS				
William H.	Jones		MD				
Kathleen S.	Jones						
Edward C.	Jones-López						
Bailey	Jordan						
Michal	Juraska		PhD				
Jarek	Juraszek		PhD				
Jessica E.	Justman						
Seyram	Kaali						
Scott	Kahney						
Spyros A.	Kalams		MD				
Satoshi	Kamidani		MD				
Sheetal	Kandiah		MD, MPH				
Pamela	Kane		DO				
Piush	Kanodia						
Shashi	Kapadia		MD, MS				

Supplemental Online Content: Nonauthor Collaborators
*First name, last name, and suffix (if applicable) are required and will appear in PubMed.

***First Name and Middle Initial(s)**	***Last Name**	***Suffix (eg, Jr, III)**	Academic Degrees	Institution	Location (city, state/province, country)	Role or Contribution, eg, chair, principal investigator	Group (if more than 1 Group listed in the byline) and/or Subgroup (eg, Steering Committee)
Lois	Katz						
Melinda	Katz						
Carol	Kauffman		MD				
Daniel	Kaul						
Margaret	Kautz						
Westly	Keating		PA-C				
Jia Jin	Kee		MS				
Michael C.	Keefer						
Wendy A.	Keitel		MD				
Colleen F.	Kelley		MD, MPH				
Amy	Kelley		BSN, RN				
Kary	Kelly		FNP-C				
Elizabeth J.	Kelly		PhD				
Sandra	Kelman						
Gerald	Kelty						
Ashley	Kennedy						
Christina	Kennelly		MD				
Maryam	Keshtkar-Jahromi		MD				
Jill	Kessler		MS				
Nzeera	Ketter						
Najat	Khan		PhD				
Nitin	Khandelwal						
Shishir K.	Khetan		MD				
Saadia	Khizer		MD, MPH				
Hannah	Kibuuka		MD				
Kristen	Kiersey		NP				
Paul	Kilgore		MD, MPH				
Anthony	Kim						
Kami	Kim		MD				
Murray A.	Kimmel		DO				

*First name, last name, and suffix (if applicable) are required and will appear in PubMed.

*First Name and Middle Initial(s)	*Last Name	*Suffix (eg, Jr, III)	Academic Degrees	Institution	Location (city, state/province, country)	Role or Contribution, eg, chair, principal investigator	Group (if more than 1 Group listed in the byline) and/or Subgroup (eg, Steering Committee)
Michelle	King		NP				
Jeffrey K.	Kingsley		DO				
William	Kirby		MD				
John A.	Kirby		MD				
Beth D.	Kirkpatrick		MD				
Judith L.	Kirstein		MD				
Cissy	Kityo Mutuluuza						
Noah	Kiwanuka						
Francis	Kiweewa						
John S.	Kizer		MD				
Terry D.	Klein		MD				
Thomas C.	Klein		MD				
Tracy R.	Klein		MD				
Jeffrey	Klein						
Susan	Kline		MD, MPH				
David	Kline		MD				
Jennifer	Knishinsky						
Jennifer	Knowles		BS				
Laura	Kogelman						
Sharon	Kohrs		RN				
Susan L.	Koletar						
James	Kopp						
Michael J.	Koren		MD				
Chalit	Kosolsak						
Karen L.	Kotloff		MD				
Angelica	Kottkamp						
Marguerite	Koutsoukos		MS				
Jayasree	Krishnankutty						
Mara	Kryvicky		PA-C				
James G.	Kublin		MD, MPH				

Supplemental Online Content: Nonauthor Collaborators
*First name, last name, and suffix (if applicable) are required and will appear in PubMed.

***First Name and Middle Initial(s)**	***Last Name**	***Suffix (eg, Jr, III)**	Academic Degrees	Institution	Location (city, state/province, country)	Role or Contribution, eg, chair, principal investigator	Group (if more than 1 Group listed in the byline) and/or Subgroup (eg, Steering Committee)
Kristine J.	Kucera		PA-C, MPAS, DHS				
Darshna	Kukian						
Marcy	Kulic		MD				
Rebecca J.	Kurnat						
Kelly	Kush						
Jitendra Singh	Kushwaha						
Mark E.	Kutner		MD				
Ben	Ladner						
Cathy	Laflan		MD				
Maryrose	Laguio-Vila						
Sherry	Lamberth		PharmD				
Raphael J.	Landovitz						
Dakotah C.	Lane		MD				
Thelma	Laot						
Anyela	Lara		MD				
Matthew	Laurens		MD				
Andrew	Lauria						
Daniel T.	Lawler		MD				
Nga	Le		PharmD				
Mathieu	Le Gars		PhD				
Vickie	Leathers						
Brett	Leav		MD				
Logan	Ledbetter		PA				
Julie E.	Ledgerwood		DO				
Kia	Lee						
Jessica L.	Lee						
Marina	Lee		PhD				
Angela	Lee						
Kia	Lee						

Supplemental Online Content: Nonauthor Collaborators
*First name, last name, and suffix (if applicable) are required and will appear in PubMed.

***First Name and Middle Initial(s)**	***Last Name**	***Suffix (eg, Jr, III)**	Academic Degrees	Institution	Location (city, state/province, country)	Role or Contribution, eg, chair, principal investigator	Group (if more than 1 Group listed in the byline) and/or Subgroup (eg, Steering Committee)
Mark	Leibowitz		MD				
Natalia	Leistner						
Mark	Lerman		MD				
Michael L.	Levin		MD				
Brendan	Levy						
Phillip	Levy		MD				
Wendell	Lewis	III	MD				
Michael	Lewis		MD				
Derek	Lewis		MD				
Wendell	Lewis		NP				
Maggie	Lewis						
Paola	Lichtenberger		MD				
Amanda	Lilienthal		MSN NP-C				
Kathleen	Linder		MD				
Carol Ann	Linebarger		MD				
Kendra	Lisec		PA				
Ryan	Little		FNP				
Susan J.	Little		MD				
Albert	Liu						
Anna S.	Lok						
Evelyn	Lomasney		MD				
James	Longe						
Carlos	Lopez		MD				
Eduardo	Lopez-Medina						
Julia	Lord		PA-C				
James	Lovell						
Ronald	Lubelchek						
John	Lucaj						
Njira	Lucia Lugogo						
Gary	Luckasen						

Supplemental Online Content: Nonauthor Collaborators
*First name, last name, and suffix (if applicable) are required and will appear in PubMed.

***First Name and Middle Initial(s)**	***Last Name**	***Suffix (eg, Jr, III)**	Academic Degrees	Institution	Location (city, state/province, country)	Role or Contribution, eg, chair, principal investigator	Group (if more than 1 Group listed in the byline) and/or Subgroup (eg, Steering Committee)
Gregg H.	Lucksinger		MD				
Annie	Luetkemeyer						
Benjamin J.	Luft		MD				
Rafael	Lupercio						
Alisha	Lutat						
Jill	Maaske		MD				
Rosa	MacBryde		RN				
Nolan	Mackey						
Rebecca P.	Madan						
Janine	Maenza						
Siham	Mahgoub		MD				
Mamodikoe	Makhene		MD, MPH				
Hussain	Malbari		MD				
Elissa	Malkin						
Carlos	Malvestutto						
Stephanie	Mamod						
Millie	Manalo						
Jose C.	Mancilla						
Kishorchandra	Mandaliya						
Somnath	Mangarule						
Padma N.	Mangu		MD				
Mary Beth	Manning		MD				
Rickey D.	Manning		MD				
Deb	Manzo		MSc				
Horacio	Marafioti		MD				
Kristin	Marks		MS, MD				
Mary A.	Marovich		MD				
Adrienna	Marquez						
Richard P.	Marshall		MD, PhD				
Judith	Martin		MD				
Troy	Martin						

Supplemental Online Content: Nonauthor Collaborators
*First name, last name, and suffix (if applicable) are required and will appear in PubMed.

***First Name and Middle Initial(s)**	***Last Name**	***Suffix (eg, Jr, III)**	Academic Degrees	Institution	Location (city, state/province, country)	Role or Contribution, eg, chair, principal investigator	Group (if more than 1 Group listed in the byline) and/or Subgroup (eg, Steering Committee)
Becky	Martinez		RN				
Reuben	Martinez						
Javier	Martínez						
Silvano Omar	Martínez Pérez		MD				
Catherine	Mary Healy		MD				
John	Mascola		MD				
Robin	Mason		MS, MBA				
Roger	Masotti		MS				
Paul G.	Matherne		MD				
Christopher	Matich						
Monica	Mauri						
Ryan C.	Maves						
Michelle	Maxwell		APRN				
Celia	Maxwell		MD				
Kenneth H.	Mayer						
Monica A.	McArthur		MD, PhD				
Jennifer	McCarter						
Michael J.	McCartney						
R. Scott	McClelland		MD, MPH				
Bruce	McClenathan		MD				
Rachael	McClennen						
Andrew	McConnell		BS				
Margaret E.	McCort						
M. Juliana	McElrath						
Charlene	McEvoy		MD, MPH				
Alice	McGarry						
John W.	McGettigan	Jr.	MD				
Jonlyn	McGettigan		MSN, RN				
Lora J.	McGill		MD				
Eric	McGrath		MD				

Supplemental Online Content: Nonauthor Collaborators
*First name, last name, and suffix (if applicable) are required and will appear in PubMed.

***First Name and Middle Initial(s)**	***Last Name**	***Suffix (eg, Jr, III)**	Academic Degrees	Institution	Location (city, state/province, country)	Role or Contribution, eg, chair, principal investigator	Group (if more than 1 Group listed in the byline) and/or Subgroup (eg, Steering Committee)
Mark M.	McKenzie		MD				
Cecilia	McKeown-Bragas		MD				
Irene	McKnight						
Morgan	McLaughlin		APRN				
Meredith	McNairy						
Kelly	McQuarrie		BSN				
Andrew	McWilliams		MD				
Jodi	Meck		MHA				
Surisday	Mederos						
Amy	Medina		ADN, BS				
Carmen	Medina		PA				
Jeffrey	Meier		MD				
Thomas	Mellman		MD				
Jessica M.	Mena		PA				
Lisa	Menard						
Kathleen	Menasche		LPN				
Janet	Mendez		APRN				
Jorge F.	Méndez Galván		MD				
Rafael	Mendoza		MD				
Erika	Mendoza		BA				
Sandra	Mendoza						
Rajan	Merchant		MD				
Fernando L.	Merino						
David	Metzger						
Jay	Meyer		MD				
Robert	Meyer						
Eric A.	Meyerowitz						
Nelson L.	Michael		MD				
Randle	Middleton						

Supplemental Online Content: Nonauthor Collaborators
*First name, last name, and suffix (if applicable) are required and will appear in PubMed.

*First Name and Middle Initial(s)	*Last Name	*Suffix (eg, Jr, III)	Academic Degrees	Institution	Location (city, state/province, country)	Role or Contribution, eg, chair, principal investigator	Group (if more than 1 Group listed in the byline) and/or Subgroup (eg, Steering Committee)
Maria	Mileno		MD				
Matthew	Miles						
Chelsea	Miles		NP				
Eric	Miller						
Jacqueline	Miller		MD				
Vicki E.	Miller		MD, MPH				
Meagan	Miller						
Paulina	Miller						
Jason	Milligan		MD				
Ann M.	Milliken						
Jill	Miracle						
Vincent	Mirkil		MD				
Mehdi	Mirsaeidi		MD				
Carol L.	Mitchell						
Kazuyuki	Mizuyama						
Gowdhami	Mohan		MD				
Satyajit	Mohapatra						
Sharzhaad	Molina Guizar						
Florence	Momplaisir						
Cynthia L.	Monaco						
Cynthia	Montano-Pereira						
Natasha	Montoya		APRN				
Candice	Montros						
Amaran	Moodley		MD				
Fernanda	Morales						
Caitlin	Moran		MD, MSc				
Catherine	Moreau						
Stephen	Morris						
Victoria	Mosteller		MD				
Stefanie	Mott		FNP-C				

Supplemental Online Content: Nonauthor Collaborators
*First name, last name, and suffix (if applicable) are required and will appear in PubMed.

***First Name and Middle Initial(s)**	***Last Name**	***Suffix (eg, Jr, III)**	Academic Degrees	Institution	Location (city, state/province, country)	Role or Contribution, eg, chair, principal investigator	Group (if more than 1 Group listed in the byline) and/or Subgroup (eg, Steering Committee)
Connie	Moya						
Terri	Moye						
Kimberly	Mueller		APRN				
Nancy	Mueller		PhD				
Jacqueline	Muenzner						
Nelly	Mugo						
Sauda	Muhammad						
Kathleen M.	Mullane		DO, PharmD				
Mark J.	Mulligan		MD				
Gysella	Muniz		MD				
Sigridh	Muñoz-Gómez						
Sonal	Munsiff						
Deborah	Murray		FNP				
Monica	Murray						
Linda	Murray		DO				
David B.	Musante		MD				
Sharon	Nachman		MD				
Shinya	Nagamatsu						
Sherif	Naguib		MD				
Jerome G.	Naifeh		MD				
S. Hasan	Naqvi		MD				
Marshall	Nash		MD				
Julio	Nasim		MD				
Martha	Nason		PhD				
Videlis	Nduba						
Paul	Nee						
Rosa	Negron		MD				
Marguerite	Neill		MD				
Joy	Nelson						

***First Name and Middle Initial(s)**	***Last Name**	***Suffix (eg, Jr, III)**	Academic Degrees	Institution	Location (city, state/province, country)	Role or Contribution, eg, chair, principal investigator	Group (if more than 1 Group listed in the byline) and/or Subgroup (eg, Steering Committee)
Taylor	Nelson		DO				
Tiffany	Nemecek						
Lori	Nesbitt						
Sonja	Neumeister		MPH				
Kathleen	Neuzil		MD				
Hannah	Nevarez						
Joseph L.	Newberg		MD				
Lauren	Newhouse						
Patrick	Newingham						
Fred E.	Newton		MD				
Lucy	Ng-Price		MA				
Kimberly	Nieves		AC-CRNP				
Chimeremma	Nnadi		MD, PhD				
Kimberly	Nofz		BSN				
Nicole L.	Nollen						
Asif	Noor						
Fernando	Noriega						
Richard M.	Novak		MD				
Patricia Nahirya	Ntege						
Paul J.	Nugent		DO				
Claudio	Nuñez Lagos						
Cheryl	Nuss		FNP				
Chelsie	Nutsch		MD				
Chelsie	Nutsch		NP				
Amesika	Nyaku		MD, MS				
Allison	O'Brien		AC-CRNP				
Enrique	Ocaña		MD				
Amanda	Occhino						
Kimberly	Offergeld		MSC				
Brenda	Okech						
Tracy	Okonya						

Supplemental Online Content: Nonauthor Collaborators
*First name, last name, and suffix (if applicable) are required and will appear in PubMed.

***First Name and Middle Initial(s)**	***Last Name**	***Suffix (eg, Jr, III)**	Academic Degrees	Institution	Location (city, state/province, country)	Role or Contribution, eg, chair, principal investigator	Group (if more than 1 Group listed in the byline) and/or Subgroup (eg, Steering Committee)
Jason F.	Okulicz						
Oyebisi	Olanrewaju		AC-CRNP				
Shelby	Olds		MD				
Marcia	O'Leary		RN				
Patrick A.	Oliver						
Odette	Olivieri		MD				
Jocelyn Zuleica	Olmos Vega		MD				
Sarah	Omodele						
Allie	Oplinger						
Emily	Oppenheim						
Yaa D.	Oppong		MD				
Jessica	Ortega						
Sandra Janeth	Ortega Domínguez						
Maricruz E.	Ortegón		MD				
Masaru	Oshita		MD				
Erica	Osmundsen						
Jaleh	Ostovar		FNP-C				
Jaleh	Ostovar		NP				
Quito	Osuna Carr		MD				
Maria	Otero						
Janet	Otto						
Samuel Gurrion	Ouma						
Jeffrey S.	Overcash		MD				
Temitope	Oyedele		MD				
Janet	Oyieko						
Janeth	Pacheco-Flores		MD				
Carmen A.	Paez		MD, MBA				
Barbara A.	Pahud		MD, MPH				
Ruth	Paiano		APRN				

Supplemental Online Content: Nonauthor Collaborators
*First name, last name, and suffix (if applicable) are required and will appear in PubMed.

***First Name and Middle Initial(s)**	***Last Name**	***Suffix (eg, Jr, III)**	Academic Degrees	Institution	Location (city, state/province, country)	Role or Contribution, eg, chair, principal investigator	Group (if more than 1 Group listed in the byline) and/or Subgroup (eg, Steering Committee)
Rolando	Pajon		PhD				
Yogesh K.	Paliwal		MD				
Amit	Paliwal		MD				
Steven	Palmer						
David	Pampe		MD				
Menelas N.	Pangalos		FMedSci, PhD				
Lalitha	Parameswaran						
Erwin	Pardo						
Mercedes	Paredes						
Purvi	Parikh						
Elizabeth	Parker		PA				
Susan	Parker						
Kevin	Parks		MD				
Reza	Parungao						
Alpa	Patel		MD				
Priti	Patel		NP				
Thomas	Patterson		MD				
Jan	Patterson		MD				
Walter	Patton		MD				
Leena	Paul		FNP				
Corrina	Pavetto		MS, RAC				
Juana R.	Pavie						
James	Peacock		MD				
Michele	Peake-Andrasik						
Laura	Pearlman		MS, MD, MBA				
Alyssa-Kay	Peay						
David	Pekala						

Supplemental Online Content: Nonauthor Collaborators
*First name, last name, and suffix (if applicable) are required and will appear in PubMed.

***First Name and Middle Initial(s)**	***Last Name**	***Suffix (eg, Jr, III)**	Academic Degrees	Institution	Location (city, state/province, country)	Role or Contribution, eg, chair, principal investigator	Group (if more than 1 Group listed in the byline) and/or Subgroup (eg, Steering Committee)
Alison	Pellecchia		BA				
Issac	Pena-Renteria						
Penny	Peng						
Noris	Peraita		ARNP				
Henry	Peralta						
Donna	Percy		RN, BSN				
Isabel	Pereira		MD				
Ashley	Perez						
James T.	Peterson		MD				
Jennifer	Petts						
Taryn	Petty		FNP				
Mai	Pham						
Sharine	Phan						
Amanda	Philyaw						
Sikhongi	Phungwayo						
Judy	Pi		PharmD				
Shelby	Pickle						
Paul	Pickrell		MD				
Terry	Piedra		BS				
Kristen K.	Pierce						
Daniela	Pineda Méndez		MD				
Ruth S.	Pinilla		MD				
Simmy	Pinto		MD				
Rebecca	Pitotti		RNP				
David	Pitrak		MD				
April	Pixler						
Stanford	Plavin		MD				
Laura	Polakowski		MD				
Terry L.	Poling		MD				

Supplemental Online Content: Nonauthor Collaborators
*First name, last name, and suffix (if applicable) are required and will appear in PubMed.

***First Name and Middle Initial(s)**	***Last Name**	***Suffix (eg, Jr, III)**	Academic Degrees	Institution	Location (city, state/province, country)	Role or Contribution, eg, chair, principal investigator	Group (if more than 1 Group listed in the byline) and/or Subgroup (eg, Steering Committee)
Philip	Ponce		MD				
Laura	Porterfield		MD				
Svitlana	Postol						
Amy	Potts		PA-C, MPH				
Richard	Powell		MD				
Margaret	Powers-Fletcher		PhD				
E. Javier	Pretell Alva						
Patty	Price-Abbott						
Veronica	Procasky		RN, JD				
John	Pullman		MD				
Lawrence J.	Purpura						
Marie-Laure	Py						
Evelyn	Quevedo		APRN				
Kim	Quillin						
Donald	Quinn		MD				
Jalima	Quintero		RN				
Vanessa	Raabe						
Frederick	Raiser	III	MD				
Sabina	Raja						
Vivek	Rajasekhar		DO				
Danielle	Raley		MD				
Mayur	Ramesh		MD				
Natalie	Ramirez						
Shelly	Ramirez						
Wanda	Ramon		BSPh				
James	Ramsey		NP-C				
Bruce G.	Rankin		DO				
Heather	Rattenbury-Shaw		DO				

*First name, last name, and suffix (if applicable) are required and will appear in PubMed.

***First Name and Middle Initial(s)**	***Last Name**	***Suffix (eg, Jr, III)**	Academic Degrees	Institution	Location (city, state/province, country)	Role or Contribution, eg, chair, principal investigator	Group (if more than 1 Group listed in the byline) and/or Subgroup (eg, Steering Committee)
Paulina	Rebolledo		MD				
Sergio E.	Recuenco						
Chinthaparthi Prabhakar	Reddy						
Patty	Reed						
Maria M.	Regalado		MD				
Malina	Regalado		NP				
María José	Reyes Fentanes		MD				
Humberto	Reynales		PhD				
Michele D.	Reynolds		MD				
Trina L.	Reynolds		BS				
Michelle	Reynolds						
Margaret	Rhee		MD				
Tamara	Richards						
Sha-Wanda	Richmond						
Sharon A.	Riddler						
Robert A.	Riesenberg		MD				
Dolores	Rijos						
Joshua	Rindt						
Enrique	Rivas						
Juan J.	Rivera		MD				
María I.	Rivera		MD				
Juana	Rivera		MD, MPH				
Gina	Rivero						
Rafael	Rivero						
Zainab	Rizvi						
Barbara	Rizzardi						
Barbara	Rizzardi		MD				
Denise	Roadman		PAC				
Merlin L.	Robb		MD				

Supplemental Online Content: Nonauthor Collaborators
*First name, last name, and suffix (if applicable) are required and will appear in PubMed.

***First Name and Middle Initial(s)**	***Last Name**	***Suffix (eg, Jr, III)**	Academic Degrees	Institution	Location (city, state/province, country)	Role or Contribution, eg, chair, principal investigator	Group (if more than 1 Group listed in the byline) and/or Subgroup (eg, Steering Committee)
Afsoon	Roberts						
Toni	Robinson		RN				
Dawn	Robison		APRN-C				
Elizabeth E.	Robison		BS				
Shannon	Rodman						
Jessica	Rodriguez		MD				
Carina A.	Rodriguez		MD				
Edith	Rodriguez						
Rachel	Rokser						
Charlotte-Paige	Rolle						
Stephanie	Rolsma		MD, PhD				
Peter	Ronco		BA				
Melody	Ronk		PA-C				
Adam	Rosen						
Jeffrey	Rosen						
David	Rosenberg		MD				
A. Lina	Rosengren		MD, MPH, MS				
Christiana A.	Rostad		MD				
Joan	Rothenberg		MD				
Rambod	Rouhbakhsh		MD, MBA				
Nadine	Rouphael		MD				
Crystal	Rowell		APRN, FNP-C				
Pavitra	Roychoudhury		PhD				
Rola G.	Rucker						
Monica	Ruiz						
Vida Veronica	Ruiz Herrera		MD				
Javier	Ruiz-Guiñazú		MD				
Guillermo M.	Ruiz-Palacios		MD				

Supplemental Online Content: Nonauthor Collaborators
*First name, last name, and suffix (if applicable) are required and will appear in PubMed.

***First Name and Middle Initial(s)**	***Last Name**	***Suffix (eg, Jr, III)**	Academic Degrees	Institution	Location (city, state/province, country)	Role or Contribution, eg, chair, principal investigator	Group (if more than 1 Group listed in the byline) and/or Subgroup (eg, Steering Committee)
Karen	Ruperto		MD				
Richard	Rupp		MD				
Sarah E.	Rutstein		MD, PhD				
Natasha	Rybak		MD				
Martin F.	Ryser		MD				
Jerald	Sadoff		MD				
Michelle	Saemann		RN				
Beth E.	Safirstein		MD				
Nafisa	Saleem		MD				
Jamshid	Saleh		MD				
Mansoor	Saleh						
Kacie	Sales		BSN, RN				
Amy	Salzl						
Denise	Sample						
Robert	Samuels						
Rosario	Sanchez						
Martha	Sanchez		MD				
Erica	Sanchez						
Pedro	Sánchez						
Laura Ruy	Sanchez Guerrero						
Nelia	Sanchez-Crespo		MD				
John	Sanders		MD, PhD				
Lisa J.	Sanders		MD				
Rica	Santiago						
Fredric	Santiago		MD				
María E.	Santolaya						
Nessryne	Sater						
Stephen	Savarino		MD				
Fredrick	Sawe						

Supplemental Online Content: Nonauthor Collaborators
*First name, last name, and suffix (if applicable) are required and will appear in PubMed.

***First Name and Middle Initial(s)**	***Last Name**	***Suffix (eg, Jr, III)**	Academic Degrees	Institution	Location (city, state/province, country)	Role or Contribution, eg, chair, principal investigator	Group (if more than 1 Group listed in the byline) and/or Subgroup (eg, Steering Committee)
Carolyn	Scatamacchia		MSN, NP-C				
Timothy W.	Schacker		MD				
Gert	Scheper		PhD				
Linda M.	Schipani						
Christy	Schmeck						
Robert	Schmidt		MD				
William	Schnitz		MD				
Florian	Schödel		MD, PhD				
Debra B.	Schroeck		MS, PA-C				
Lode	Schuerman		MD				
Hanneke	Schuitemaker		PhD				
Howard I.	Schwartz		MD				
Adam	Schwartz						
Howard	Schwartz		MD				
Jennifer	Schwartz		APRN				
Tiffany	Schwasinger-Schmidt						
Michael	Sciaudone		MD, MPH				
Hyman	Scott						
Tameika	Scott		DrPH				
Elizabeth	Secord		MD				
Nathan	Segall		MD				
William	Seger		MD				
Shannen	Seversen		PA-C				
Beverly E.	Sha						
Mitul	Shah		MD				
Jinen	Shah						
Samira	Shairi		RN				
Shivanjali	Shankaran						
Amie	Shannon		MD				

Supplemental Online Content: Nonauthor Collaborators
*First name, last name, and suffix (if applicable) are required and will appear in PubMed.

***First Name and Middle Initial(s)**	***Last Name**	***Suffix (eg, Jr, III)**	Academic Degrees	Institution	Location (city, state/province, country)	Role or Contribution, eg, chair, principal investigator	Group (if more than 1 Group listed in the byline) and/or Subgroup (eg, Steering Committee)
Adrienne E.	Shapiro						
Stephan C.	Sharp						
Marian E.	Shaw		MD				
Rozeli	Shelly		MD				
Sandra	Shelton						
Lawrence	Sher						
Amy	Sherman		MD				
Neil P.	Sheth		MD				
Steven	Shinn		MD				
Tammi L.	Shlotzhauer		MD				
Kathryn	Shoemaker		MS				
Timothy	Shope		MD, MPH				
Bo	Shopsin						
Steven	Shoptaw		PhD				
Rajeev	Shrestha						
Georgi	Shukarev		MD				
Farhan	Siddiqui						
Marc	Siegel						
Silva	Sieger						
Dianca	Sierra		BA				
Abraham	Siika						
William P.	Silver		MD				
Todd	Simmons		MD				
Gary	Simon						
Matthew D.	Sims						
Leonard	Singer		MD				
Chandramani	Singh						
Veer Bahadur	Singh						
Justin	Singletary						
Nuchra	Sirisuphmitr						
Stephanie	Skipper						

Supplemental Online Content: Nonauthor Collaborators
*First name, last name, and suffix (if applicable) are required and will appear in PubMed.

***First Name and Middle Initial(s)**	***Last Name**	***Suffix (eg, Jr, III)**	Academic Degrees	Institution	Location (city, state/province, country)	Role or Contribution, eg, chair, principal investigator	Group (if more than 1 Group listed in the byline) and/or Subgroup (eg, Steering Committee)
Stacy	Slechta		DO				
Teresa S.	Sligh		MD				
William B.	Smith		MD				
Mary	Smith		MSN, FNP-C				
Derek M.	Smith						
Michael J.	Smith						
Katherine	Smith		MD				
Candace	Smith		PharmD				
Kimberly	Snell		PharmD				
M. Mahdee	Sobhanie						
Magdalena E.	Sobieszczyk						
Obiageli	Sogbetun		MD, MPH				
Joel	Solis		MD				
Cora	Sonnier						
Carrie	Sopher						
Adriana	Sordo Duran						
Brit	Sovic						
Stephen A.	Spector		MD				
Steven	Sperber						
Bart	Spiessens		PhD				
Stephanie	Sproule		MMath				
Saranya	Sridhar		Dphil				
Jonathan	Staben		MD				
Jessica	Stahl						
Amber	Stanford		PA-C				
Lisa	Stangl		NP				
Ariana C.	Stanton		PA-C				
Katherine	Stapleton						
Jack	Stapleton		MD				
Thomas	Starkey						

Supplemental Online Content: Nonauthor Collaborators
*First name, last name, and suffix (if applicable) are required and will appear in PubMed.

***First Name and Middle Initial(s)**	***Last Name**	***Suffix (eg, Jr, III)**	Academic Degrees	Institution	Location (city, state/province, country)	Role or Contribution, eg, chair, principal investigator	Group (if more than 1 Group listed in the byline) and/or Subgroup (eg, Steering Committee)
Richard	Starlin		MD				
Amy	Starr		FNP-C				
Ryan P.	Starr		DO				
Kenneth	Steil		DO				
Carolene	Stephenson						
Kathryn E.	Stephenson		MD, MPH				
Stephanie	Sterling						
Lisa	Stevens Rameaka		MD				
Brittany	Stewart		RD, PharmD				
Jeffrey	Stoddard		MD				
Robert	Striker						
Frank	Struyf		MD				
Suzanna	Studdard						
Laura	Stulken		PA				
Mildred	Stull		APRN, FNP-C				
Rodney E.	Sturgeon		MD				
May-Yin	Suen						
Danny	Sugimoto		MD				
Amit	Suresh Bhate		MD				
Janice	Suyehira		MD				
Kazuo	Suzuki						
Shobha	Swaminathan		MD				
Suzanne	Swan		MD				
Edith	Swann		PhD				
Austin	Swanson						
Christy	Sweet						
Elisa	Swieboda		APRN				
Scott N.	Syndergaard		DO				

Supplemental Online Content: Nonauthor Collaborators
*First name, last name, and suffix (if applicable) are required and will appear in PubMed.

***First Name and Middle Initial(s)**	***Last Name**	***Suffix (eg, Jr, III)**	Academic Degrees	Institution	Location (city, state/province, country)	Role or Contribution, eg, chair, principal investigator	Group (if more than 1 Group listed in the byline) and/or Subgroup (eg, Steering Committee)
Karla Beatriz	Tafur Bances						
Therese	Takas		BS				
Zudi-Mwak	Takizala		MD, MPH, MBA				
Simbarashe G.	Takuva		MBChB, MSc				
Kawsar R.	Talaat						
Dipesh	Tamrakar						
Chen S.	Tan		MD				
Cayce	Tangemen						
Tamzin	Tanner		PhD				
Milagritos	Tapia		MD				
Denis	Tarakjian		MD				
Karen	Tashima		MD				
David	Tatelbaum						
Fernanda	Tavares Da-Silva						
Edward M.	Tavel	Jr.					
Kimberly L.	Taylor		PhD				
Brandie	Taylor		NP				
Barbara S.	Taylor		MD, MS				
David	Taylor						
Sharita	Tedder-Edwards		FNP				
Meghan	Teherani		MD				
Leslie	Tharenos						
Jeffrey	Thessing		MD				
Meagan	Thomas						
LaTeshia	Thomas-Seaton		MS, APRN				

Supplemental Online Content: Nonauthor Collaborators
*First name, last name, and suffix (if applicable) are required and will appear in PubMed.

***First Name and Middle Initial(s)**	***Last Name**	***Suffix (eg, Jr, III)**	Academic Degrees	Institution	Location (city, state/province, country)	Role or Contribution, eg, chair, principal investigator	Group (if more than 1 Group listed in the byline) and/or Subgroup (eg, Steering Committee)
Amy	Thompson						
Karl M.	Thompson		PhD				
Teri	Thompson-Seim						
Isaac	Thomsen		MD				
Hong V	Tieu						
Lucas Otieno	Tina						
Ramy J.	Toma		MD				
Joanne E.	Tomassini		PhD				
Christian	Tomaszewski						
Lee	Tomatsu						
Ryan	Tomlinson						
Xiaomi	Tong		PhD				
Tina	Tong		DrPH(c), MS, RAC(US)				
Juan P.	Torres						
Julian A.	Torres						
Dalia	Tovar		MA				
Carolyn	Tran		MD				
John J.	Treanor						
Carla	Truyers		PhD				
Sade	Tukuru						
Christine B.	Turley		MD				
Mark A.	Turner		MD				
Stefanie	Tyson						
Akiyoshi	Uchiyama						
Robert J.	Ulrich						
Lisa S.	Usdan		MD				
Gregory C.	Utz						
Timothy P.	Vachris		MD				

Supplemental Online Content: Nonauthor Collaborators

*First name, last name, and suffix (if applicable) are required and will appear in PubMed.

***First Name and Middle Initial(s)**	***Last Name**	***Suffix (eg, Jr, III)**	Academic Degrees	Institution	Location (city, state/province, country)	Role or Contribution, eg, chair, principal investigator	Group (if more than 1 Group listed in the byline) and/or Subgroup (eg, Steering Committee)
Bruno	Valenti		NP				
Insiya	Valika		PA-C				
Mimi	Van Der Leden		MD, PhD				
Ilse	Van Dromme		PhD				
Wesley	Van Ever						
Johan	Van Hoof		MD				
Griet	Van Roey		PhD				
An	Vandebosch		PhD				
Sergio L.	Vargas		MD				
Tilly	Varughese		MD				
Eduardo Gabriel	Vázquez Saldaña						
Norma	Vega						
Karen Sofia	Vega Orozco						
Diana	Vegas						
Hector	Velasquez						
Michele	Vertucci		PA, NP				
Veronica	Viar						
Roberto A.	Viau Colindres						
Nicholas A.	Viens		MD				
Tonya	Villafana		PhD, MPH				
Sandra M.	Villagómez Martínez		MD				
Marysol	Villegas						
Johan	Vingerhoets		PhD				
Mary	Vogler		MD				
Mandy	Vowell						
Keith W.	Vrbicky		MD				

Supplemental Online Content: Nonauthor Collaborators
*First name, last name, and suffix (if applicable) are required and will appear in PubMed.

***First Name and Middle Initial(s)**	***Last Name**	***Suffix (eg, Jr, III)**	Academic Degrees	Institution	Location (city, state/province, country)	Role or Contribution, eg, chair, principal investigator	Group (if more than 1 Group listed in the byline) and/or Subgroup (eg, Steering Committee)
Deo	Wabwire						
Larkin T.	Wadsworth	III	MD				
Tami	Wahlin		MD				
Anne	Wajja						
Paul H.	Wakefield		MD				
Anna	Wald		MD, MPH				
Shannon	Walker		MD				
James	Walker		RN				
Stephen R.	Walsh		MD				
Edward E.	Walsh						
Mary C.	Walsh						
Emmanuel B.	Walter						
Ayanna	Walters		RN, BSN				
Tina	Wang		MD				
Ashley	Warden						
T. Anh	Wartel		MD				
Michael	Waters		MD				
Breanz	Watkins						
Angella	Webb		APRN				
Hala	Webster						
Jessica L.	Weidler						
Thomas	Weiss		MD				
Margaret	Wells		MD				
Andrea	Wendrow						
Melody	Werne						
Whitney	West		APRN				
Jordan L.	Whatley		MD				
Jennifer A.	Whitaker		MD				
Julie	White		MBA				
Jewel J.	White		MD				
Toni	White						

Supplemental Online Content: Nonauthor Collaborators
*First name, last name, and suffix (if applicable) are required and will appear in PubMed.

***First Name and Middle Initial(s)**	***Last Name**	***Suffix (eg, Jr, III)**	Academic Degrees	Institution	Location (city, state/province, country)	Role or Contribution, eg, chair, principal investigator	Group (if more than 1 Group listed in the byline) and/or Subgroup (eg, Steering Committee)
Priyantha N.	Wijewardane		MD				
Timothy	Wilkin		MD, MPH				
Bethanie	Wilkinson		PhD				
Samantha	Williams		NP				
Barton G.	Williams		MD				
Hayes	Williams		MD, PhD				
Greg	Wilson		MD				
Christine	Wilson						
Natasha	Wilson		APRN				
Clint	Wilson		MD				
Jennifer	Winbigler		MD				
Peter J.	Winkle		MD				
Patricia L.	Winokur		MD				
John	Witbeck						
David	Wohl		MD				
Wayne	Woo		MS				
Leana	Woodside		DNP, NP-C				
Glenn	Wortman		MD				
Yi H.	Wu						
Paul E.	Wylie		MD				
Kinara S.	Yang						
Fan	Yang		MD				
Anelgine C.	Yoon		MD				
Jill	York						
Cheryl	Young						
Juan Luis	Yrivarren Giorza						
Joseph	Yusin						
Luz	Zapata		MD				

*First name, last name, and suffix (if applicable) are required and will appear in PubMed.

***First Name and Middle Initial(s)**	***Last Name**	***Suffix (eg, Jr, III)**	Academic Degrees	Institution	Location (city, state/province, country)	Role or Contribution, eg, chair, principal investigator	Group (if more than 1 Group listed in the byline) and/or Subgroup (eg, Steering Committee)
Zindy Yazmín	Zárate Hinojosa		MD				
Rebecca	Zash		MD				
Elodie	Zaworski						
Arthur L.	Zemanek						
Kate	Zenlea		MPH				
Karla	Zepeda		NP				
Marcus J.	Zervos		MD				
Nan	Zhai		NP-C				
Kevin	Zhang						
Nianxian	Zhang						
Honghong	Zhou		PhD				
James	Zhou		PhD, MS				
Andrea	Zimmer		MD				
Barry S.	Zingman						
Carmen D.	Zorrilla		MD				
