# Supplementary material for: Evaluating the Test-Negative Design for COVID-19 Vaccine Effectiveness Using Randomized Trial Data: A Secondary Cross-Protocol Analysis of 5 Randomized Clinical Trials
Source: JAMA Netw Open. 2025 May 28;8(5):e2512763. doi: 10.1001/jamanetworkopen.2025.12763 (PMC12120655; doi:10.1001/jamanetworkopen.2025.12763)
Supplement: Supplement 3. — Data Sharing Statement [file jamanetwopen-e2512763-s003.pdf]

Data Sharing Statement

Andrews. Evaluating the Test-Negative Design for COVID-19 Vaccine Effectiveness Using Randomized Trial Data. *JAMA Netw Open*. Published May 28, 2025.
doi:10.1001/jamanetworkopen.2025.12763

Data
Data available: Yes
Data types: Deidentified participant data
How to access data: Moderna COVE: Requests to use the data can be made to Moderna Inc., 200 Technology Square, Cambridge, MA 02139, USA. A materials transfer and/or data access agreement with the sponsor will be required for accessing of shared data. AstraZeneca AZD1222: AstraZeneca's data sharing policy is described at https://astrazenecagrouptrials.pharmacm.com/ST/Submission/Disclosure. AstraZeneca Group of Companies allows researchers to submit a request to access anonymized patient level clinical data, aggregate clinical or genomics data (when available), and anonymized clinical study reports through the Vivli web-based data request platform. Janssen ENSEMBLE: The data sharing policy of Janssen Pharmaceutical Companies of Johnson & Johnson is available at https://www.janssen.com/clinical-trials/ transparency. As noted on this site, requests for access to the study data can be submitted through Yale Open Data Access (YODA) Project site at http://yoda.yale.edu. Novavax PREVENT-19: Study information is available at https://clinicaltrials.gov/ct2/show/NCT04611802. Requests for the data supporting the results reported in this work should be submitted to the corresponding author (Leah I. B. Andrews; landrew2@uw.edu). Deidentified participant data may be provided. Sanofi/GSK VAT00008: Qualified researchers can request access to patient-level data and related study documents, including the clinical study report, study protocol with any amendments, blank case report forms, statistical analysis plan, and dataset specifications. Patient-level data will be anonymized, and study documents will be redacted to protect the privacy of trial participants. Further details on Sanofi's data sharing criteria, eligible studies, and process for requesting access can be found at https://vivli.org/.
When available: With publication

Supporting Documents
Document types: None

Additional Information
Who can access the data: Moderna COVE: Requests to use the data can be made to Moderna Inc., 200 Technology Square, Cambridge, MA 02139, USA. A materials transfer and/or data access agreement with the sponsor will be required for accessing of shared data. AstraZeneca AZD1222: AstraZeneca's data sharing policy is described at https://astrazenecagrouptrials.pharmacm.com/ST/Submission/Disclosure. AstraZeneca Group of Companies allows researchers to submit a request to access anonymized patient level clinical data, aggregate clinical or genomics data (when available), and anonymized clinical study reports through the Vivli web-based data request platform. Janssen ENSEMBLE: The data sharing policy of Janssen Pharmaceutical Companies of Johnson & Johnson is available at https://www.janssen.com/clinical-trials/ transparency. As noted on this site, requests for access to the study data can be submitted through Yale Open Data Access (YODA) Project site at http://yoda.yale.edu. Novavax PREVENT-19: Study information is available at https://clinicaltrials.gov/ct2/show/NCT04611802. Requests for the data supporting the results reported in this work should be submitted to the corresponding author (Leah I. B. Andrews; landrew2@uw.edu). Deidentified participant data may be provided. Sanofi/GSK VAT00008: Qualified researchers can request access to patient-level data and related study documents, including the clinical study report, study protocol with any amendments, blank case report

forms, statistical analysis plan, and dataset specifications. Patient-level data will be anonymized, and study documents will be redacted to protect the privacy of trial participants. Further details on Sanofi's data sharing criteria, eligible studies, and process for requesting access can be found at https://vivli.org/.

Types of analyses: Moderna COVE: Requests to use the data can be made to Moderna Inc., 200 Technology Square, Cambridge, MA 02139, USA. A materials transfer and/or data access agreement with the sponsor will be required for accessing of shared data. AstraZeneca AZD1222: AstraZeneca's data sharing policy is described at https://astrazenecagrouptrials.pharmacm.com/ST/Submission/Disclosure. AstraZeneca Group of Companies allows researchers to submit a request to access anonymized patient level clinical data, aggregate clinical or genomics data (when available), and anonymized clinical study reports through the Vivli web-based data request platform. Janssen ENSEMBLE: The data sharing policy of Janssen Pharmaceutical Companies of Johnson & Johnson is available at https://www.janssen.com/clinical-trials/ transparency. As noted on this site, requests for access to the study data can be submitted through Yale Open Data Access (YODA) Project site at http://yoda.yale.edu. Novavax PREVENT-19: Study information is available at https://clinicaltrials.gov/ct2/show/NCT04611802. Requests for the data supporting the results reported in this work should be submitted to the corresponding author (Leah I. B. Andrews; landrew2@uw.edu). Deidentified participant data may be provided. Sanofi/GSK VAT00008: Qualified researchers can request access to patient-level data and related study documents, including the clinical study report, study protocol with any amendments, blank case report forms, statistical analysis plan, and dataset specifications. Patient-level data will be anonymized, and study documents will be redacted to protect the privacy of trial participants. Further details on Sanofi's data sharing criteria, eligible studies, and process for requesting access can be found at https://vivli.org/.

Mechanisms of data availability: Moderna COVE: Requests to use the data can be made to Moderna Inc., 200 Technology Square, Cambridge, MA 02139, USA. A materials transfer and/or data access agreement with the sponsor will be required for accessing of shared data. AstraZeneca AZD1222: AstraZeneca's data sharing policy is described at https://astrazenecagrouptrials.pharmacm.com/ST/Submission/Disclosure. AstraZeneca Group of Companies allows researchers to submit a request to access anonymized patient level clinical data, aggregate clinical or genomics data (when available), and anonymized clinical study reports through the Vivli web-based data request platform. Janssen ENSEMBLE: The data sharing policy of Janssen Pharmaceutical Companies of Johnson & Johnson is available at https://www.janssen.com/clinical-trials/ transparency. As noted on this site, requests for access to the study data can be submitted through Yale Open Data Access (YODA) Project site at http://yoda.yale.edu. Novavax PREVENT-19: Study information is available at https://clinicaltrials.gov/ct2/show/NCT04611802. Requests for the data supporting the results reported in this work should be submitted to the corresponding author (Leah I. B. Andrews; landrew2@uw.edu). Deidentified participant data may be provided. Sanofi/GSK VAT00008: Qualified researchers can request access to patient-level data and related study documents, including the clinical study report, study protocol with any amendments, blank case report forms, statistical analysis plan, and dataset specifications. Patient-level data will be anonymized, and study documents will be redacted to protect the privacy of trial participants. Further details on Sanofi's data sharing criteria, eligible studies, and process for requesting access can be found at https://vivli.org/.
